# Supplementary material for: Synthetic phosphoethanolamine-modified oligosaccharides reveal the importance of glycan length and substitution in biofilm-inspired assemblies
Source: Nat Commun. 2022 Jul 8;13:3954. doi: 10.1038/s41467-022-31633-5 (PMC9270332; doi:10.1038/s41467-022-31633-5)
Supplement: Supplementary file 1 — Supplementary Info [file 41467_2022_31633_MOESM1_ESM.pdf]

Supplementary information

Synthetic phosphoethanolamine-modified oligosaccharides reveal the importance of glycan length and substitution in biofilm-inspired assemblies

Theodore Tyrikos-Ergas[1,2]†, Soeun Gim[1,2]†, Jhih-Yi Huang[1,2]†, Sandra Pinzón Martín[1,2], Daniel Varón Silva[2,3], Peter H. Seeberger[1,2] & Martina Delbianco[1]*

[1]Department of Biomolecular Systems, Max Planck Institute of Colloids and Interfaces, Am Mühlenberg 1, 14476 Potsdam, Germany.

[2]Department of Chemistry and Biochemistry, Freie Universität Berlin, Arnimallee 22, 14195 Berlin, Germany.

[3]Current affiliation: University of Applied Sciences and Arts Northwestern Switzerland, School of Life Sciences, Institute of Chemistry und Bioanalytics, Hofackerstrasse 30, 4132 Muttenz, Switzerland.

† These authors contributed equally

*Corresponding author. E-mail: martina.delbianco@mpikg.mpg.de (M.D.)

Table of contents

Table of contents 2

1. Supplementary Notes 4

2. Supplementary Methods 5

2.1. Synthesis of oligosaccharides 5

2.2. Building blocks and photolabile linkers 6

2.3. Automated glycan assembly (AGA) 7

2.3.1. General materials and methods 7

2.3.2. Preparation of stock solutions 7

2.3.3. Modules for automated synthesis 7

2.4. Post-synthesizer manipulations 12

2.5. Synthesis of P 14

2.6. Synthesis of PA 18

2.7. Synthesis of PAP 22

2.8. Synthesis of PA_5 26

2.9. Synthesis of A_3PA_2 30

2.10. Synthesis of $(APA)_2$ 34

2.11. Synthesis of $A_2P_2A_2$ 38

2.12. Synthesis of P_2APA_2 42

2.13. Synthesis of $(PA)_3$ 46

2.14. Synthesis of R5 50

3. Supplementary Discussion 52

3.1. Assembly of artificial fibers 52

3.2. Fibrils structural analysis 53

3.3. NMR sample preparation 62

3.4. Proton assignment and structural characterization of R5. 63

3.5. NMR characterization of R5 and A_6. 66

3.6. NMR characterization of R5 and $(PA)_3$. 67

3.7. NMR characterization of R5 and P_2APA_2. 68

3.8. NMR comparison of the four samples. 69

3.9. ^{31}P NMR of $(PA)_3$ and $(PA)_3$ in presence of R5 70

3.10. Analysis of the mechanical properties of the artificial biofilms-inspired matrices 71

4. Supplementary References 74

1. Supplementary Notes

All chemicals used were reagent grade and used as supplied unless otherwise noted. The automated syntheses were performed on a home-built synthesizer developed at the Max Planck Institute of Colloids and Interfaces. Analytical thin-layer chromatography (TLC) was performed on Merck silica gel 60 F254 plates (0.25 mm). Compounds were visualized by UV irradiation or dipping the plate in a staining solution (sugar stain: 10% H_2SO_4 in EtOH; CAM: 48 g/L ammonium molybdate, 60 g/L ceric ammonium molybdate in 6% H_2SO_4 aqueous solution). Flash column chromatography was carried out by using forced flow of the indicated solvent on Fluka Kieselgel 60 M (0.04 – 0.063 mm). Analysis and purification by normal and reverse phase HPLC was performed by using an Agilent 1200 series. Products were lyophilized using a Christ Alpha 2-4 LD plus freeze dryer. ^{1}H, ^{13}C and HSQC NMR spectra were recorded on a Varian 400-MR (400 MHz), a Varian 600-MR (600 MHz) or a Varian 700-MR (700 MHz) spectrometer. Spectra were recorded in $CDCl_3$ by using the solvent residual peak chemical shift as the internal standard ($CDCl_3$: 7.26 ppm ^{1}H, 77.0 ppm ^{13}C) or in D_2O using the solvent as the internal standard in ^{1}H NMR (D_2O: 4.79 ppm ^{1}H). High resolution mass spectra were obtained using a 6210 ESI-TOF mass spectrometer (Agilent) and a MALDI-TOF autoflex™ (Bruker). MALDI and ESI mass spectra were run on IonSpec Ultima instruments. Scanning electron microscopy (SEM) images were obtained with a Gemini SEM, LEO 1550 system with cold field emission gun operation at 3 kV. All the samples were coated with Au/Pd. Transmission electron microscopy (TEM) images were obtained on carbon-coated copper grids with a Zeiss EM 912Ω instrument at 120 kV without negative staining. Circular dichroism (CD) spectra were acquired with a Chrascan qCD spectrometer (Applied Photophysics Ltd. Leatherhead, UK) using a quartz cuvette (Helma GmbH & Co. KG, Mullheim, Germany) at 23°C with a band width of 1 nm. The thioflavin T (ThT) fluorescence was measured at RT using a SpectraMax M5 plate reader (Molecular Devices LLC., California, USA) with an excitation wavelength at 438 nm with a cut-off filter at 475 nm. The 5 day-matured samples were incubated with the ThT solution with the final concentration of 20 µM for 30 minutes and stirred 10 seconds right before the measurement. Atomic force microscopy (AFM) was carried out in air with a JPK NanoWizard 4 AFM. Images were attained with the conventional AC mode and flattened without further modification. The samples for AFM for the imaging of single filaments (Day 1) and matured fibrils (Day 5) were prepared on freshly cleaved mica. Qualitative imaging (QI) mode was applied for nanoindentation and adhesion force measurement with a silicon cantilever. For mechanical property analysis, the uniform film with the thickness of 300 nm were prepared on a pre-washed glass substrate. The nanoindentation was conducted in air with a silicon nitride cantilever at a constant force of 10 nN. The approaching force-distance curve were fit to the Hertz model and manipulated to obtain Young's modulus. A tipless cantilever was modified with a polystyrene bead (diameter, 8.4 µm) for the adhesion measurement. 10 x 10 curves were obtained from one spot. A minimum of three samples with 10 spots per sample was tested for each oligosaccharide. JPK data processing software was used to analyze all AFM data including images and forces. All solution-state NMR experiments regarding **R5** and its interaction with oligosaccharides were performed using a Bruker Ascend™ (AvanceIII HD) 700 MHz NMR spectrometer with water suppression. All spectra were recorded at 297 K. In order to monitor the aggregation of R5, ^{1}H spectra were acquired on Day 0, Day 1, and Day 5. Data were processed with MestReNova.

2. Supplementary Methods

2.1. Synthesis of oligosaccharides

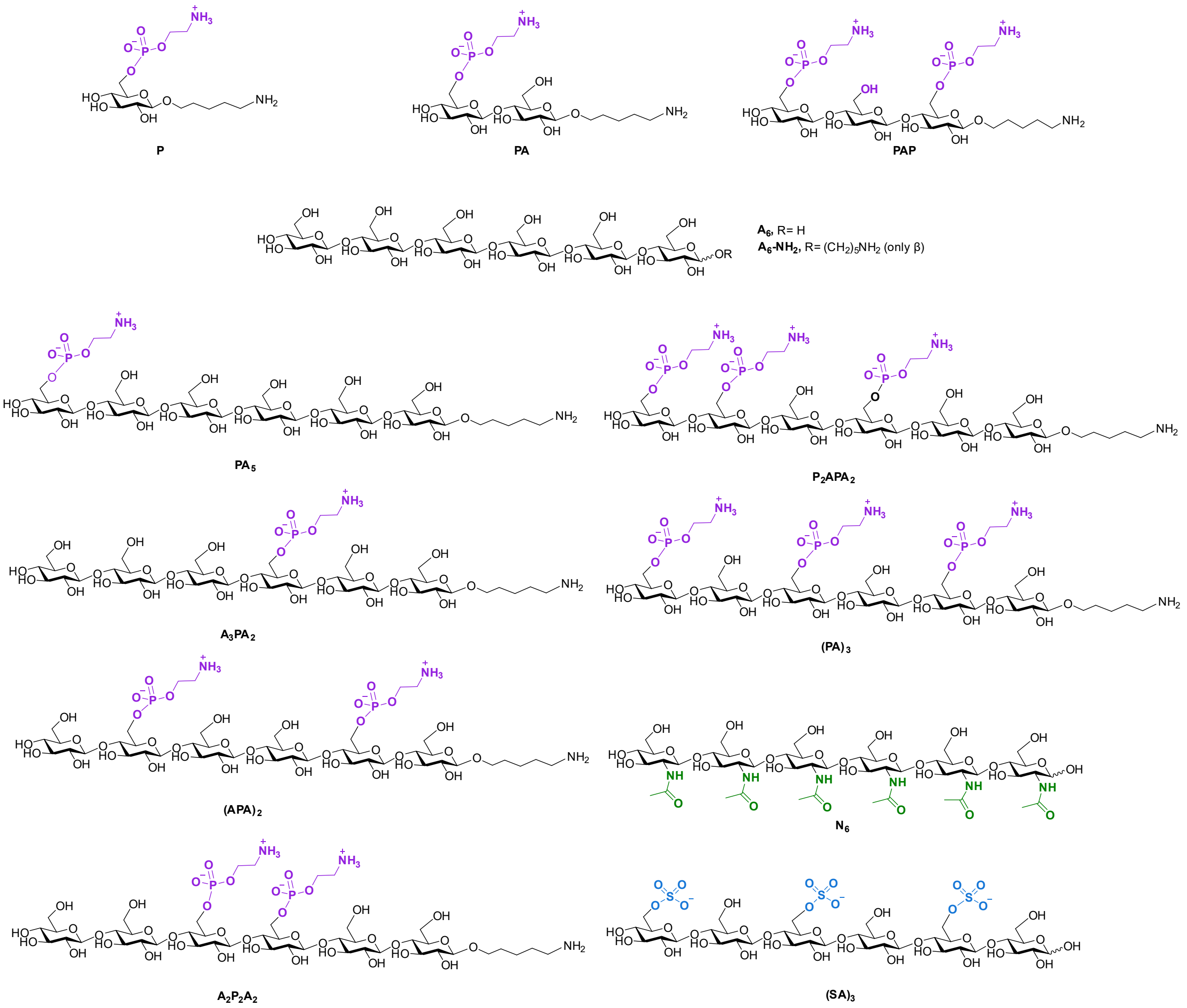

Supplementary Figure 1. Chemical structure of the oligosaccharides used in the study. **A_6, A_6-NH_2, N_6** and **$(SA)_3$** were prepared following previously reported procedures.[1,2]

2.2. Building blocks and photolabile linkers

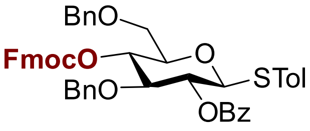

BB1

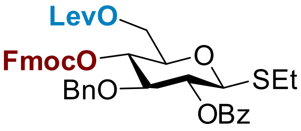

BB2

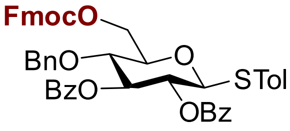

BB3

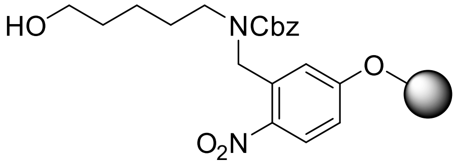

L1

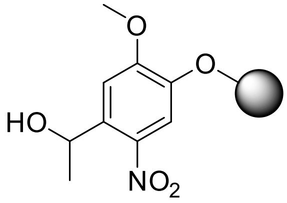

L2

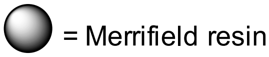

BB1 and **BB3** were purchased from GlycoUniverse (Germany, product codes Glc32.11021402 and Glc32.11110214 respectively). **BB2** was synthesized following previous established procedures[3]. Merrifield resin equipped with photocleavable linkers (**L1**, loading 0.30 mmol/g and **L2**, loading 0.34 mmol/g) were prepared according to previous literature.[4]

2.3. Automated glycan assembly (AGA)

2.3.1. General materials and methods

The automated syntheses were performed on a home-built synthesizer designed at the Max Planck Institute of Colloids and Interfaces. All solvents used were HPLC-grade. The solvents used for the building block, activator, TMSOTf and capping solutions were taken from an anhydrous solvent system (*J*cmeyer-solvent systems). The building blocks were co-evaporated three times with toluene and dried for 1 h under high vacuum before use. Activator, capping, deprotection, acidic wash and building block solutions were freshly prepared and kept under argon during the automation run. All yields of products obtained by AGA were calculated on the basis of resin loading. Resin loading was determined following previously established procedures.[5]

2.3.2. Preparation of stock solutions

- **Building block solution**: 0.08 mmol of building block was dissolved in DCM (1 mL).
- **Activator solution**: 1.35 g of recrystallized NIS was dissolved in 40 mL of a 2:1 mixture of anhydrous DCM and anhydrous dioxane. Then triflic acid (55 µL) was added. The solution is kept at 0 ˚C for the duration of the automation run.
- **Fmoc deprotection solution**: A solution of 20% piperidine in DMF (v/v) was prepared.
- **Lev deprotection solution**: Hydrazine acetate (550 mg, 5.97 mmol) was dissolved in pyridine/AcOH/H2O (40 mL, v/v, 32:8:2) and sonicated at RT for 10 min.
- **TMSOTf solution**: TMSOTf (0.45 mL, 2.49 mmol) was added to DCM (40 mL).
- **Capping solution**: A solution of 10% acetic anhydride and 2% methanesulfunic acid in DCM (v/v) was prepared.

2.3.3. Modules for automated synthesis

Module A: Resin Preparation for Synthesis (20 min)

All automated syntheses were performed on 0.0125 mmol scale. Resin was placed in the reaction vessel and swollen in DCM for 20 min at RT prior to synthesis. During this time, all reagent lines needed for the synthesis were washed and primed. After swelling, the resin was washed with the DMF, THF, and DCM (three times each with 2 mL for 25 s).

Module B: Acidic Wash with TMSOTf Solution (20 min)

The resin was swollen in 2 mL DCM and the temperature of the reaction vessel was adjusted to -20 °C. Upon reaching the desired temperature, TMSOTf solution (1 mL) was added dropwise to the reaction vessel. After bubbling for 3 min, the acidic solution was drained and the resin was washed with 2 mL DCM for 25 s.

Supplementary Table 1. Sequences of action executed in Module B.

Action	Cycles	Solution	Amount	T (°C)	Incubation time
**Cooling**	-	-	-	-20	(15 min)*
**Deliver**	1	DCM	2 mL	-20	-
**Deliver**	1	TMSOTf solution	1 mL	-20	3 min
**Wash**	1	DCM	2 mL	-20	25 sec

*Time required to reach the desired temperature.

Module C: Thioglycoside Glycosylation (35 min)

The building block solution (0.08 mmol of BB in 1 mL of DCM per glycosylation) was delivered to the reaction vessel. After the set temperature was reached, the reaction was started by drop wise addition of the activator solution (1.0 mL, excess). The glycosylation conditions are building block dependent[1,6] (we report the most common set of conditions). After completion of the reaction, the solution was drained and the resin was washed with DCM, DCM:dioxane (1:2, 3 mL for 20 s) and DCM (two times, each with 2 mL for 25 s). The temperature of the reaction vessel was increased to 25 °C for the next module.

Supplementary Table 2. Sequences of action executed in Module C.

Action	Cycles	Solution	Amount	T (°C)	Incubation time
**Cooling**	-	-	-	-20	-
**Deliver**	1	BB solution	1 mL	-20	-
**Deliver**	1	Activator solution	1 mL	-20	-
**Reaction** (BB dependent)	1			-20<br>to 0	5 min<br>20 min
**Wash**	1	DCM	2 mL	0	5 sec
**Wash**	1	DCM : Dioxane (1:2)	2 mL	0	20 sec
**Heating**	-	-	-	25	-
**Wash**	2	DCM	2 mL	> 0	25 sec

Module D: Capping (30 min)

The resin was washed with DMF (two times with 2 mL for 25 s) and the temperature of the reaction vessel was adjusted to 25 °C. 2 mL of pyridine solution (10% in DMF) was delivered into the reaction vessel. After 1 min, the solution was drained and the resin washed with DCM (three times with 3 mL for 25 s). 4 mL of capping solution was delivered into the reaction vessel. After 20 min, the solution was drained and the resin washed with DCM (three times with 3 mL for 25 s).

Supplementary Table 3. Sequences of action executed in Module D.

Action	Cycles	Solution	Amount	T (°C)	Incubation time
**Heating**	-	-	-	25	(5 min)*
**Wash**	2	DMF	2 mL	25	25 sec
**Deliver**	1	10% Pyridine in DMF	2 mL	25	1 min
**Wash**	3	DCM	2 mL	25	25 sec
**Deliver**	1	Capping Solution	4 mL	25	20 min
**Wash**	3	DCM	2 mL	25	25 sec

*Time required to reach the desired temperature.

Module E1: Fmoc Deprotection (9 min)

The resin was washed with DMF (three times with 2 mL for 25 s) and the temperature of the reaction vessel was adjusted to 25 °C. 2 mL of Fmoc deprotection solution was delivered to the reaction vessel and kept under Ar bubbling. After 5 min, the solution was drained and the resin washed with DMF (three times with 3 mL for 25 s) and DCM (five times each with 2 mL for 25 s). The temperature of the reaction vessel was decreased to -20 °C for the next module.

Supplementary Table 4. Sequences of action executed in Module E1.

Action	Cycles	Solution	Amount	T (°C)	Incubation time
**Wash**	3	DMF	2 mL	25	25 sec
**Deliver**	1	Fmoc depr. solution	2 mL	25	5 min
**Wash**	1	DMF	2 mL		
**Cooling**	-	-	-	-20	-
**Wash**	3	DMF	2 mL	< 25	25 sec
**Wash**	5	DCM	2 mL	< 25	25 sec

Module E2: Lev Deprotection (65 min)

The resin was washed with DMF (three times with 2 mL for 25 s), DCM (1.3 mL) was delivered to the reaction vessel and the temperature of the reaction vessel was adjusted to 25 °C. 2 mL of Lev deprotection solution was delivered to the reaction vessel and kept under pulsed Ar bubbling for 30 min. This procedure was repeated twice. The solution was drained and the resin washed with DMF (three times with 3 mL for 25 s) and DCM (five times each with 2 mL for 25 s).

Supplementary Table 5. Sequences of action executed in Module E2.

Action	Cycles	Solution	Amount	T (°C)	Incubation time
**Wash**	3	DMF	2 mL	25	25 sec
**Deliver**	2	Lev depr. solution	2 mL	25	30 min
**Wash**	1	DMF	2 mL		
**Cooling**	-	-	-	-20	-
**Wash**	3	DMF	2 mL	< 25	25 sec
**Wash**	5	DCM	2 mL	< 25	25 sec

2.4. Post-synthesizer manipulations

Module F: Cleavage from solid Support

The oligosaccharides were cleaved from the solid support using a continuous-flow photoreactor as described previously.[7]

Module G: Phosphorylation

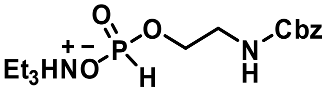

4

4 was prepared according to a previously established procedure.[8]

The partially protected oligosaccharide obtained from *Module F* was mixed with **4** (4 equiv.), co-evaporated with pyridine for three times and dried under high vacuum for 2 h. The ratio between the oligosaccharide and **4** changed depending on the oligosaccharide structure (here we report the most common set of conditions, variations are reported in the specific procedures). The mixture was dissolved in anhydrous pyridine (2 mL) and a solution of pivaloyl chloride (equimolar to **4**) in pyridine (1 mL) was added. The solution was stirred for 12 h at RT, after which time iodine (10 equiv.) and water (0.5 mL) were added and the reaction was stirred for additional 2 h. The reaction mixture was quenched with $Na_2S_2O_3$ and extracted with CH_2Cl_2. The organic layers were combined and evaporated.

Module H: Solution-phase methanolysis

The oligosaccharide was dissolved in MeOH : DCM (1.5 mL, 1:1). NaOMe in MeOH (0.5 M, 3 equiv. per benzoyl ester) was added and the solution was stirred at RT for 12 h, neutralized with Amberlite IR-120 (H^+ form), filtered and concentrated *in vacuo.*

Module I: Hydrogenolysis at ambient pressure[a]

The crude compound obtained from Module H was dissolved in 2 mL of *t*-BuOH:H_2O (1:1). The Pd catalyst (2.5 times the weight of the starting material) was added and the reaction was stirred in a flask equipped with a H_2 balloon. The reaction progress was monitored to avoid undesired side products formation. Upon completion, the reaction was filtered and washed with *t*-BuOH and H_2O. The filtrates were concentrated *in vacuo.*

[a]Reaction times and type of catalyst are indicated for each synthesis.

Module J: Purification

After photovleavage, crudes were analyzed and purified using analytical and preparative HPLC (Agilent 1200 Series spectrometer, **Method A_1** and **Method A_2,** respectively). The protected phosphorylated crudes were purified with **Method B** and **Method C**. After methanolysis, the semi-deprotected compounds were purified with **Method C**. The final compounds were purified with **Method E** and analyzed using analytical HPLC (Agilent 1200 Series spectrometer, **Method F**).

- **Method A_1:** (YMC-Diol-300 column, 150 x 4.6 mm) flow rate of 1.0 mL / min with Hex – 20% EtOAc as eluent [isocratic 20% EtOAc (5 min), linear gradient to 55% EtOAc (35 min), linear gradient to 100% EtOAc (5 min)].
- **Method A_2:** (YMC-Diol-300 column, 150 x 20 mm) flow rate of 15 mL / min with Hex – 20% EtOAc as eluent [isocratic 20% EtOAc (5 min), linear gradient to 55% EtOAc (35 min), linear gradient to 100% EtOAc (5 min)].
- **Method B**: Manual silica gel column chromatography quenched with 0.1% Et_3N in DCM. Solvent system DCM:MeOH, gradient from 0 to 10% MeOH.
- **Method C**: Sephadex® LH-20 column with DCM:MeOH (1:1) as eluent, isocratic.
- **Method D:** Sephadex® LH-20 column with H_2O:MeOH (1:1) as eluent, isocratic.
- **Method E:** Manual reverse phase C_{18} silica gel column chromatography. Solvent system H_2O:MeOH, gradient from 0 to 50% MeOH).
- **Method F:** (Hypercarb column, 150 x 10 mm) flow rate of 0.7 mL / min with H_2O (0.1% formic acid) as eluents [isocratic (5 min), linear gradient to 50% ACN (30 min), linear gradient to 100% ACN (5 min)].

Following final purification, all deprotected products were lyophilized on a Christ Alpha 2-4 LD plus freeze dryer prior to characterization.

2.5. Synthesis of P

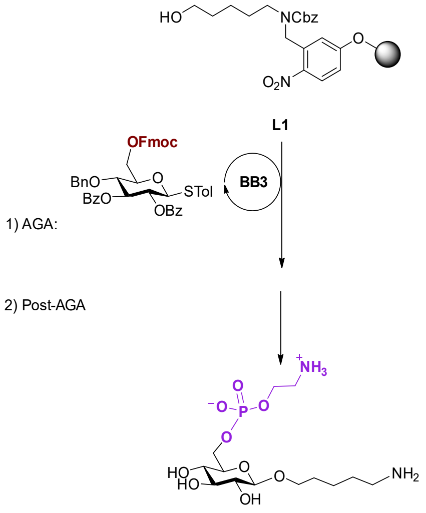

P

Supplementary Table 6. Synthesis of **P**.

Step		Modules	Notes
AGA	**BB3**	**A**<br>**B, C, D, E1**	**C:** (**BB3**, -20 °C for 5 min, 0 °C for 20 min)
Post-AGA		**F, J**	**J:** (Method **A_2**, t_R = 15.9 min)
		**G, J1, J2,**	**G:** (4 equiv. of **4**)<br>**J1:** (Method **B**)<br>**J2:** (Method **C**)
		**H,J1, I, J2**	**J1:** (Method **C**) **I:** 10% Pd/C (12 h) **J2:** (Method **E** and **D**)

Compound **P** was obtained as a white solid (1.1 mg, 21 % overall yield).

Analytical data for **P**: ^{1}H NMR (400 MHz, D_2O) δ 4.40 (t, *J* = 7.7 Hz, 1H), 4.17 – 3.95 (m, 4H), 3.85 (dt, *J* = 10.0, 6.5 Hz, 1H), 3.75 – 3.35 (m, 4H), 3.27 (s, 1H), 3.25 – 3.12 (m, 3H), 2.93 (t, *J* = 7.5 Hz, 2H), 1.60 (p, *J* = 7.5 Hz, 4H), 1.50 – 1.29 (m, 2H); ^{13}C NMR (101 MHz, D_2O) δ 102.19, 75.54, 74.2, 72.97, 70.07, 68.95, 64.47, 40.04, 39.22, 28.06, 26.27, 21.93; ^{31}P NMR (162 MHz, D_2O) δ 0.24; m/z (HRMS+) 389.1671 $[M+H]^+$ ($C_{13}H_{30}N_2O_9P$ requires 389.1689).

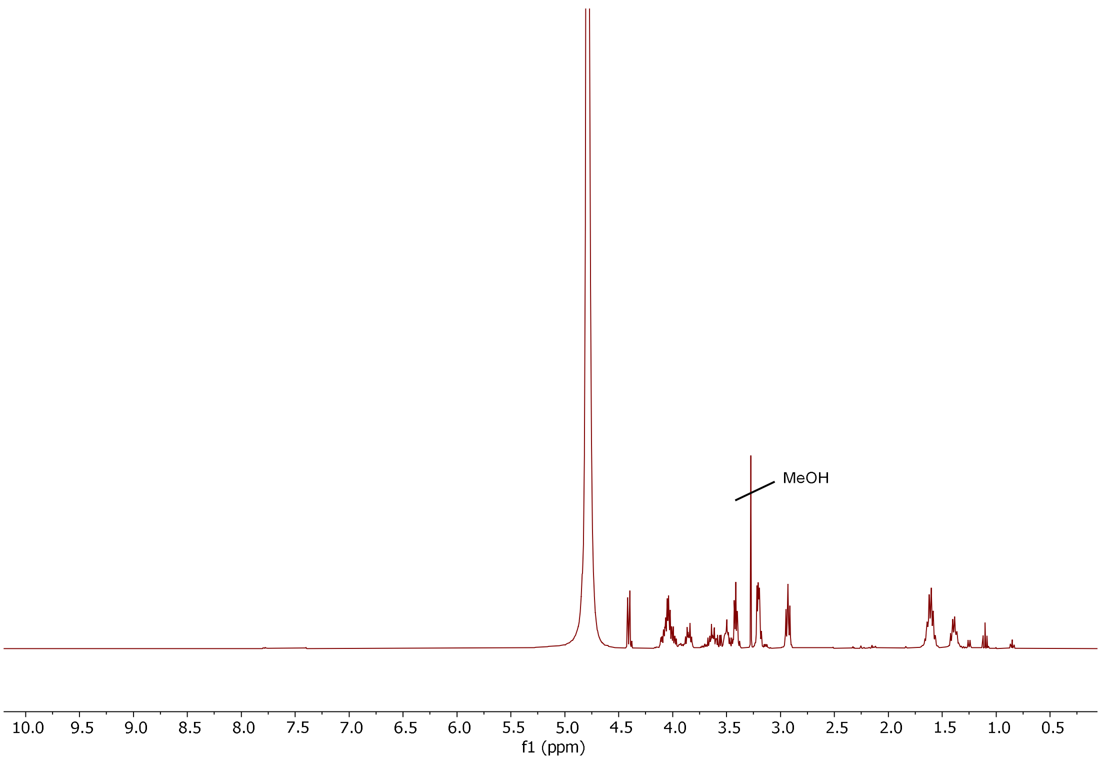

Supplementary Figure 2. ^{1}H NMR of P (400 MHz, D_2O)

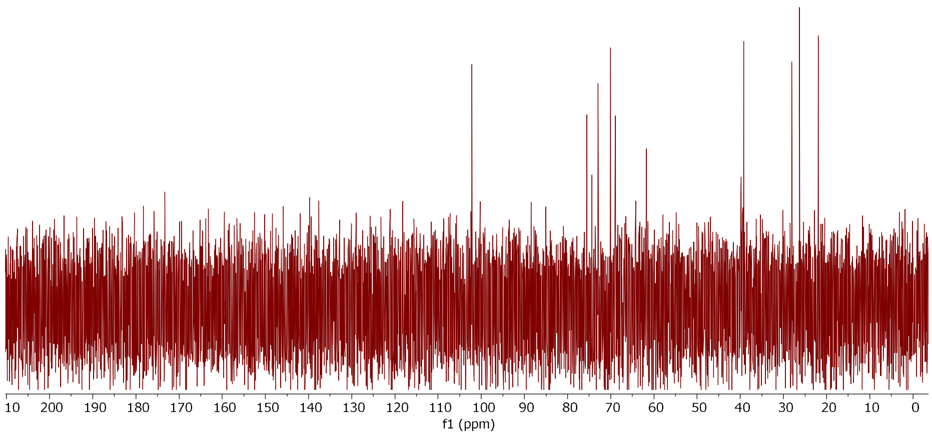

Supplementary Figure 3. ^{13}C NMR of P (101 MHz, D_2O)

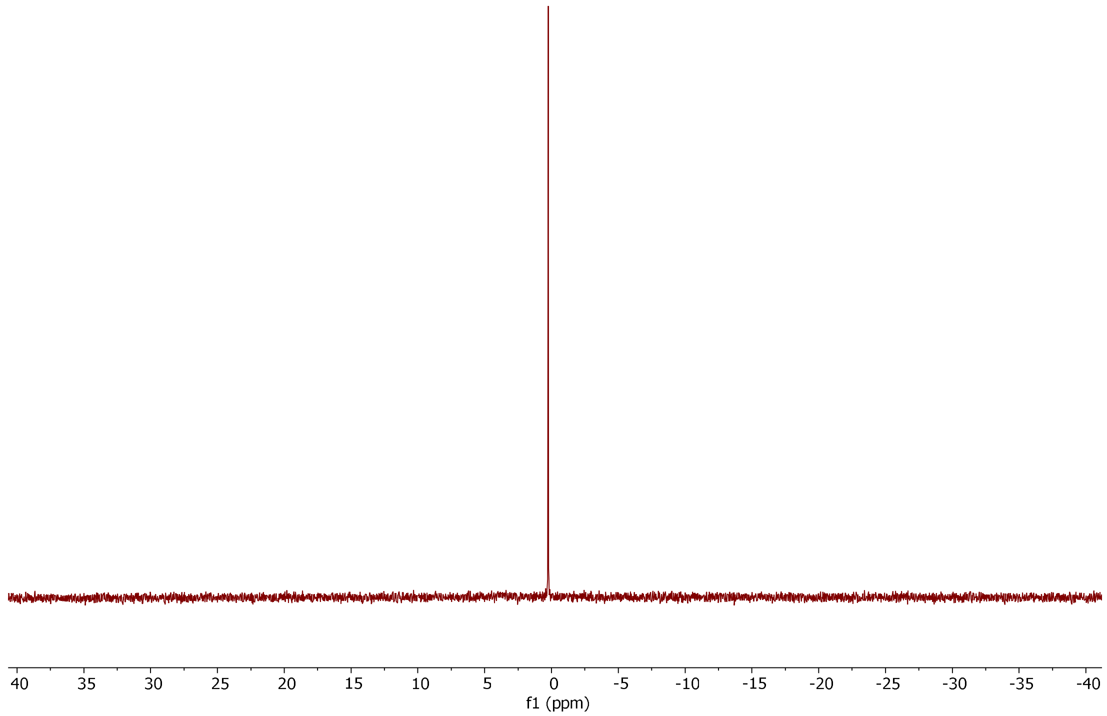

Supplementary Figure 4. ^{31}P NMR of P (162 MHz, D_2O)

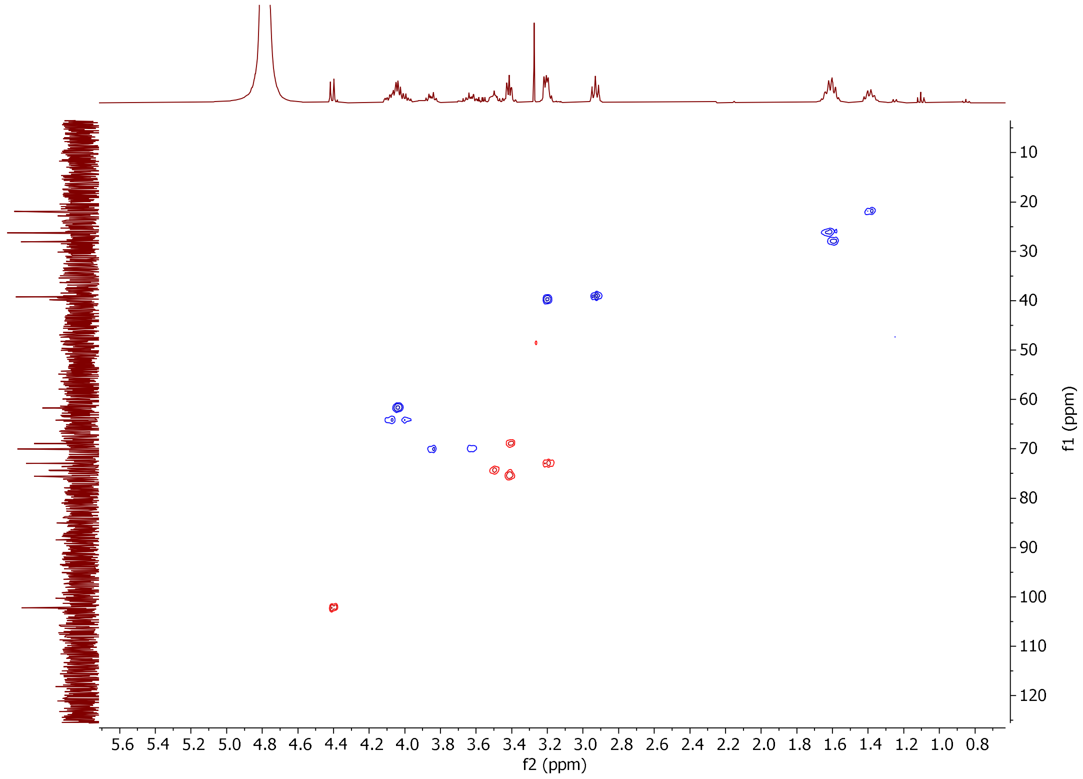

Supplementary Figure 5. HSQC NMR of P (D_2O)

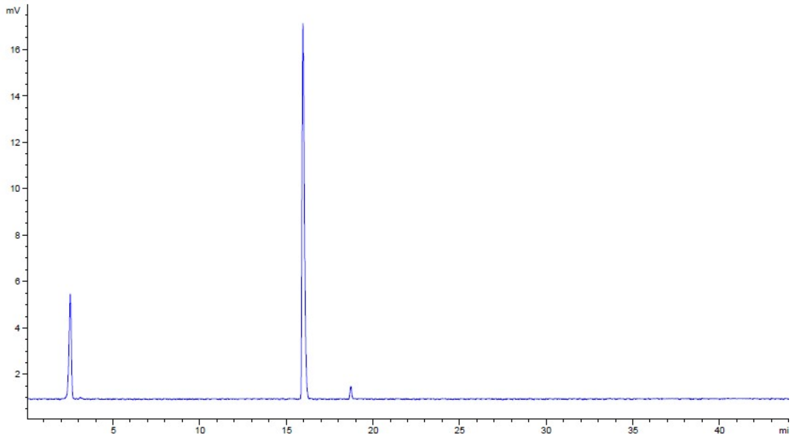

Supplementary Figure 6. RP-HPLC of P (ELSD trace, Method F, t_R= 15.9 min)

2.6. Synthesis of PA

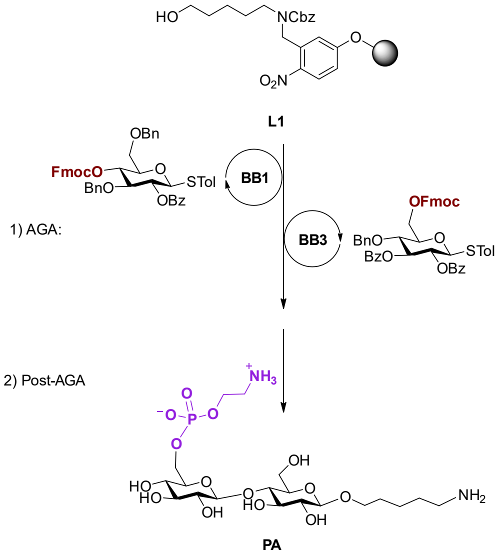

Supplementary Table 7. Synthesis of **PA**.

Step		Modules	Notes
AGA		**A**	
	**BB1**	**B, C, D, E1**	**C:** (**BB1**, -20 °C for 5 min, 0 °C for 20 min)
	**BB3**	**B, C, D, E1**	**C:** (**BB3**, -20 °C for 5 min, 0 °C for 20 min)
Post-AGA		**F, J**	**J:** (Method **A$_2$**, t_R = 16.3 min)
		**G, J1, J2,**	**G:** (4 equiv. of **4** per -OH)<br>**J1:** (Method **B**)<br>**J2:** (Method **C**)
		**H,J1, I, J2**	**J1:** (Method **C**) **I:** 10% Pd/C (12 h) **J2:** (Method **E** and **D**)

Compound **PA** was obtained as a white solid (2.1 mg, 28 % overall yield).

Analytical data for **PA**: ^{1}H NMR (400 MHz, D_2O) δ 4.46 (d, *J* = 8.0 Hz, 1H), 4.42 (d, *J* = 8.0 Hz, 1H), 4.22 – 3.78 (m, 6H), 3.74 – 3.42 (m, 8H), 3.29 – 3.16 (m, 4H), 2.97 – 2.89 (m, 2H), 1.61 (h, *J* = 7.0, 6.3 Hz, 4H), 1.44 – 1.34 (m, 2H); ^{13}C NMR (101 MHz, D_2O) δ 104.82, 103.93, 81.37, 77.25, 76.66, 76.40, 75.03, 74.84, 72.09, 71.00, 64.05, 62.02, 41.32, 28.42, 24.09; ^{31}P NMR (162 MHz, D_2O) δ 0.26; m/z (HRMS+) 551.2217 $[M+H]^+$ ($C_{19}H_{39}N_2O_{14}P$ requires 551.2217).

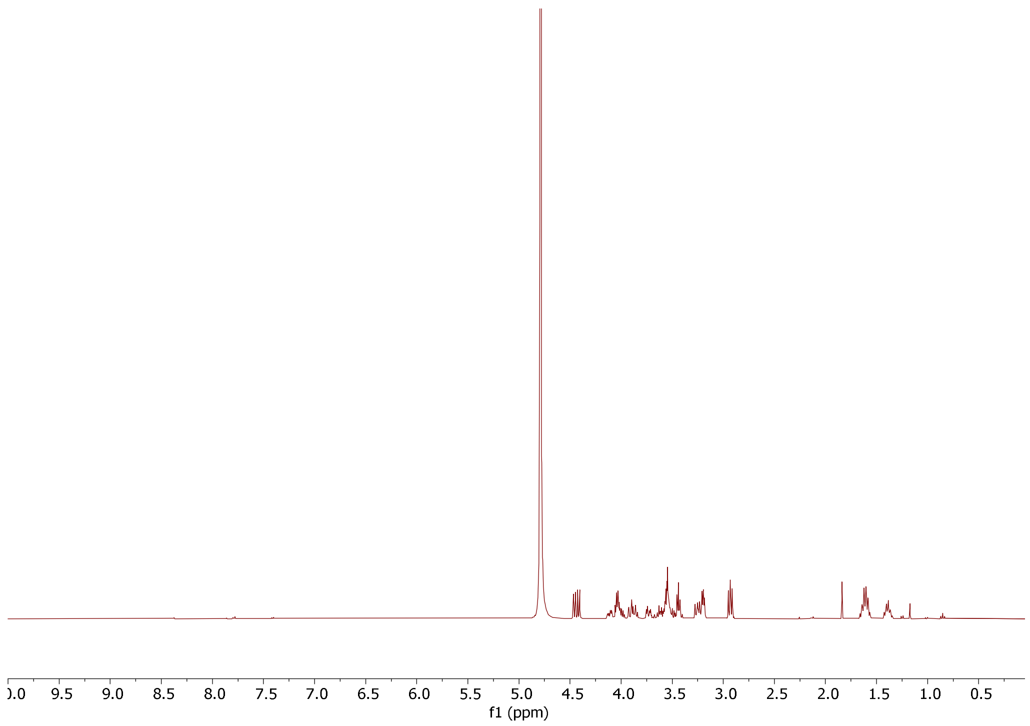

Supplementary Figure 7. ^{1}**H NMR of PA (400 MHz,** D_2O**)**

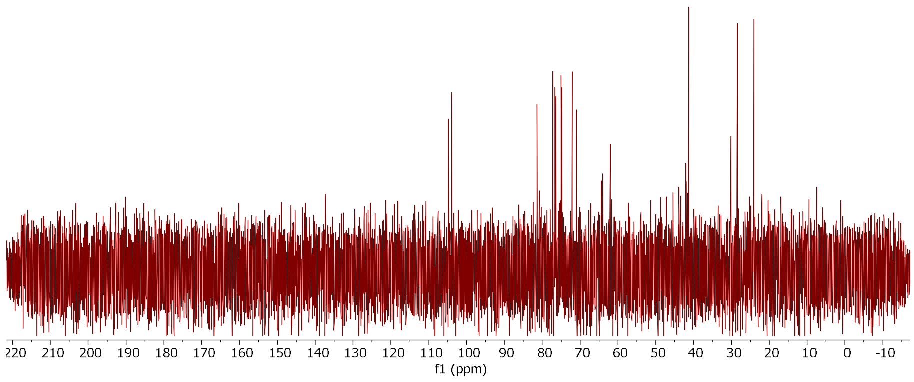

Supplementary Figure 8. ^{13}**C NMR of PA (101 MHz,** D_2O**)**

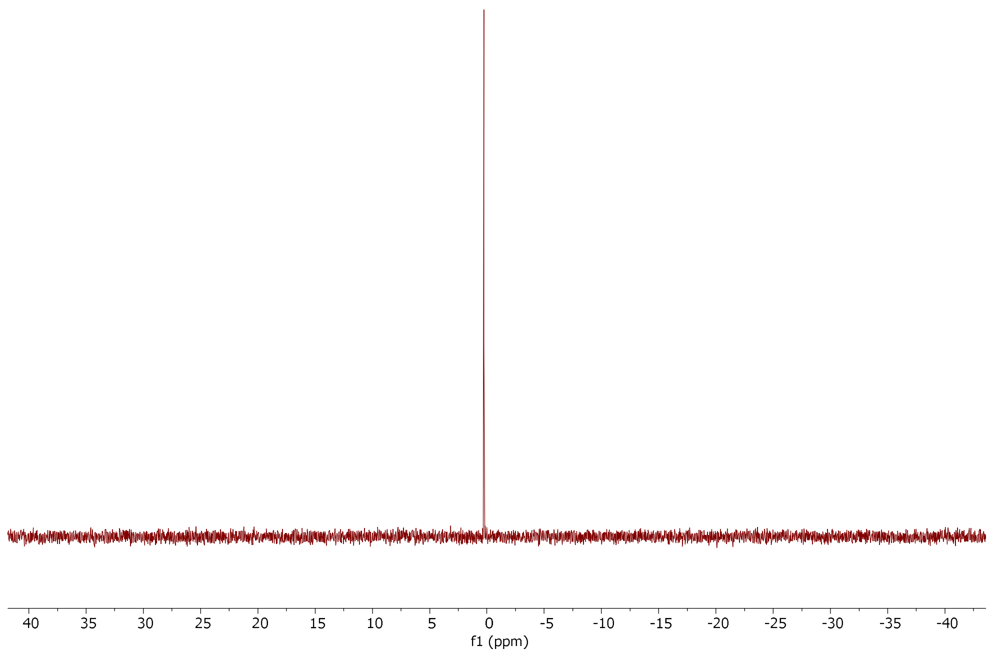

Supplementary Figure 9. ^{31}P NMR of PA (162 MHz, D_2O)

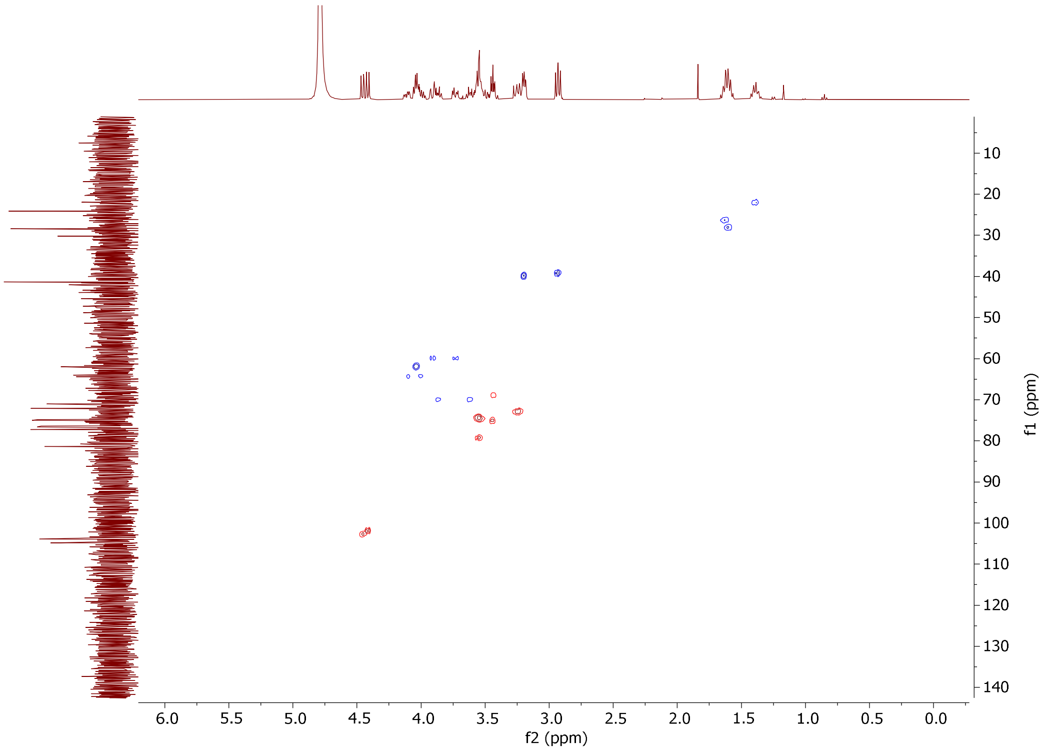

Supplementary Figure 10. HSQC NMR of PA (D_2O)

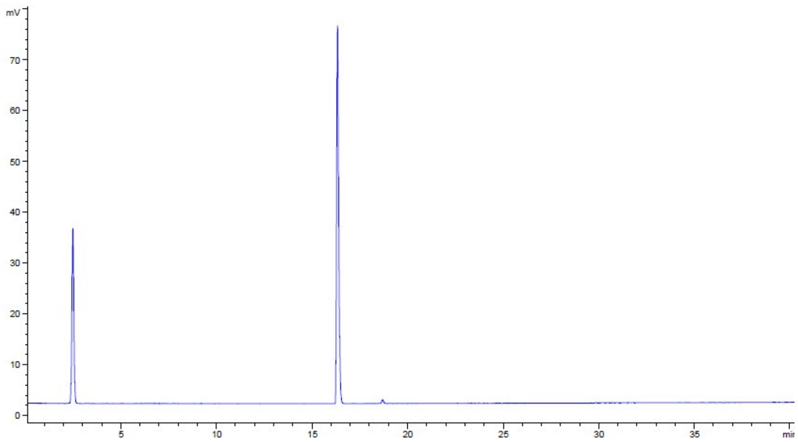

Supplementary Figure 11. RP-HPLC of PA (ELSD trace, Method F, t_R= 16.3 min)

2.7. Synthesis of PAP

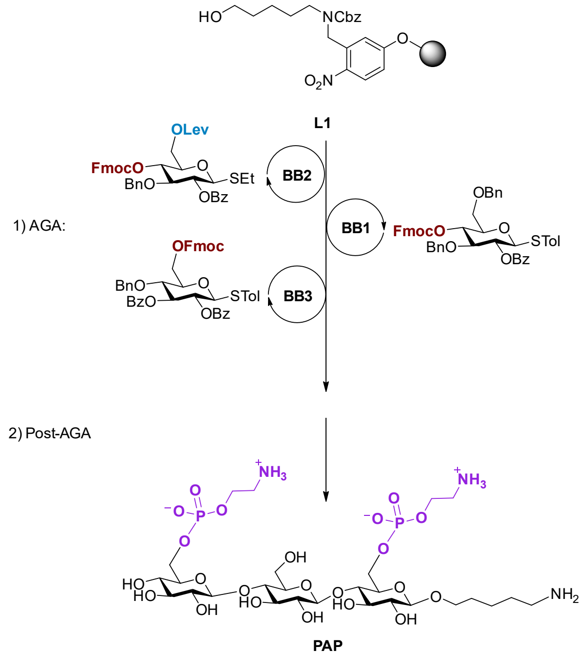

Supplementary Table 8. Synthesis of **PAP**.

Step		Modules	Notes
AGA		**A**	
	**BB2**	**B, C, D, E1**	**C:** (**BB2**, -20 °C for 5 min, 0 °C for 20 min)
	**BB1**	**B, C, D, E1**	**C:** (**BB1**, -20 °C for 5 min, 0 °C for 20 min)
	**BB3**	**B, C, D, E1, E2**	**C:** (**BB3**, -20 °C for 5 min, 0 °C for 20 min)
Post-AGA		**F, J**	**J:** (Method **A_2**, t_R = 22.2 min)
		**G, J1, J2,**	**G:** (4 equiv. of **4** per -OH) **J1:** (Method **B**) **J2:** (Method **C**)
		**H,J1, I, J2**	**J1:** (Method **C**) **I:** 10% Pd/C (12 h) **J2:** (Method **E** and **D**)

Compound **PAP** was obtained as a white solid (2.7 mg, 24 % overall yield).

Analytical data for **PAP:** ^{1}H NMR (600 MHz, D_2O) δ 4.61 (d, *J* = 8.0 Hz, 1H), 4.55 (d, *J* = 8.0 Hz, 1H), 4.52 (d, *J* = 8.0 Hz, 1H), 4.29 – 3.49 (m, 22H), 3.40 – 3.14 (m, 8H), 3.06 – 3.01 (m, 2H), 1.76 – 1.65 (m, 4H), 1.48 (ddd, *J* = 10.8, 8.7, 4.9 Hz, 2H); ^{13}C NMR (151 MHz, D_2O) δ 102.78, 102.12, 102.04, 79.11, 78.00, 75.19, 74.65, 74.54, 74.48, 74.17, 74.13, 73.33, 72.97, 72.88, 72.81, 70.15, 68.98, 61.89, 61.86, 59.85, 52.23,

46.62, 39.99, 39.93, 39.30, 28.09, 26.30, 21.96; ^{31}P NMR (243 MHz, D_2O) δ 0.30, -0.04; m/z (HRMS+) 836.2854 $[M+H]^+$ ($C_{27}H_{56}N_3O_{22}P_2$ requires 836.2831).

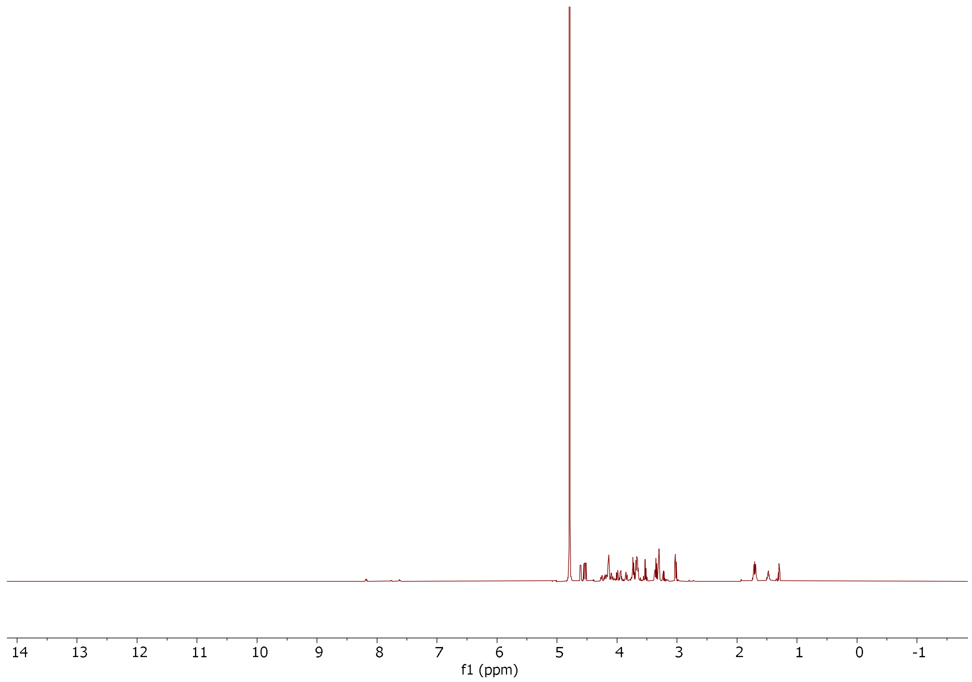

Supplementary Figure 12. ^{1}H NMR of PAP (600 MHz, D_2O)

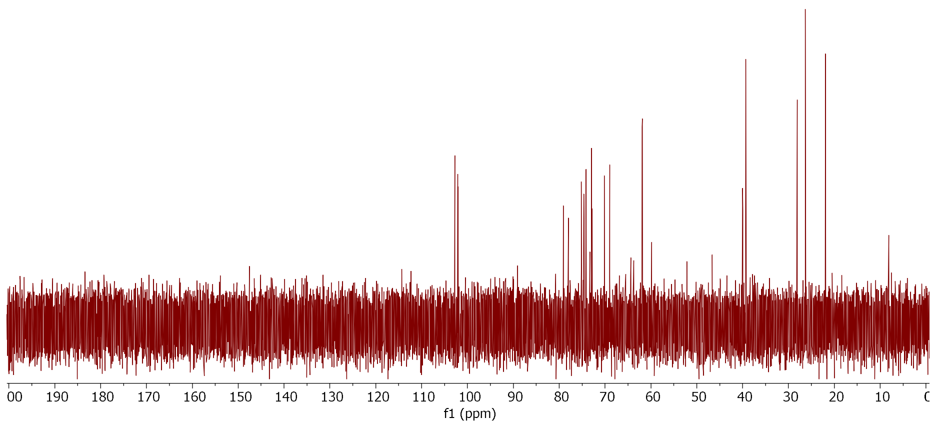

Supplementary Figure 13. ^{13}C NMR of PAP (151 MHz, D_2O)

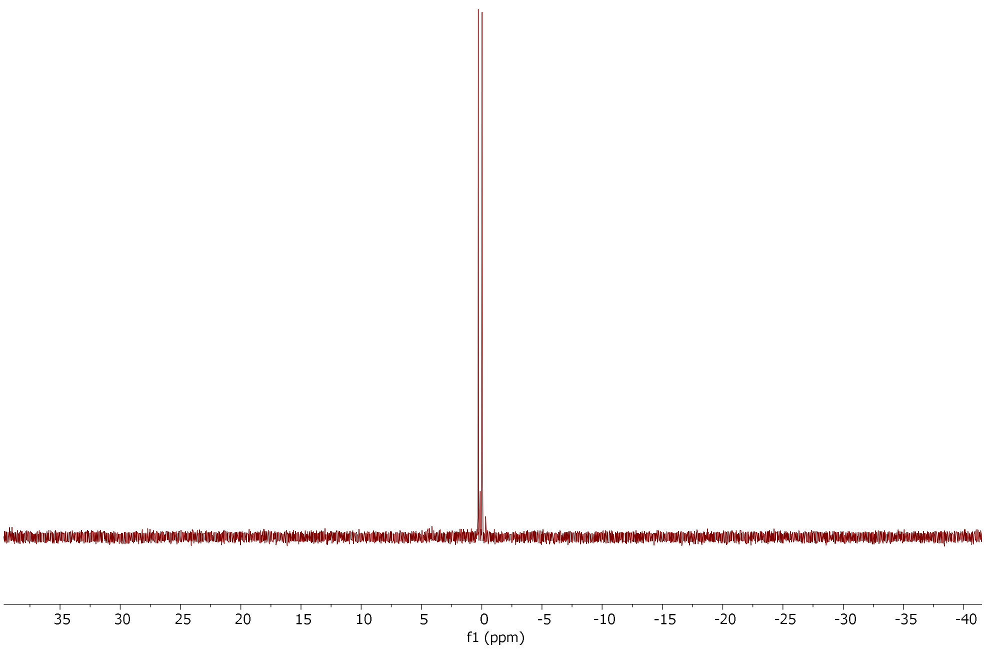

Supplementary Figure 14. ^{31}P NMR of PAP (243 MHz, D_2O)

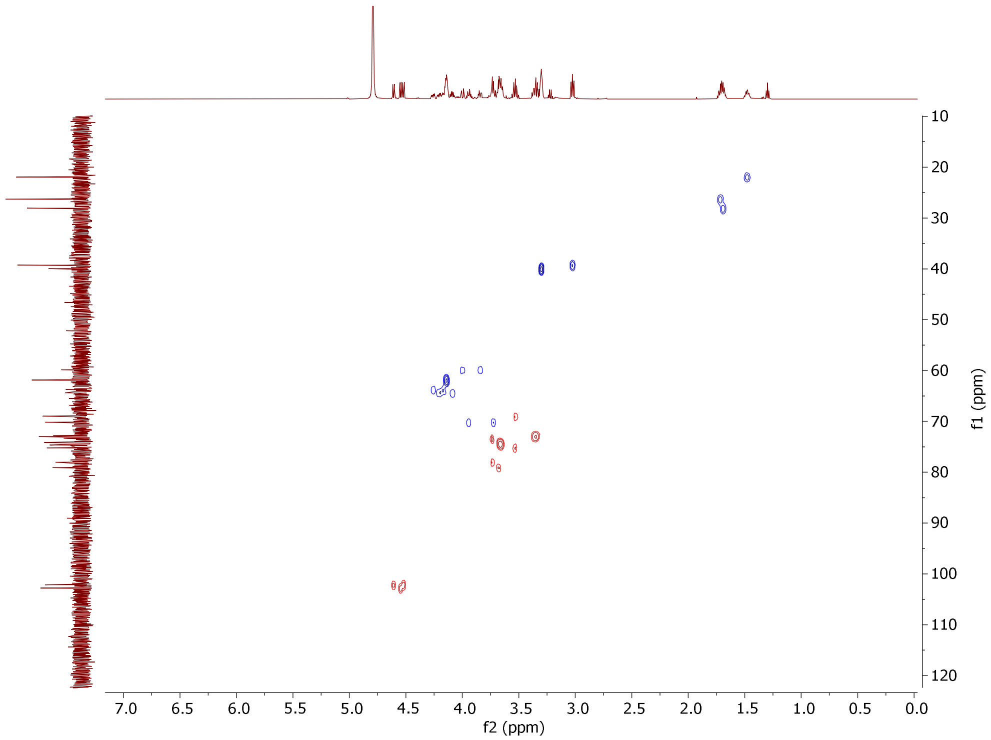

Supplementary Figure 15. HSQC NMR of PAP (D_2O)

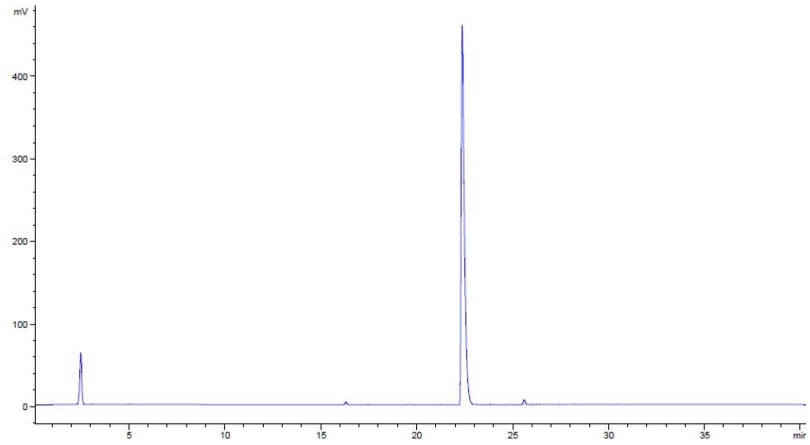

Supplementary Figure 16. RP-HPLC of PAP (ELSD trace, Method F, t_R= 22.2 min)

2.8. Synthesis of PA_5

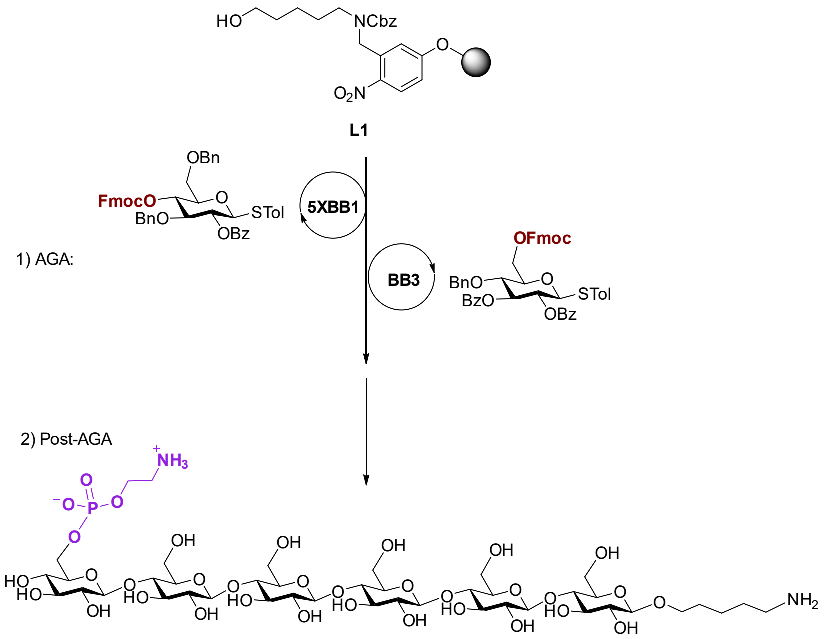

PA_5

Supplementary Table 9. Synthesis of **PA_5**.

Step		Modules	Notes
AGA		**A**	
	**5xBB1**	**B, C, D, E1**	**C:** (**BB1**, -20 °C for 5 min, 0 °C for 20 min)
	**BB3**	**B, C, D, E1**	**C:** (**BB3**, -20 °C for 5 min, 0 °C for 20 min)
Post-AGA		**F, J**	**J:** (Method **A_2**, t_R = 37.2 min)
		**G, J1, J2,**	**G:** (4 equiv. of **4** per -OH) **J1:** (Method **B**) **J2:** (Method **C**)
		**H,J1, I, J2**	**J1:** (Method **C**) **I:** 10% Pd/C (48 h) **J2:** (Method **E** and **D**)

Compound **PA$_5$** was obtained as a white solid (1.3 mg, 16% overall yield).

Analytical data for **PA$_5$:** ^{1}H NMR (600 MHz, D_2O) δ 4.55 (dd, *J* = 7.9, 2.3 Hz, 5H), 4.50 (d, *J* = 8.0 Hz, 1H), 4.20 (ddd, *J* = 11.5, 5.3, 2.1 Hz, 1H), 4.16 – 4.11 (m, 2H), 4.09 (dt, *J* = 11.1, 5.4 Hz, 1H), 4.03 – 3.93 (m, 6H), 3.83 (m, 5H), 3.73 – 3.62 (m, 16H), 3.53 (dd, *J* = 8.6, 6.9 Hz, 2H), 3.41 – 3.28 (m, 8H), 3.05 – 3.00 (m, 2H), 1.76 – 1.66 (m, 4H), 1.51 – 1.43 (m, 2H); ^{13}C NMR (151 MHz, D_2O) δ 102.73, 102.28, 102.20, 101.94, 79.02, 78.51, 78.20, 75.20, 74.75, 74.71, 74.65, 74.49, 74.28, 74.01, 73.93, 72.98, 72.86, 72.81, 70.02, 68.97, 61.89, 59.78, 39.99, 39.29, 28.09, 26.34, 22.01; ^{31}P NMR (243 MHz, D_2O) δ 0.30; m/z (HRMS+) 600.2223 $[M+2H]^{2+}$ ($C_{43}H_{81}N_2O_{34}P$ requires 600.2198).

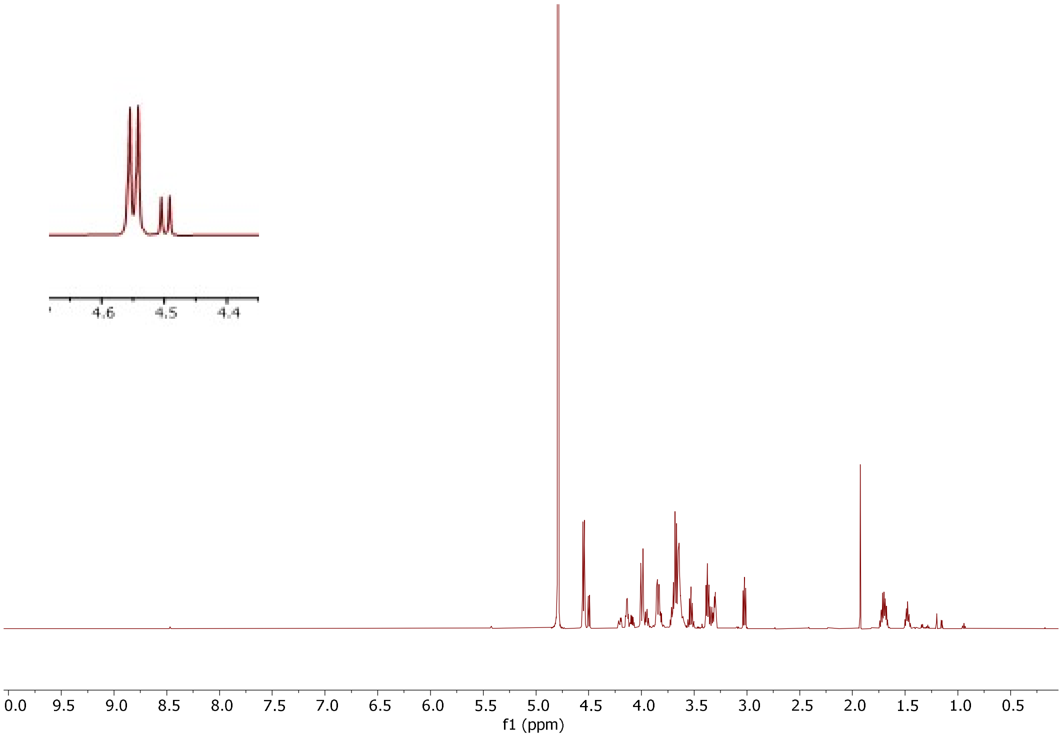

Supplementary Figure 17. ^{1}H NMR of PA$_5$ (600 MHz, D_2O)

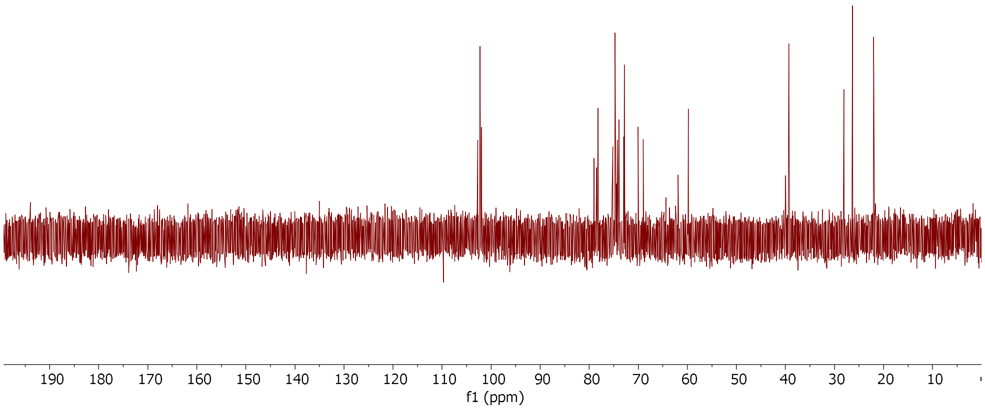

Supplementary Figure 18. ^{13}C NMR of PA_5 (151 MHz, D_2O)

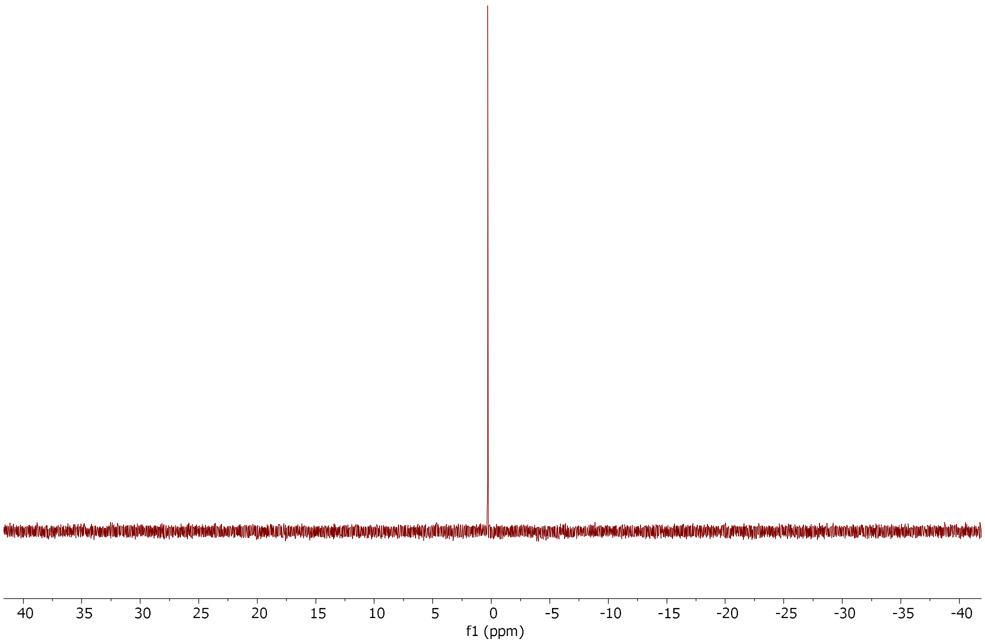

Supplementary Figure 19. ^{31}P NMR of PA_5 (243 MHz, D_2O)

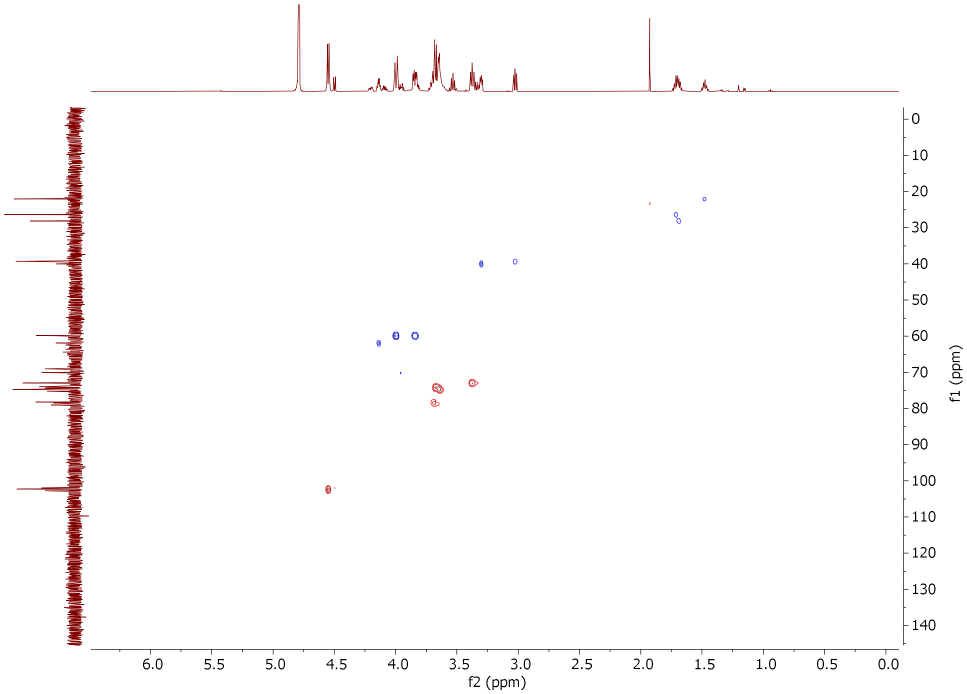

Supplementary Figure 20. HSQC NMR of PA_5 (D_2O)

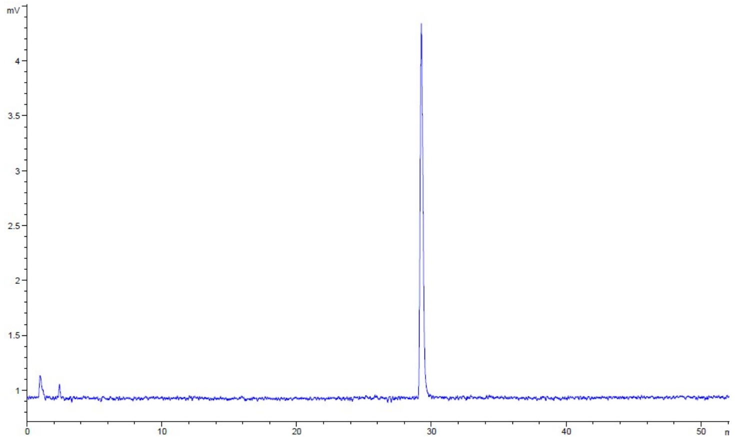

Supplementary Figure 21. RP-HPLC of PA_5 (ELSD trace, Method F, t_R= 29.2 min)

2.9. Synthesis of A_3PA_2

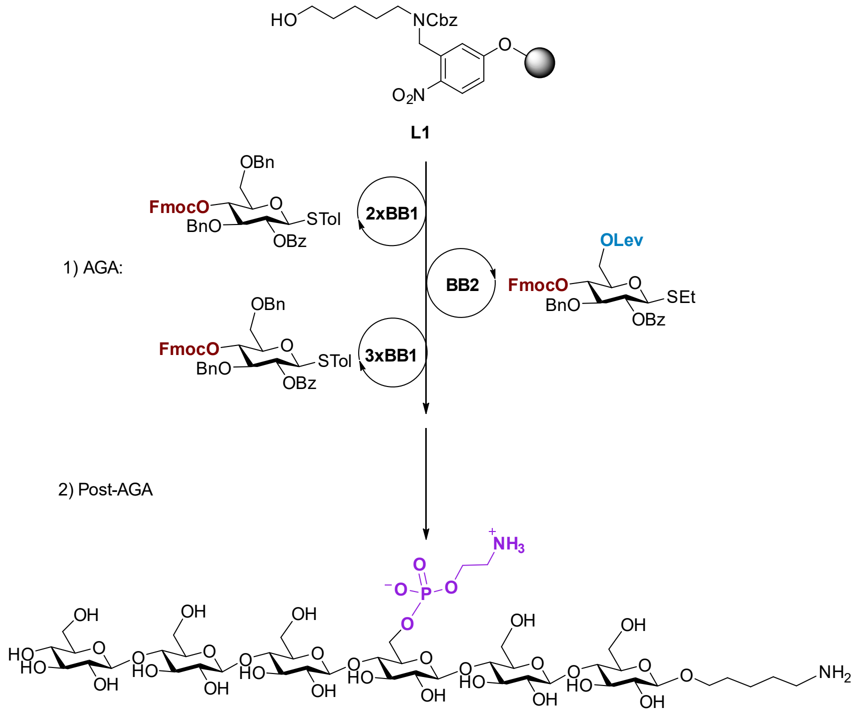

A_3PA_2

Supplementary Table 10. Synthesis of $\mathbf{A_3PA_2}$.

Step		Modules	Notes
AGA		**A**	
	**2xBB1**	**B, C, D, E1**	**C:** (**BB1**, -20 °C for 5 min, 0 °C for 20 min)
	**BB2**	**B, C, D, E1**	**C:** (**BB2**, -20 °C for 5 min, 0 °C for 20 min)
	**3xBB1**	**B, C, D, E1, D, E2**	**C:** (**BB1**, -20 °C for 5 min, 0 °C for 20 min)
Post-AGA		**F, J**	**J:** (Method $\mathbf{A_2}$, t_R = 36.9 min)
		**G, J1, J2,**	**G:** (4 equiv. of **4** per -OH)<br>**J1:** (Method **B**)<br>**J2:** (Method **C**)
		**H,J1, I, J2**	**J1:** (Method **C**) **I:** 10% Pd/C (48 h) **J2:** (Method **E** and **D**)

Compound **A_3PA_2** was obtained as a white solid (0.8 mg, 10% overall yield).

Analytical data for **A_3PA_2:** ^{1}H NMR (700 MHz, D_2O) δ 4.63 – 4.47 (m, 6H), 4.27 (dd, *J* = 10.8, 5.0 Hz, 1H), 4.21 – 4.08 (m, 3H), 4.03 – 3.89 (m, 6H), 3.87 – 3.58 (m, 21H), 3.56 – 3.26 (m, 11H), 3.06 – 2.98 (m, 2H), 1.93 (s, 2H), 1.72 – 1.64 (m, 4H), 1.53 – 1.43 (m, 2H)*;* ^{13}C NMR (176 MHz, D_2O) δ 102.49, 102.28, 101.95, 75.91, 74.75, 74.27, 73.97, 73.80, 72.85, 70.02, 69.37, 59.82, 39.28, 28.08, 26.34, 22.00*;* ^{31}P NMR (243 MHz, D_2O) δ -0.01; m/z (HRMS+) 600.2222 $[M+2H]^{2+}$ ($C_{43}H_{81}N_2O_{34}P$ requires 600.2198).

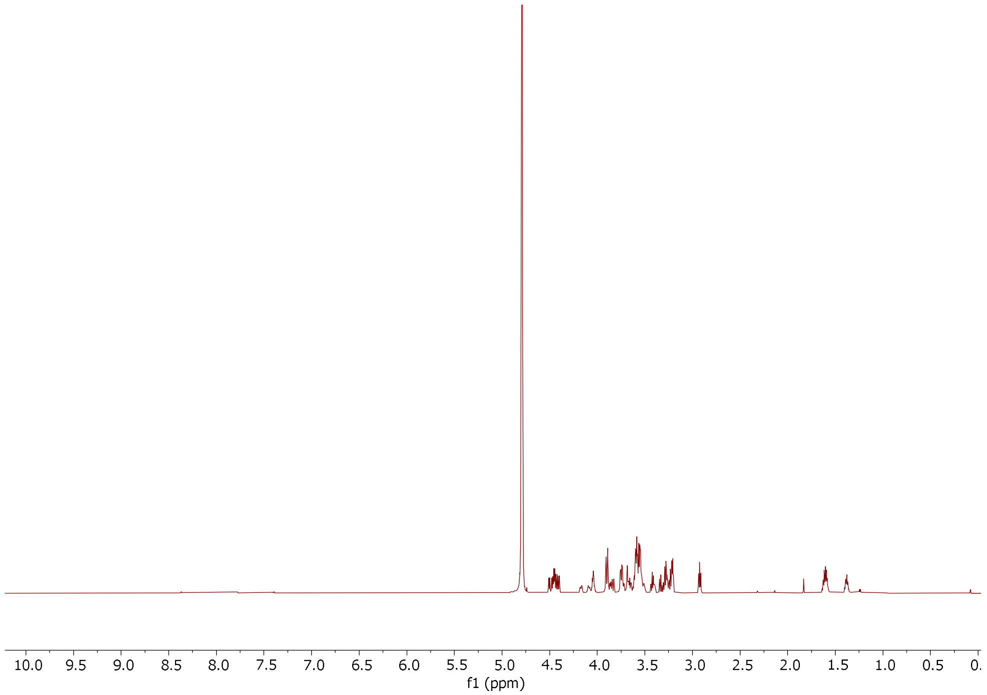

Supplementary Figure 22. ^{1}H NMR of A_3PA_2 (700 MHz, D_2O)

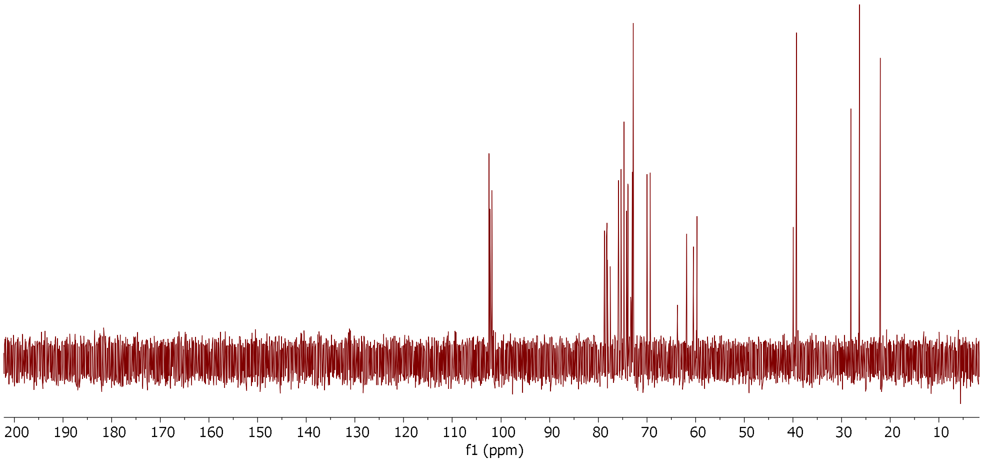

Supplementary Figure 23. ^{13}C NMR of A_3PA_2 (176 MHz, D_2O)

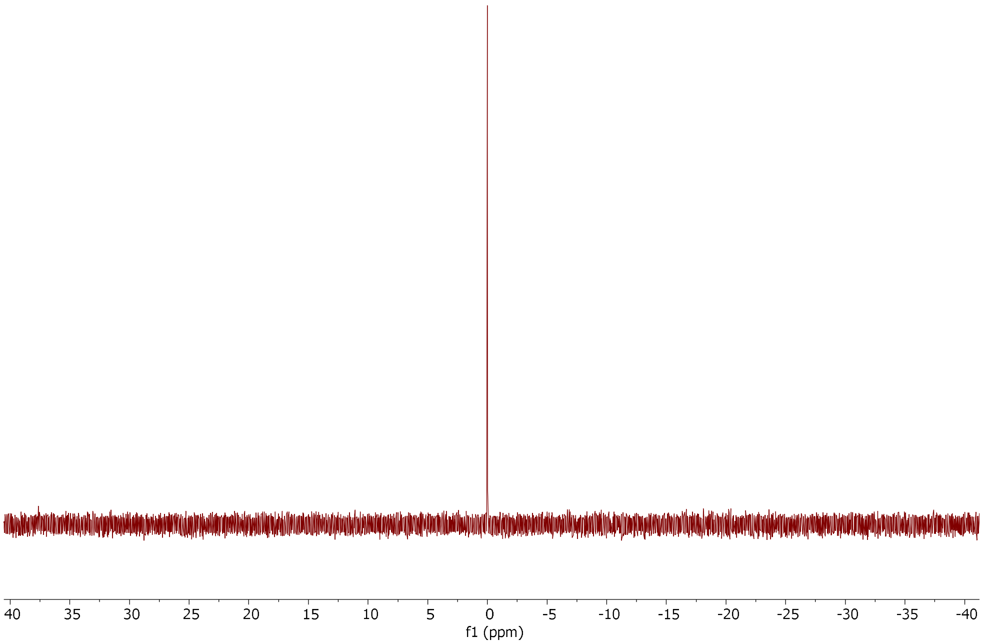

Supplementary Figure 24. ^{31}P NMR of A_3PA_2 (243 MHz, D_2O)

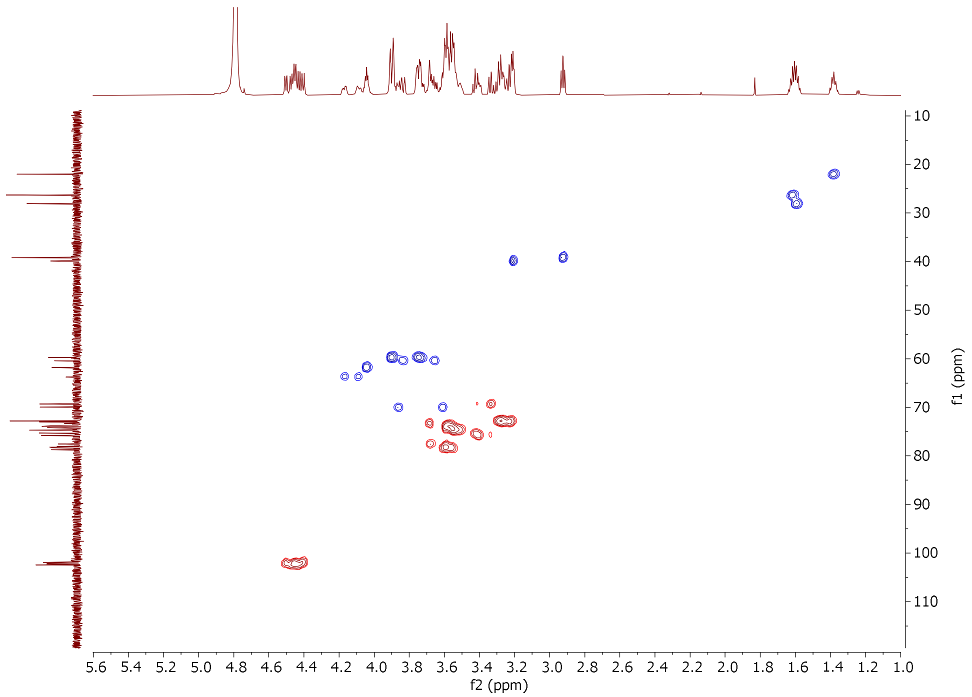

Supplementary Figure 25. HSQC NMR of A_3PA_2 (D_2O)

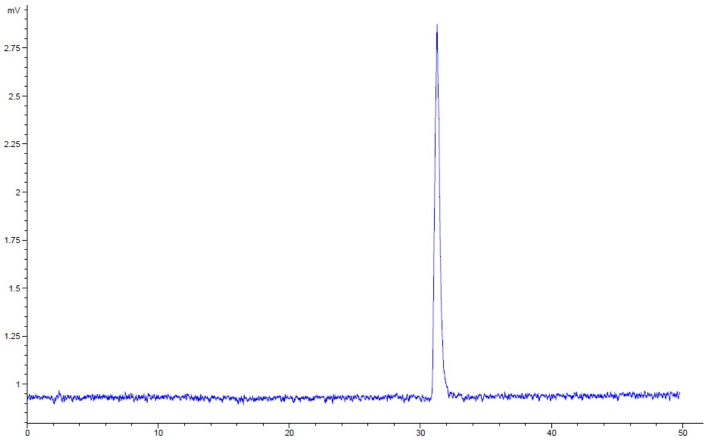

Supplementary Figure 26. RP-HPLC of A_3PA_2 (ELSD trace, Method F, t_R= 31.2 min)

2.10. Synthesis of (APA)$_2$

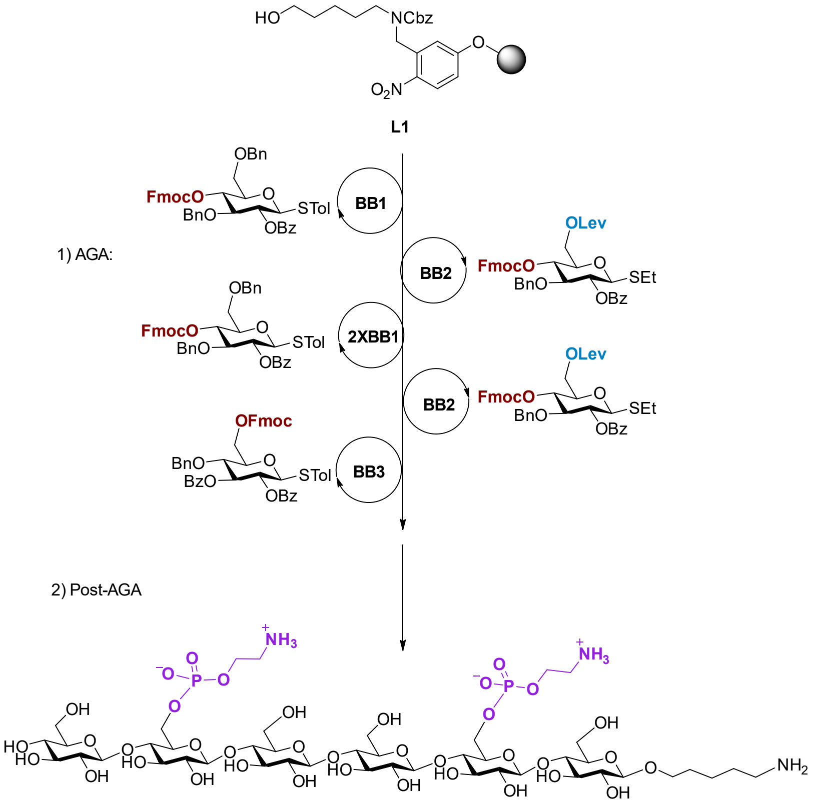

(APA)$_2$

Supplementary Table 11. Synthesis of **(APA)$_2$**.

Step		Modules	Notes
AGA		**A**	
	**BB1**	**B, C, D, E1**	**C:** (**BB1**, -20 °C for 5 min, 0 °C for 20 min)
	**BB2**	**B, C, D, E1**	**C:** (**BB2**, -20 °C for 5 min, 0 °C for 20 min)
	**2xBB1**	**B, C, D, E1**	**C:** (**BB1**, -20 °C for 5 min, 0 °C for 20 min)
	**BB2**	**B, C, D, E1**	**C:** (**BB2**, -20 °C for 5 min, 0 °C for 20 min)
	**BB3**	**B, C, D, E1, D, E2**	**C:** (**BB3**, -20 °C for 5 min, 0 °C for 20 min)
Post-AGA		**F, J**	**J:** (Method **A$_2$**, t_R = 46.1 min)
		**G, J1, J2,**	**G:** (5 equiv. of **4** per -OH)<br>**J1:** (Method **B**)<br>**J2:** (Method **C**)
		**H,J1, I, J2**	**J1:** (Method **C**) **I:** 10% Pd/C (48 h) **J2:** (Method **E** and **D**)

Compound **(APA)₂** was obtained as a white solid (2 mg, 11% overall yield).

Analytical data for **$(APA)_2$:** ^{1}H NMR (600 MHz, D_2O) δ 4.64 – 4.46 (m, 6H), 4.31 – 3.27 (m, 46H), 3.04 – 3.00 (m, 2H), 1.70 (dp, *J* = 13.9, 7.2 Hz, 4H), 1.48 (q, *J* = 8.0 Hz, 2H); ^{13}C NMR (151 MHz, D_2O) δ 102.51, 102.49, 102.37, 102.23, 102.16, 101.91, 79.16, 78.84, 78.17, 77.80, 75.91, 75.46, 74.75, 74.67, 74.62, 74.32, 73.96, 73.83, 73.34, 73.17, 72.94, 72.83, 72.79, 70.03, 69.43, 61.84, 60.50, 59.97, 59.81, 40.01, 39.96, 39.28, 28.09, 26.33, 22.01; ^{31}P NMR (243 MHz, D_2O) δ -0.02, -0.05; m/z (HRMS+) 661.7258 $[M+2H]^{+2}$ ($C_{45}H_{87}N3O_{37}P_2$ requires 661.7241).

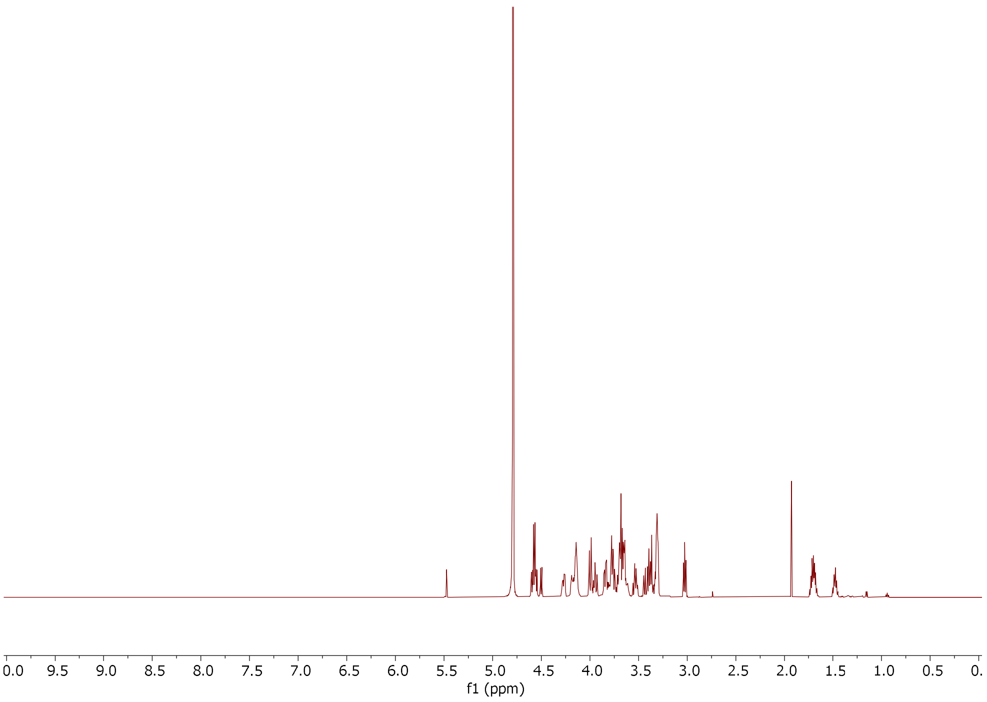

Supplementary Figure 27. ^{1}H NMR of $(APA)_2$ (600 MHz, D_2O)

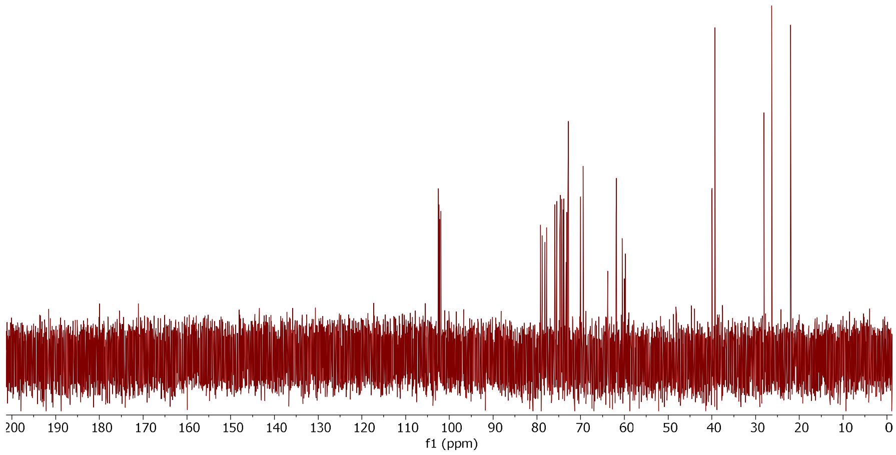

Supplementary Figure 28. ^{13}C NMR of $(APA)_2$ (151 MHz, D_2O)

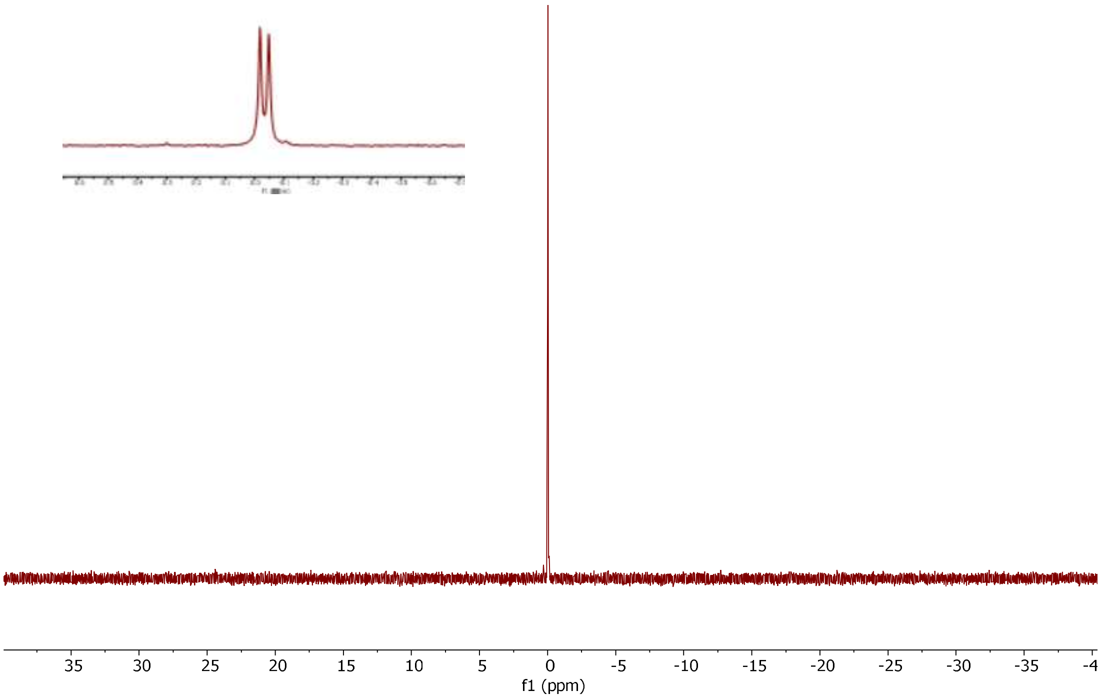

Supplementary Figure 29. ^{31}P NMR of $(APA)_2$ (243 MHz, D_2O)

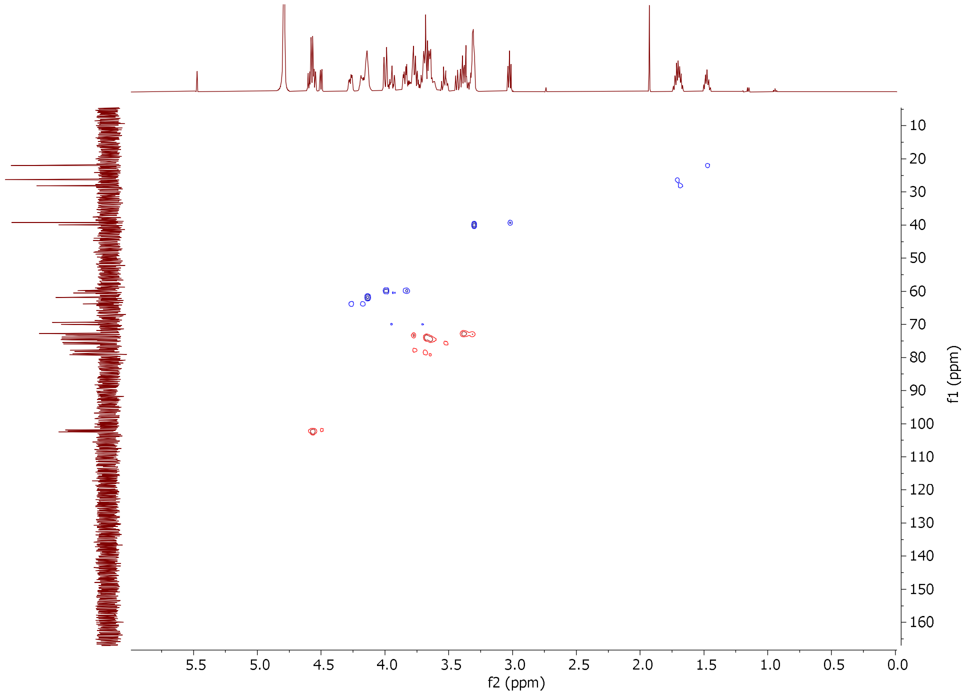

Supplementary Figure 30. HSQC NMR of $(APA)_2$ (D_2O)

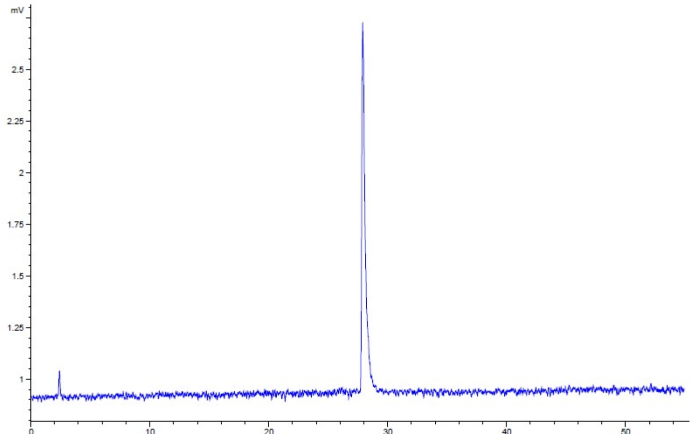

Supplementary Figure 31. RP-HPLC of $(APA)_2$ (ELSD trace, Method F, t_R= 27.8 min)

2.11. Synthesis of $A_2P_2A_2$

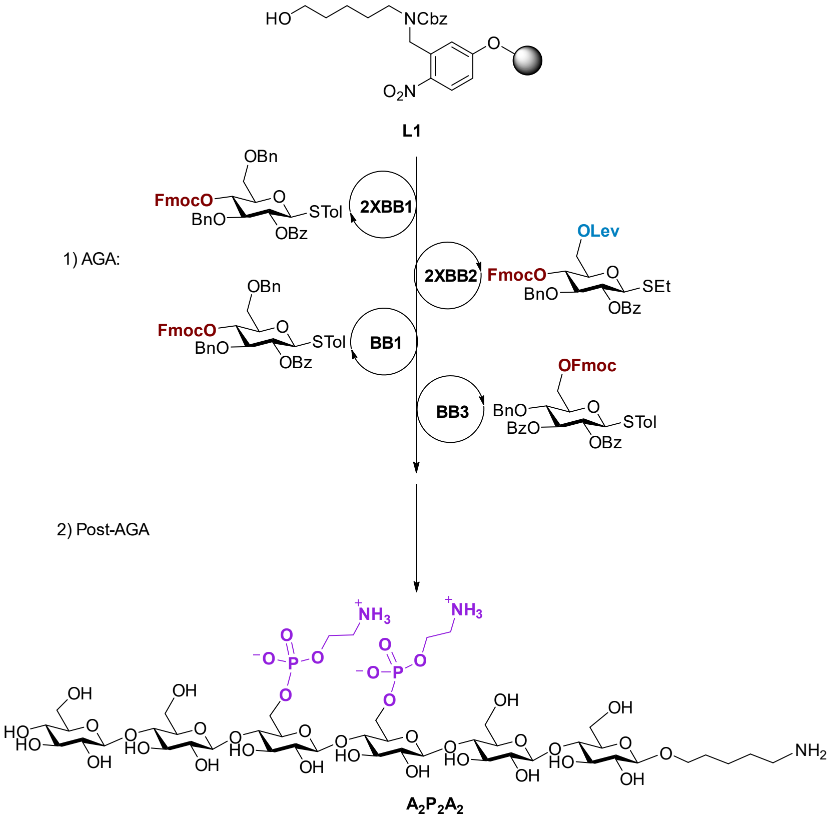

Supplementary Table 12. Synthesis of $\mathbf{A_2P_2A_2}$.

Step		Modules	Notes
AGA		**A**	
	**2xBB1**	**B, C, D, E1**	**C:** (**BB1**, -20 °C for 5 min, 0 °C for 20 min)
	**2XBB2**	**B, C, D, E1**	**C:** (**BB2**, -20 °C for 5 min, 0 °C for 20 min)
	**BB1**	**B, C, D, E1**	**C:** (**BB1**, -20 °C for 5 min, 0 °C for 20 min)
	**BB3**	**B, C, D, E1, D, E2**	**C:** (**BB3**, -20 °C for 5 min, 0 °C for 20 min)
Post-AGA		**F, J**	**J:** (Method $\mathbf{A_2}$, t_R = 41.2 min)
		**G, J1, J2,**	**G:** (5 equiv. of **4** per -OH)<br>**J1:** (Method **B**)<br>**J2:** (Method **C**)
		**H,J1, I, J2**	**J1:** (Method **C**) **I:** 10% Pd/C (48 h) **J2:** (Method **E** and **D**)

Compound **$A_2P_2A_2$** was obtained as a white solid (1.7 mg, 9 % overall yield).

Analytical data for **$A_2P_2A_2$:** ^{1}H NMR (600 MHz, D_2O) δ 4.61 (t, *J* = 8.4 Hz, 2H), 4.57 (d, *J* = 7.9 Hz, 1H), 4.55 (d, *J* = 7.9 Hz, 1H), 4.52 (d, *J* = 7.9 Hz, 1H), 4.50 (d, *J* = 8.0 Hz, 1H), 4.27 (dd, *J* = 11.0, 4.9 Hz, 2H), 4.22 – 4.07 (m, 6H), 4.03 – 3.90 (m, 5H), 3.86 – 3.59 (m, 19H), 3.55 – 3.47 (m, 2H), 3.45 – 3.27 (m, 11H), 3.05 – 3.00 (m, 2H), 1.74 – 1.66 (m, 4H), 1.51 – 1.44 (m, 2H))*;* ^{13}C NMR (151 MHz, D_2O) δ 102.49, 102.45, 102.24, 102.13, 101.94, 78.79, 78.47, 78.41, 78.33, 77.66, 75.91, 75.39, 74.75, 74.70, 74.68, 74.27, 74.00, 73.98, 73.85, 73.83, 73.33, 73.07, 72.93, 72.87, 72.85, 72.75, 70.03, 69.38, 63.81, 61.90, 61.87, 60.50, 59.93, 59.82, 58.38, 46.62, 40.02, 39.97, 39.29, 28.09, 26.34, 23.18, 22.01, 8.15*;* ^{31}P NMR (243 MHz, D_2O) δ -0.01, -0.05*;* m/z (HRMS+) 661.7283 $[M+2H]^{+2}$ ($C_{45}H_{87}N3O_{37}P_2$ requires 661.7241).

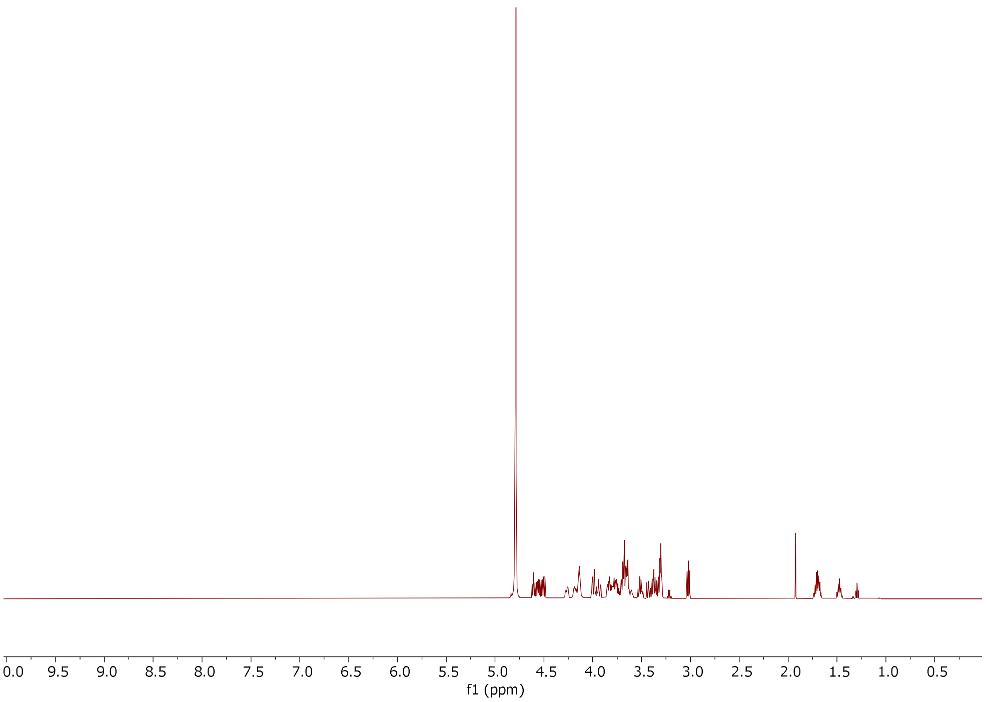

Supplementary Figure 32. ^{1}H NMR of $A_2P_2A_2$ (600 MHz, D_2O)

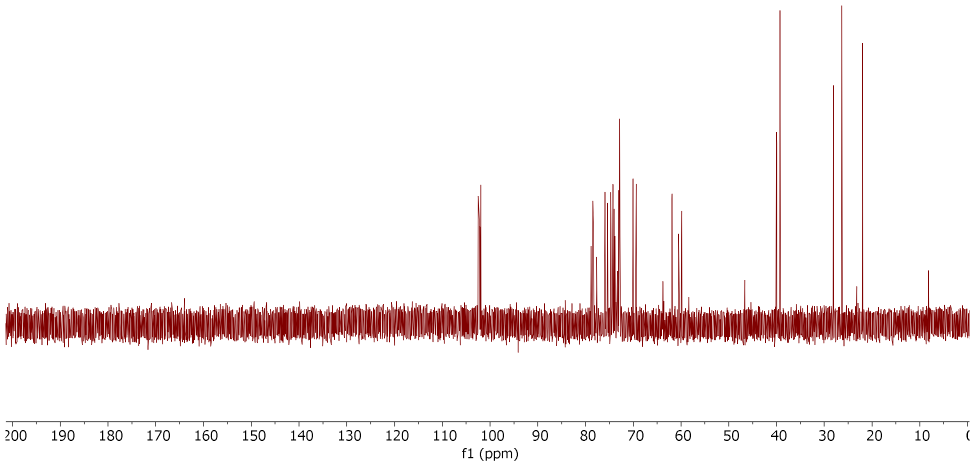

Supplementary Figure 33. ^{13}C **NMR of** $A_2P_2A_2$ **(151 MHz,** D_2O**)**

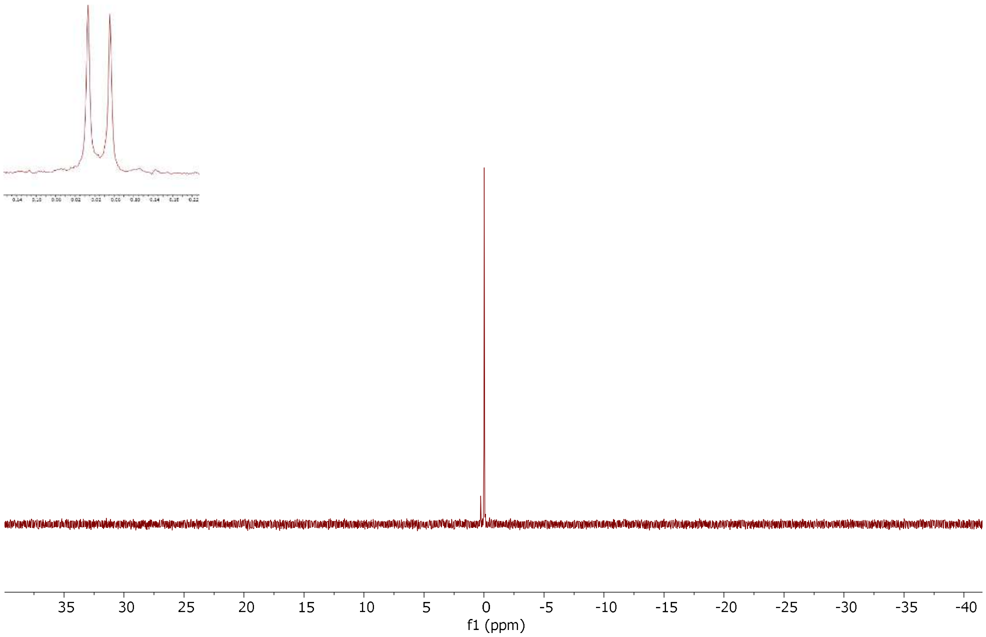

Supplementary Figure 34. ^{31}P **NMR of** $A_2P_2A_2$ **(243 MHz,** D_2O**)**

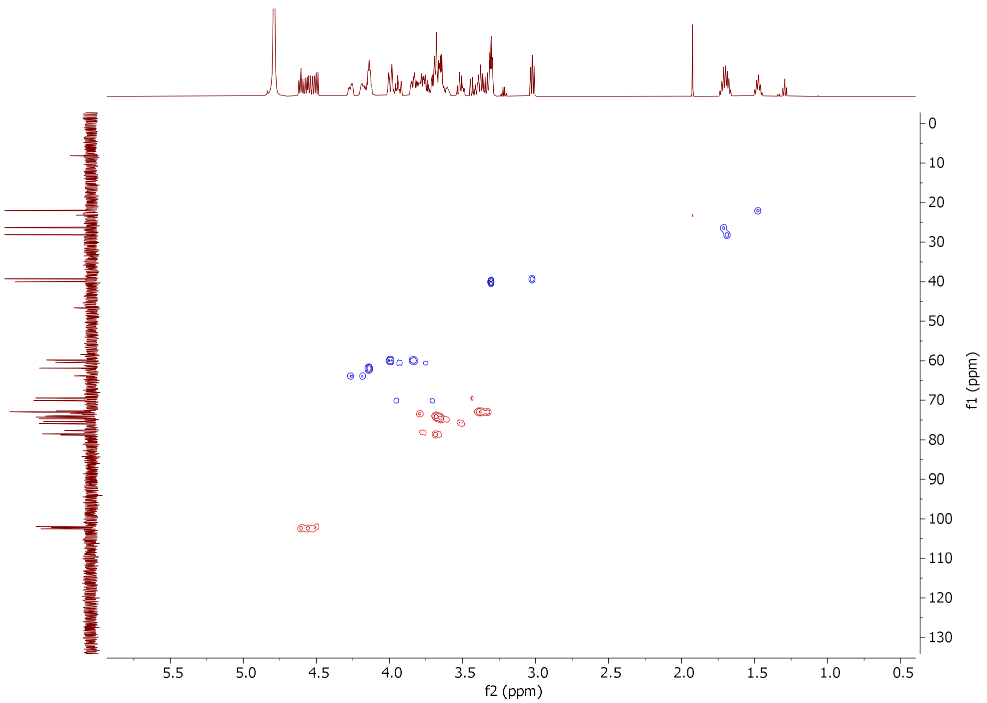

Supplementary Figure 35. HSQC NMR of $A_2P_2A_2$ (D_2O)

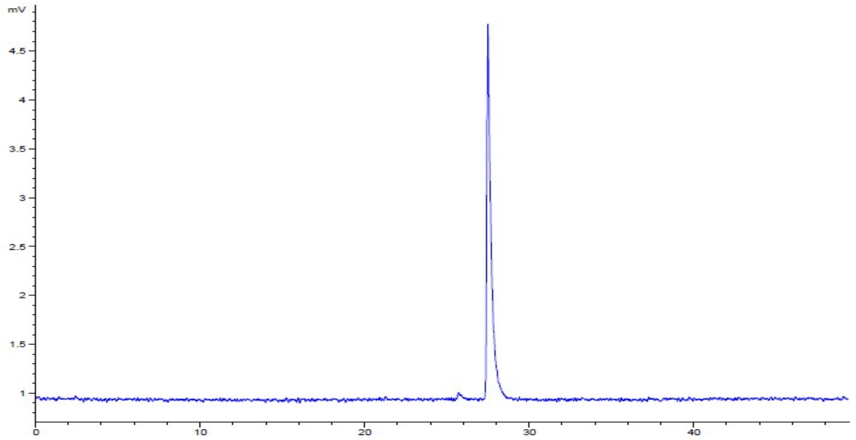

Supplementary Figure 36. RP-HPLC of $A_2P_2A_2$ (ELSD trace, Method F, t_R= 27.4 min)

2.12. Synthesis of P_2APA_2

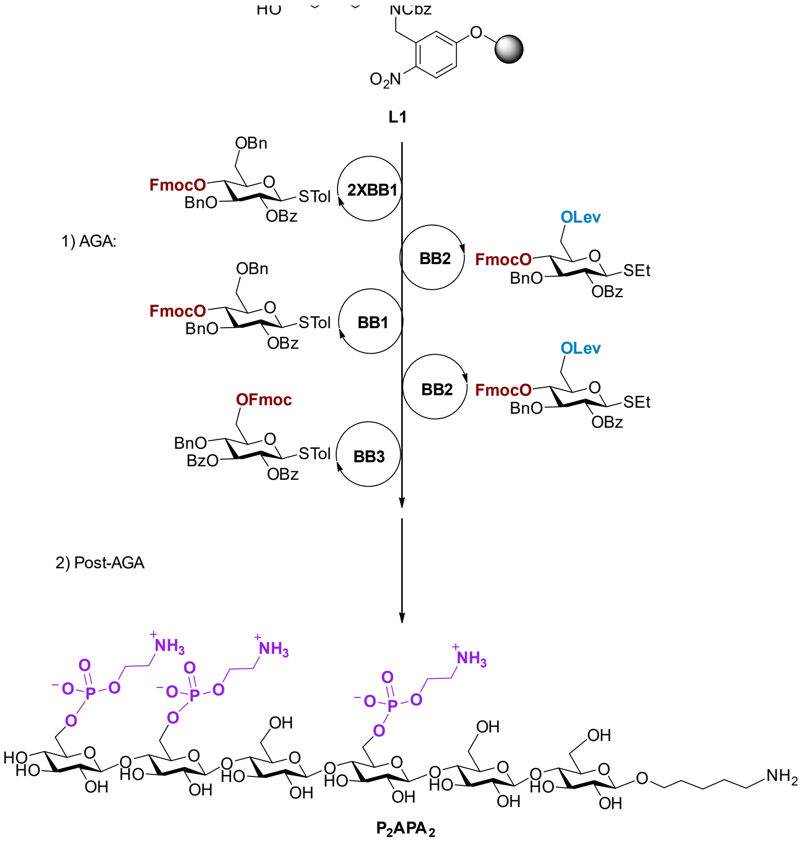

Supplementary Table 13. Synthesis of **P_2APA_2**.

Step		Modules	Notes
AGA		**A**	
	**2xBB1**	**B, C, D, E1**	**C:** (**BB1**, -20 °C for 5 min, 0 °C for 20 min)
	**BB2**	**B, C, D, E1**	**C:** (**BB2**, -20 °C for 5 min, 0 °C for 20 min)
	**BB1**	**B, C, D, E1**	**C:** (**BB1**, -20 °C for 5 min, 0 °C for 20 min)
	**BB2**	**B, C, D, E1**	**C:** (**BB2**, -20 °C for 5 min, 0 °C for 20 min)
	**BB3**	**B, C, D, E1, E2**	**C:** (**BB3**, -20 °C for 5 min, 0 °C for 20 min)
Post-AGA		**F, J**	**J:** (Method **A_2**, t_R = 48.7 min)
		**G, J1, J2,**	**G:** (5 equiv. of **4** per -OH) **J1:** (Method **B**) **J2:** (Method **C**)
		**H,J1, I, J2**	**J1:** (Method **C**) **I:** 10% Pd/C (48 h) **J2:** (Method **E** and **D**)

Compound **P_2APA_2** was obtained as a white solid (1.6 mg, 8 % overall yield).

Analytical data for **P_2APA_2:** ^{1}H NMR (600 MHz, d_2o) δ 4.58 (ddd, *J* = 20.7, 10.4, 6.8 Hz, 5H), 4.50 (d, *J* = 7.9 Hz, 1H), 4.32 – 4.05 (m, 11H), 4.03 – 3.90 (m, 4H), 3.89 – 3.48 (m, 22H), 3.47 – 3.23 (m, 11H), 3.02 (t, *J* = 7.6 Hz, 2H), 1.70 (dp, *J* = 14.1, 7.2 Hz, 4H), 1.47 (p, *J* = 7.8 Hz, 2H)*;* ^{13}C NMR (151 MHz, D_2O) δ 102.49, 102.45, 102.24, 102.13, 101.94, 78.79, 78.47, 78.41, 78.33, 77.66, 75.91, 75.39, 74.75, 74.70, 74.68, 74.27, 74.00, 73.98, 73.85, 73.83, 73.33, 73.07, 72.93, 72.87, 72.85, 72.75, 70.03, 69.38, 63.81, 61.90, 61.87, 60.50, 59.93, 59.82, 58.38, 46.62, 40.02, 39.97, 39.29, 28.09, 26.34, 23.18, 22.01, 8.15*;* ^{31}P NMR (243 MHz, D_2O) δ 0.31, 0.04, -0.02*;* m/z (HRMS+) 482.4858 $[M+3H]^{+3}$ ($C_{47}H_{94}N_4O_{40}P_3$ requires 482.4880).

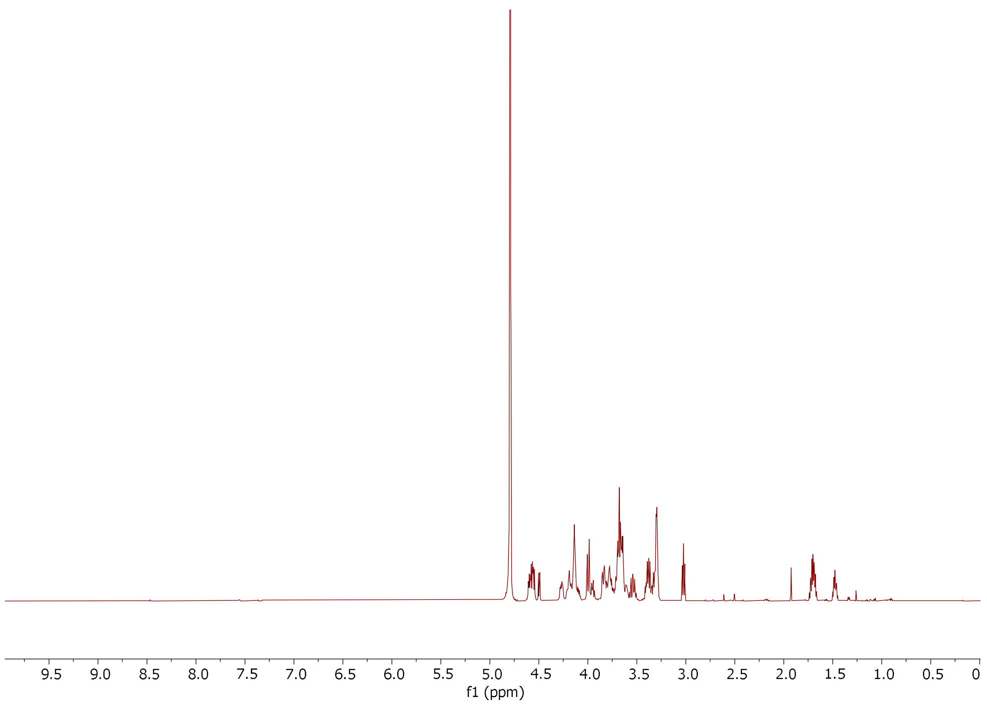

Supplementary Figure 37. ^{1}H NMR of P_2APA_2 (600 MHz, D_2O)

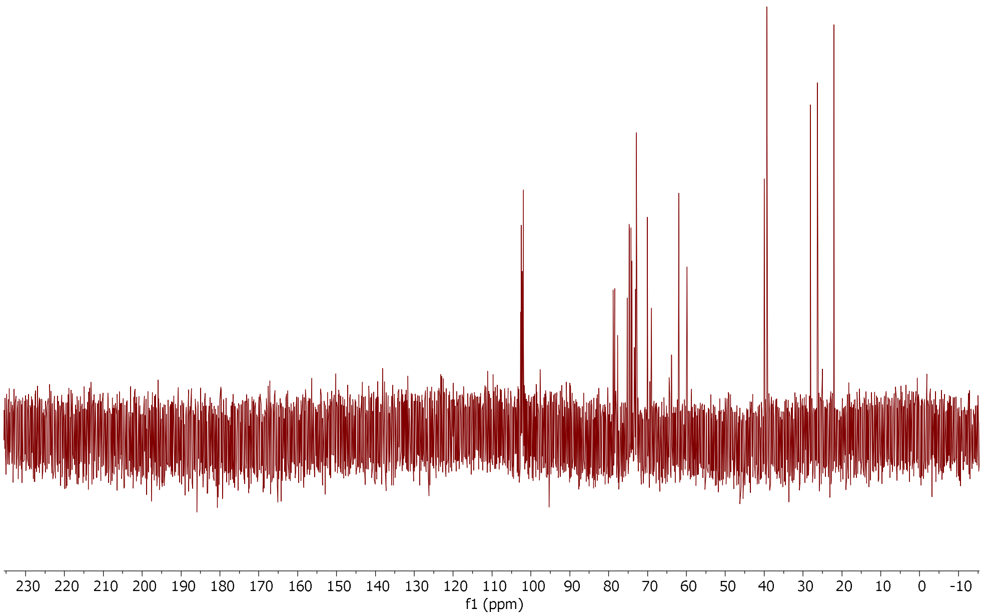

Supplementary Figure 38. ^{13}C NMR of P_2APA_2 (151 MHz, D_2O)

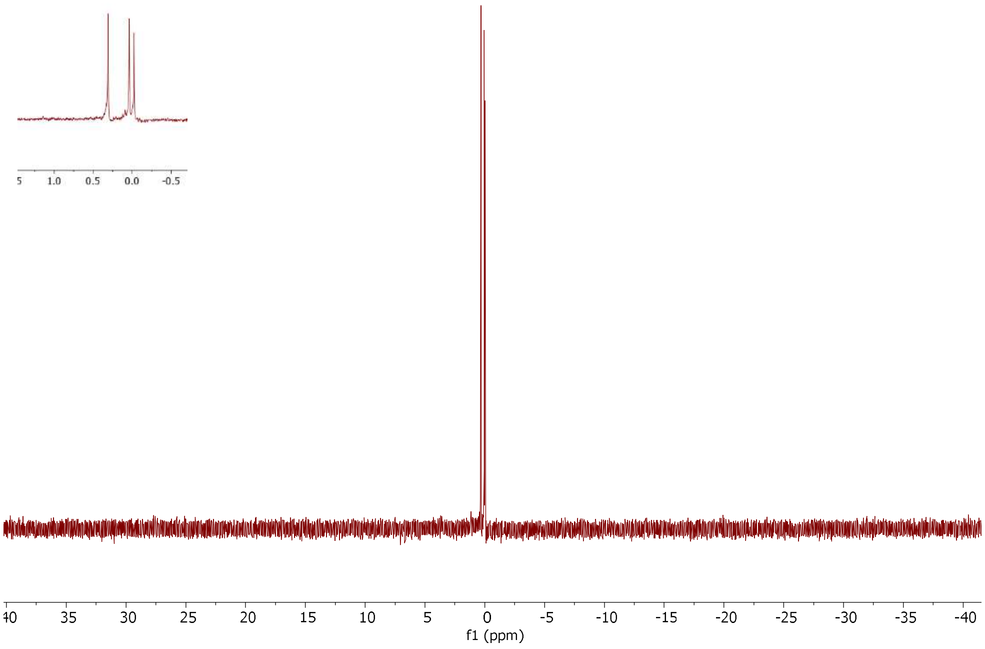

Supplementary Figure 39. ^{31}P NMR of P_2APA_2 (243 MHz, D_2O)

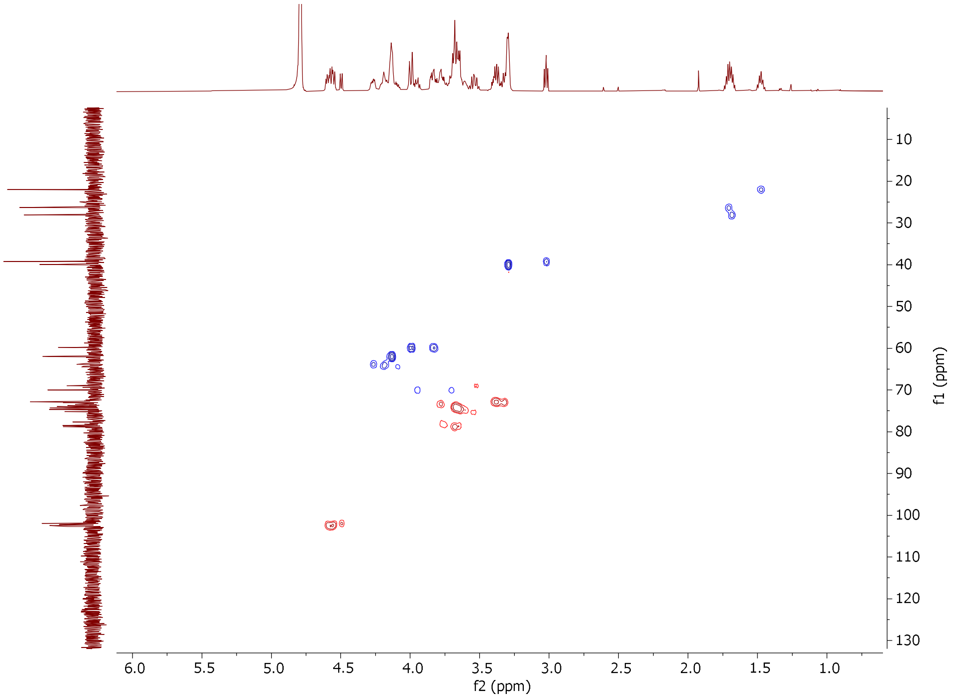

Supplementary Figure 40. HSQC NMR of P_2APA_2 (D_2O)

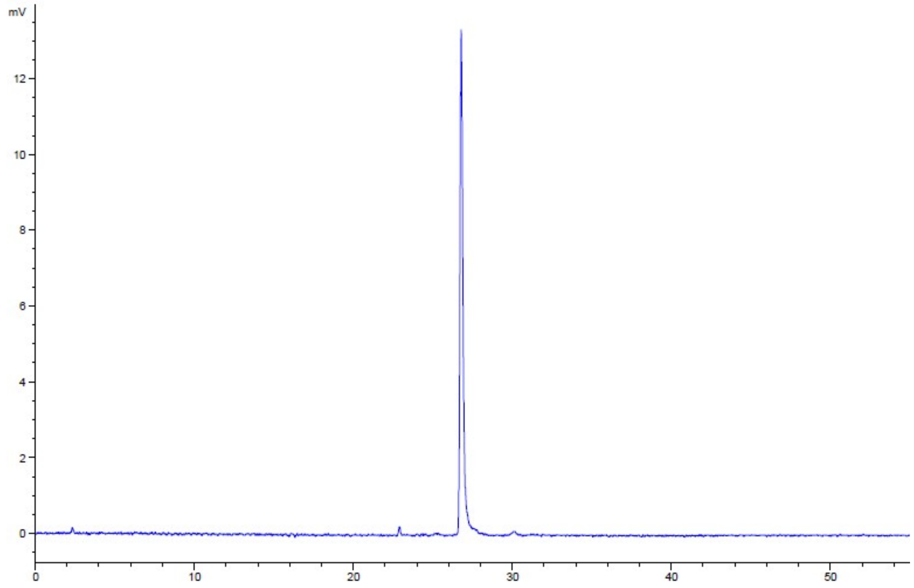

Supplementary Figure 41. RP-HPLC of P_2APA_2 (ELSD trace, Method F, t_R= 26.7 min)

2.13. Synthesis of (PA)$_3$

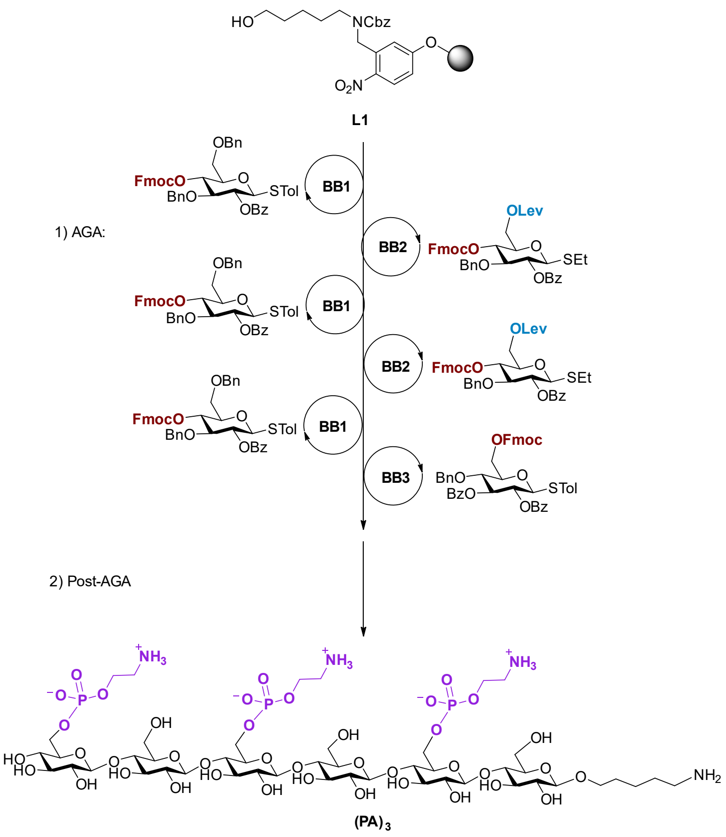

Supplementary Table 14. Synthesis of **(PA)$_3$**.

Step		Modules	Notes
AGA		**A**	
	**BB1**	**B, C, D, E1**	**C:** (**BB1**, -20 °C for 5 min, 0 °C for 20 min)
	**BB2**	**B, C, D, E1**	**C:** (**BB2**, -20 °C for 5 min, 0 °C for 20 min)
	**BB1**	**B, C, D, E1**	**C:** (**BB1**, -20 °C for 5 min, 0 °C for 20 min)
	**BB2**	**B, C, D, E1**	**C:** (**BB2**, -20 °C for 5 min, 0 °C for 20 min)
	**BB1**	**B, C, D, E1**	**C:** (**BB1**, -20 °C for 5 min, 0 °C for 20 min)
	**BB3**	**B, C, D, E1, E2**	**C:** (**BB3**, -20 °C for 5 min, 0 °C for 20 min)
Post-AGA		**F, J**	**J:** (Method **A$_2$**, t_R = 46.1 min)
		**G, J1, J2,**	**G:** (5 equiv. of **4** per -OH)<br>**J1:** (Method **B**)<br>**J2:** (Method **C**)
		**H,J1, I, J2**	**J1:** (Method **C**) **I:** 10-20% Pd(OH)$_2$/C (48 h)<br>**J2:** (Method **E** and **D**)

Compound **(PA)$_3$** was obtained as a white solid (1 mg, 5 % overall yield).

Analytical data for **(PA)$_3$:** ^{1}H NMR (600 MHz, D_2O) δ 4.63 – 4.52 (m, 5H), 4.50 (d, *J* = 8.0 Hz, 1H), 4.26 (t, *J* = 6.9 Hz, 2H), 4.18 (td, *J* = 13.8, 11.4, 8.5 Hz, 3H), 4.15 – 4.08 (m, 6H), 4.03 – 3.93 (m, 4H), 3.87 – 3.61 (m, 19H), 3.57 – 3.50 (m, 2H), 3.40 – 3.31 (m, 4H), 3.25 (m, 5H), 3.02 (t, *J* = 7.6 Hz, 2H), 1.76 – 1.64 (m, 4H), 1.47 (m, 2H)*;* ^{13}C NMR (151 MHz, D_2O) δ 102.6, 102.5, 102.4, 102.1, 101.8, 78.8, 77.7, 75.17, 74.62, 72.79, 70.1, 70.01, 68.96, 69.7, 63.25, 59.7, 40.02, 39.7, 39.29, 28.3, 26.35, 22.01, 21.8*;* ^{31}P NMR (243 MHz, D_2O) δ 0.37, 0.05, 0.03*;* m/z (HRMS+) 482.4851 $[M+3H]^{+3}$ ($C_{47}H_{94}N_4O_{40}P_3$ requires 482.4880).

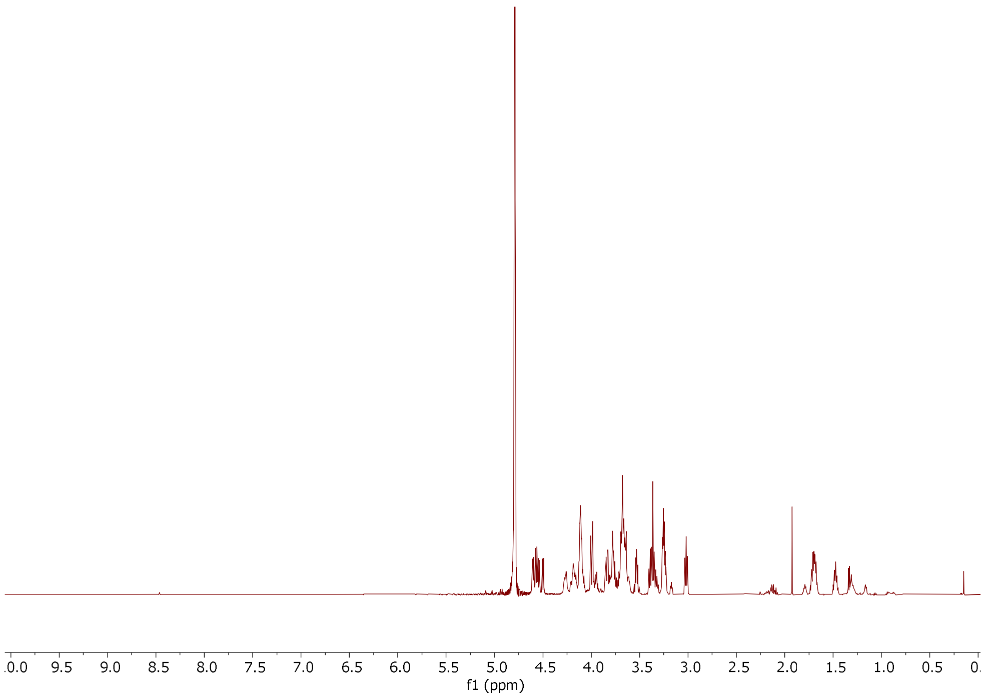

Supplementary Figure 42. ^{1}H NMR of (PA)$_3$ (600 MHz, D_2O)

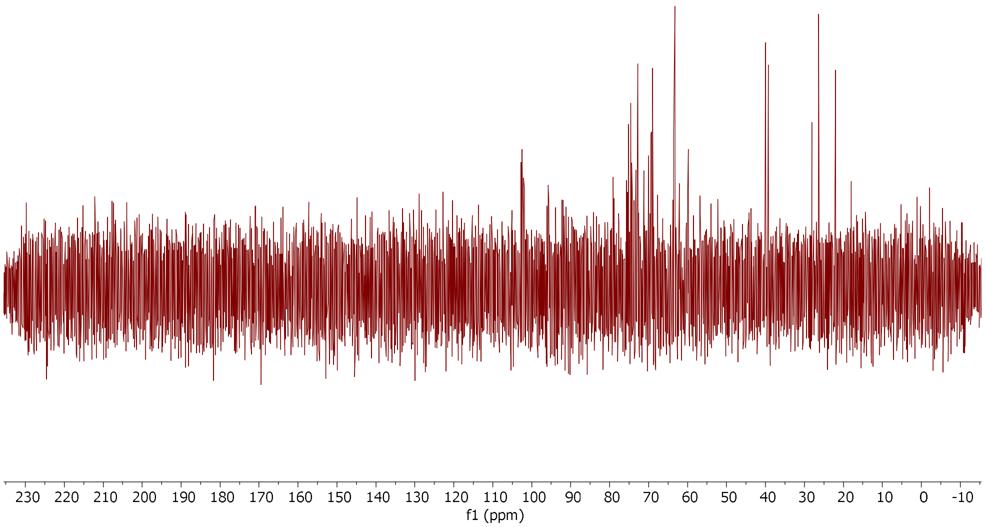

Supplementary Figure 43. ^{13}**C NMR of (PA)$_3$ (151 MHz, D$_2$O)**

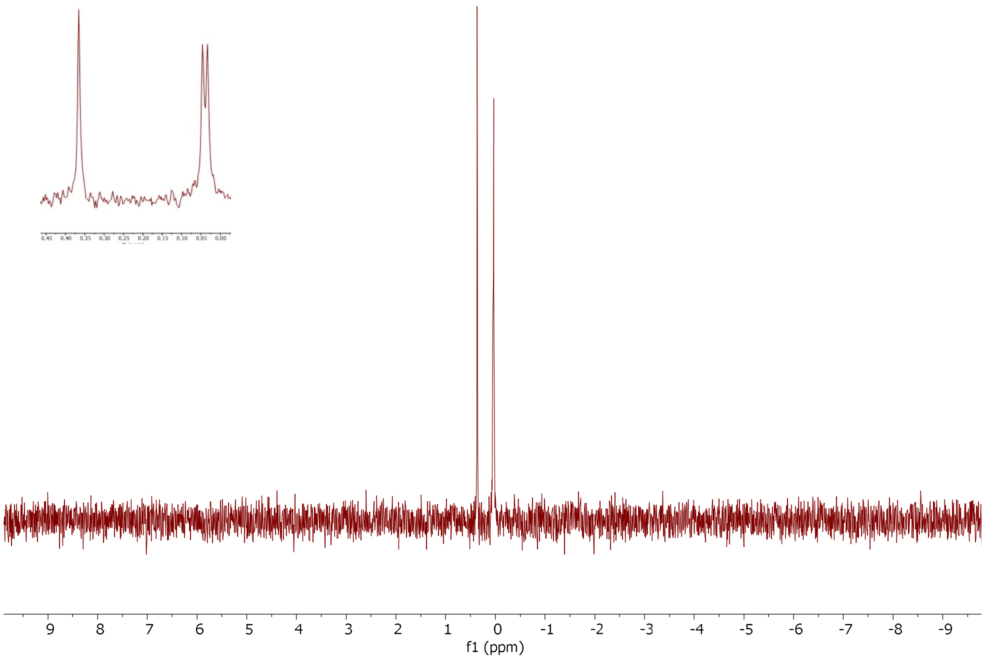

Supplementary Figure 44. ^{31}**P NMR of (PA)$_3$ (243 MHz, D$_2$O)**

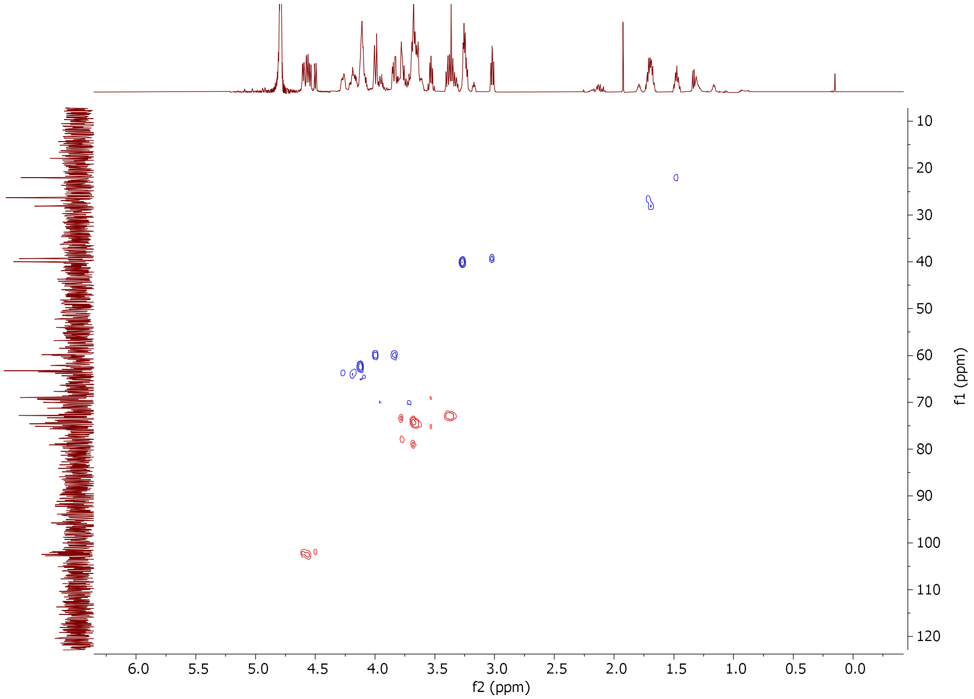

Supplementary Figure 45. HSQC NMR of (PA)$_3$ (D_2O)

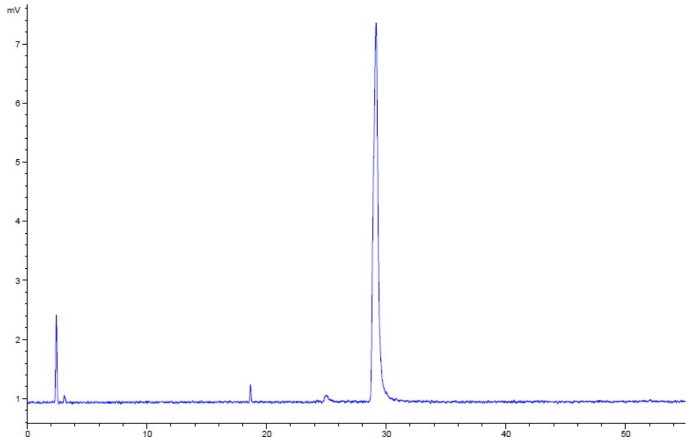

Supplementary Figure 46. RP-HPLC of (PA)$_3$ (ELSD trace, Method F, t_R= 29.1 min)

2.14. Synthesis of R5

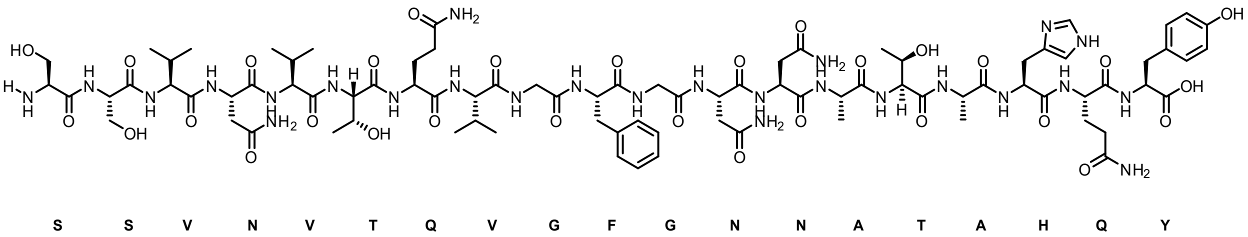

R5

The solid-phase peptides synthesis was carried out with a microwave-assisted peptide synthesizer (Liberty Blue, CEM, USA). 2-Cl-Trt-Cl Protide resin was swollen in dichloromethane for 30 min. The first amino acid was coupled manually using 4 equiv. of Fmoc-Tyr(OtBu)-OH (with respect to the resin loading) and 8 equiv. of diisopropylethylamine (DIEA) in 3 mL of DCM, shaking at RT overnight. The resin was washed with DMF and DCM. Unreacted groups were capped with a solution of DCM/CH_3OH/DIEA (17:2:1 v/v/v) (1 h at RT). Then, the resin was washed with DMF, DCM and DMF and transferred to the reaction vessel of the synthesizer. A resin loading of 0.24 mmol/g was determined using Fmoc quantification at 290 nm. Peptide coupling was performed with 0.25 M solutions of Fmoc-Xaa-COOH in DMF, 1 M ethyl cyano(hydroxyimino)acetate (Oxyma Pure®) in DMF and 0.5 M N,N′-diisopropylcarbodiimide (DIC) for activation (5 min at 75°C). The fluorenylmehtyloxycarbonyl (Fmoc) was removed with 20% piperidine in DMF solution (3 min at 75°C). Histidine coupling required a double cycle performed at RT (2 x 1 h). After the fifth residue, all coupling and deprotection cycles were performed twice. After synthesis, the resin was dried under vacuum and the peptide was cleaved by treatment with trifluoroacetic acid containing 2.5% of H_2O and 2.5% triisopropylsilane as scavengers (2 h at RT). The cleaved peptide was precipitated and washed with ice-cold diethyl ether three times and analyzed by RP-HPLC as described below. Peptide **R5** was obtained as a fluffy white powder (7 mg, 11%).

Purification

After cleavage, the crudes were analyzed with **Method G** using analytical HPLC Agilent HP 1100 and purified with **Method H**. The final pure compound was analyzed by LC-MS using a Waters Acquity UPLC coupled to a Xevo Q2-XS Qtof (**Method I**).

- **Method G:** (YMC Hydrosphere C18 column 50 mm X 3.0mm, S-3 µm) flow rate of 0.5 mL/min with 5% ACN in H_2O (0.1% formic acid) as eluent [isocratic 5% (2min)], linear gradient to 70% of ACN (15 min), linear gradient to 100% ACN (3 min)]
- **Method H:** (Manual reverse phase C_{18} silica gel column chromatography) solvent system 0.1% formic acid in H_2O:MeOH, gradient from 0 to 100% MeOH.
- **Method I**: (YMC Hydrosphere C18 column 50 mm X 3.0mm, S-3 µm) flow rate of 0.5 mL/min with 5%ACN (0.1% formic acid) in H2O (0.1% formic acid) as eluent [isocratic 5% (2min)], linear gradient to 70% of CAN (0.1% formic acid) (13 min), linear gradient to 100% ACN (3 min)]

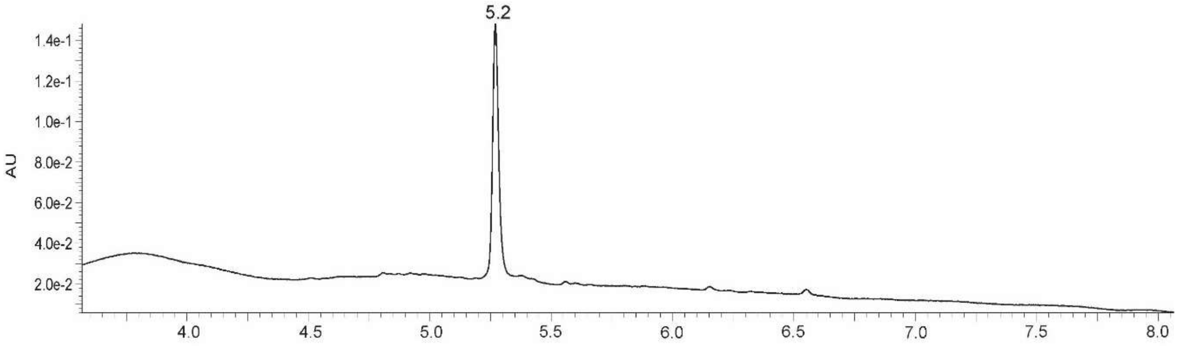

Supplementary Figure 47. RP-HPLC of R5 (UV 214nm trace, Method I, t_R= 5.2 min)

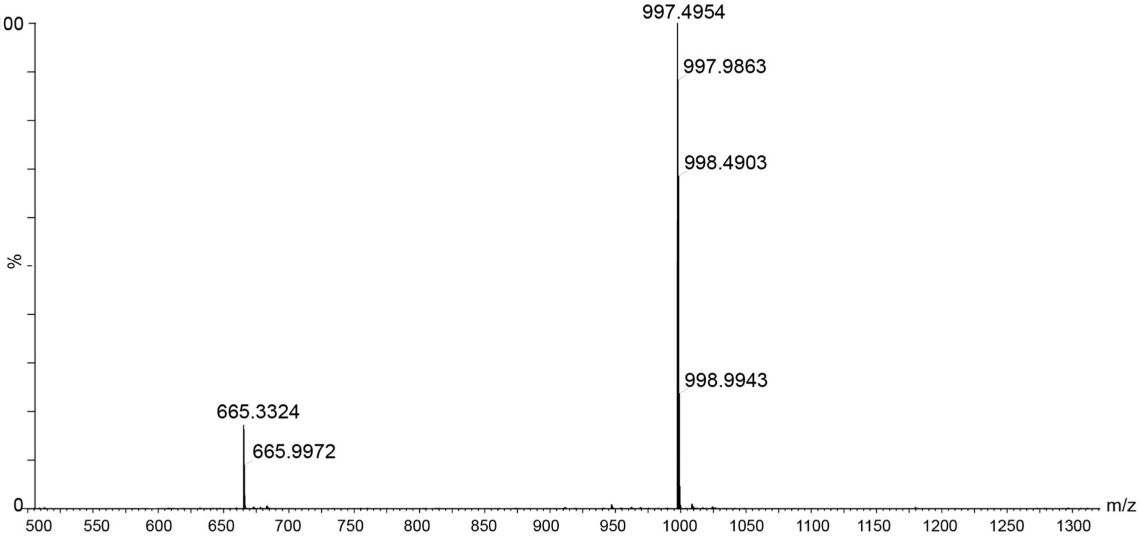

Supplementary Figure 48. ESI-HRMS of R5

3. Supplementary Discussion

3.1. Assembly of artificial fibers

Stock solutions were prepared dissolving separately **R5** and the oligosaccharides in HFIP with a concentration of 200 µM (0.4 mg mL^{-1}) and 0.13 mg mL^{-1}, respectively. The **R5** and oligosaccharide stock solutions were mixed with 2 to 1 (or 1 to 1) volume ratio to reach the final mass ratio with 6 to 1 (or 3 to 1) and sonicated for 10 minutes. HFIP was removed under gentle nitrogen purging followed by evaporation under high vacuum. Complete HFIP removal was confirmed by ^{19}F-NMR. Water was added to the dried films to reach the final peptide concentration of 25 µM for imaging, CD, ThT binding test, and AFM force measurement, and 200 µM for 2D TOCSY NMR analysis. AFM imaging and force measurement were performed in air in an AFM chamber with a relative humidity (RH) of 25%. If not mentioned, the standard ratio between **R5** and oligosaccharide is 6 to 1 by mass.

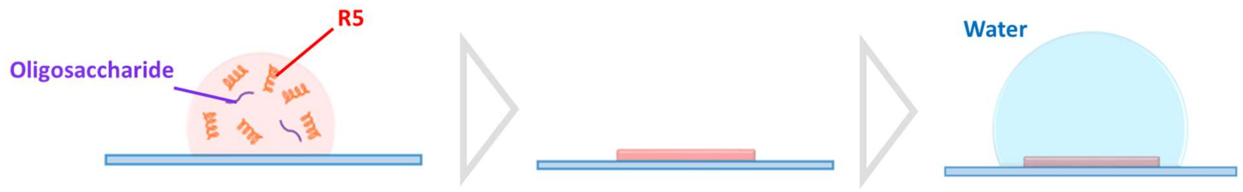

Supplementary Figure 49. Cartoon representation of the sample preparation method.

3.2. Fibrils structural analysis

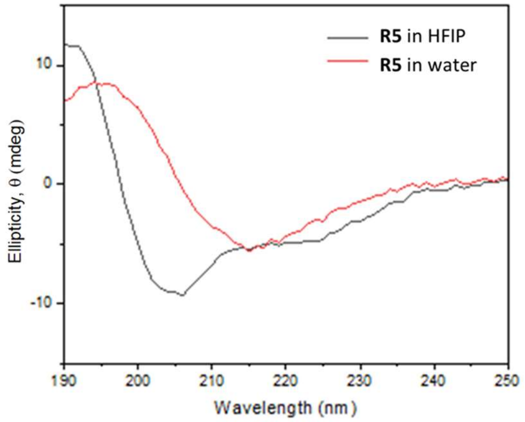

Supplementary Figure 50. CD spectra of **R5** in HFIP (black) adopting an alpha-helix structure and in water (red) adopting a beta-sheet structure (25 µM, 23 °C).

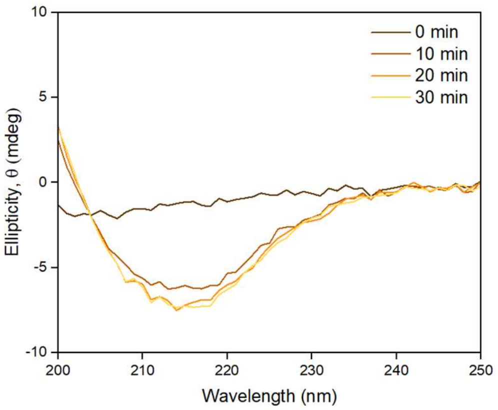

Supplementary Figure 51. CD spectra of **R5** in PBS buffer (1X, pH 7.4) confirming that **R5** showed the same secondary structure in water (neutral pH) and PBS buffer. Due to the strong background signal of the PBS buffer solution from 190 to 200 nm, the spectra were obtained from 200 to 250 nm. To avoid artefacts originated from buffer solutions and obtain clear images, all the samples in the following experiments were prepared in water.

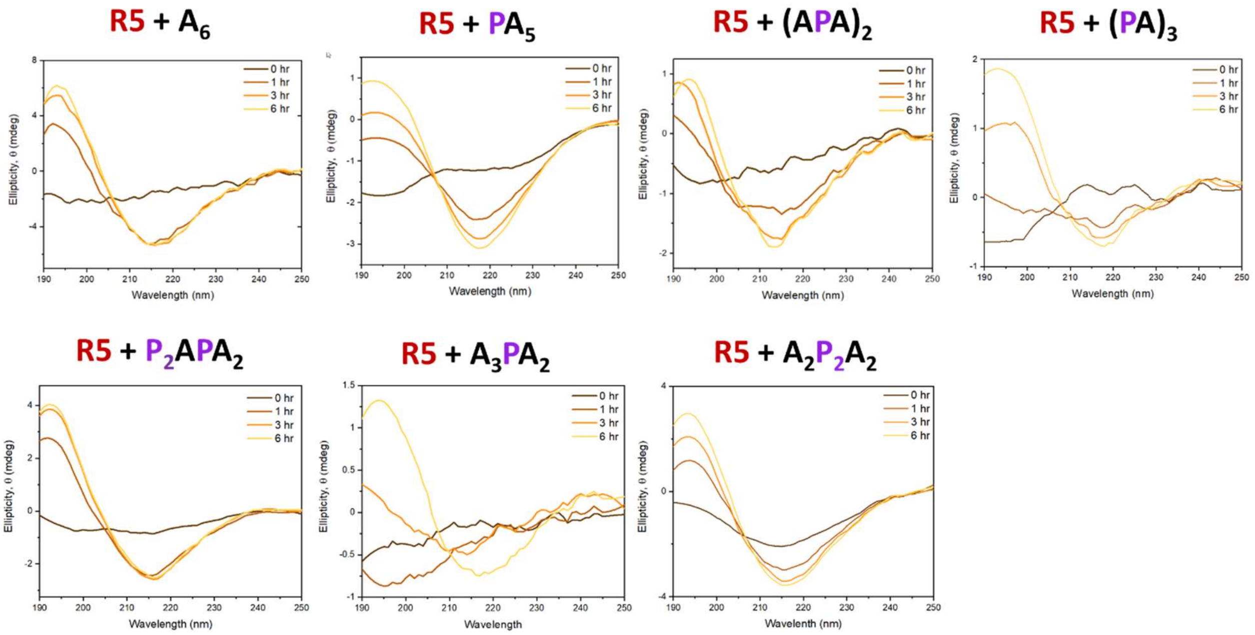

Supplementary Figure 52. CD spectra of **R5** in the presence of hexasaccharides with different degrees and patterns of pEtN substitution.

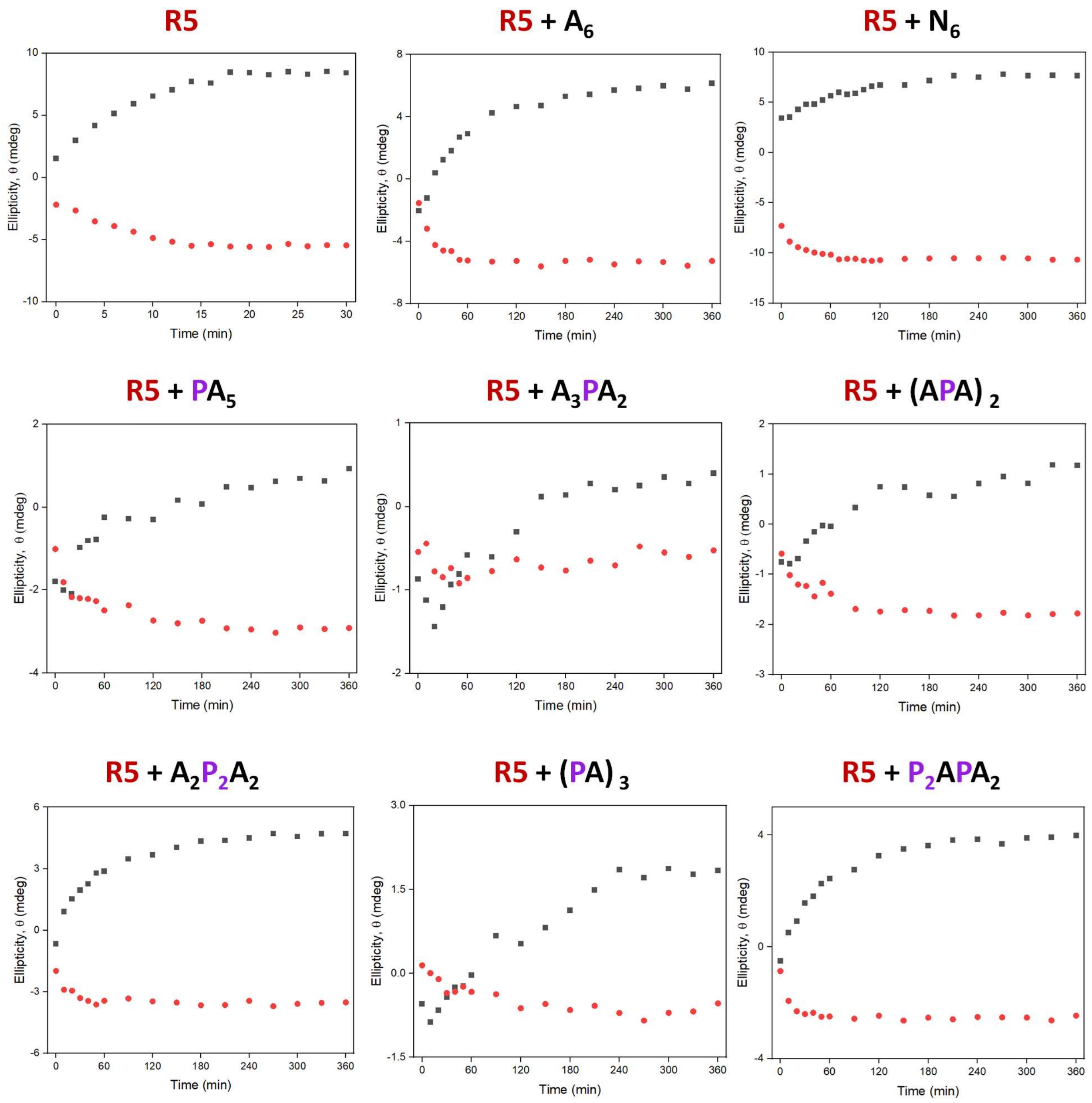

Supplementary Figure 53. Time-dependent conformational changes of **R5** in the presence of different hexasaccharides monitored by CD at $\lambda_{194\ nm}$ (black) and $\lambda_{215\ nm}$ (red). While the structural transition of **R5** alone reached a plateau within 20 minutes, longer times are required in the presence of the hexasaccharides.

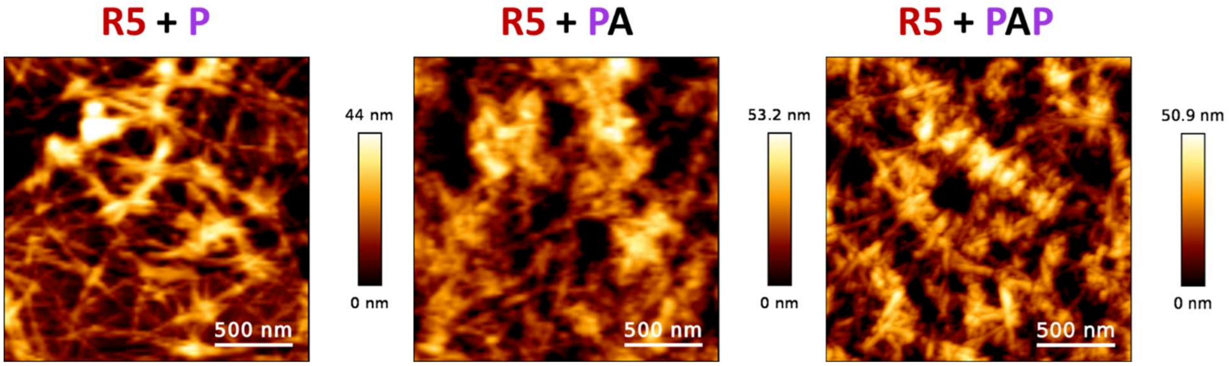

Supplementary Figure 54. AFM images of the matrix formed by **R5** in the presence of different oligosaccharides, P, PA and PAP, upon 5 days incubation (25 µM, milliQ water, RT).

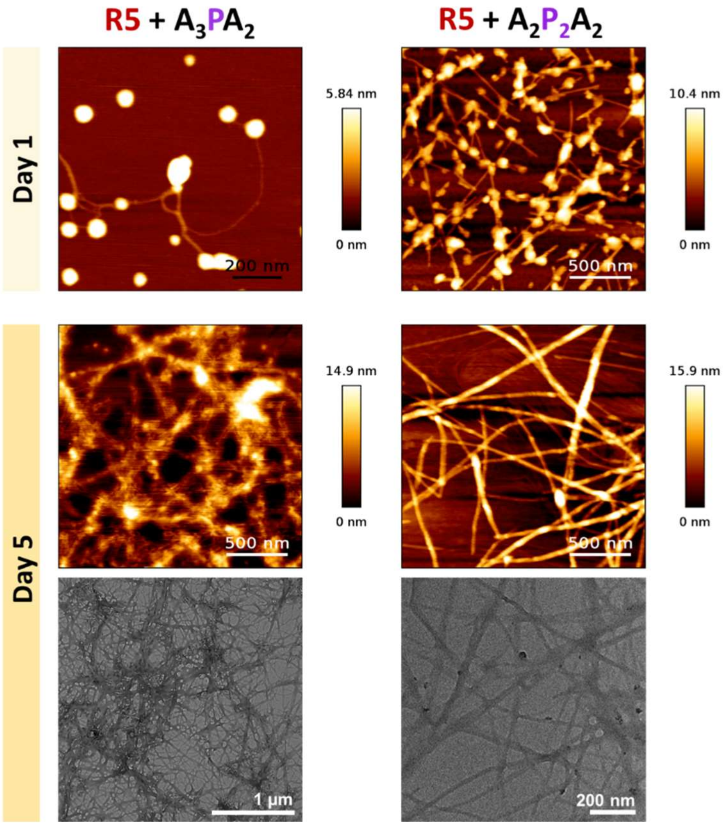

Supplementary Figure 55. AFM and TEM images of the fibers formed by **R5** in the presence of different hexasaccharides upon 1 and 5 days incubation (25 µM, milliQ water, RT).

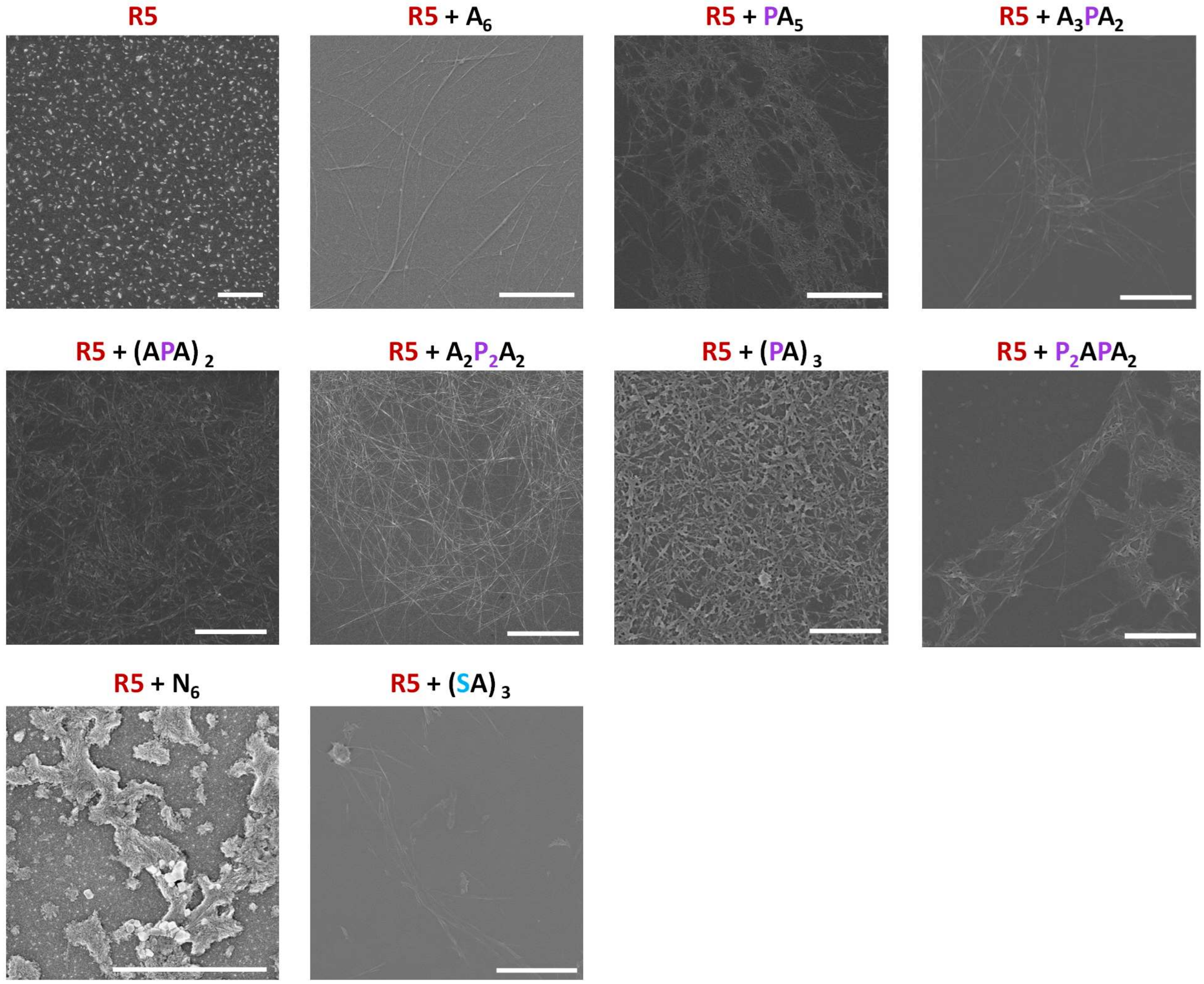

Supplementary Figure 56. SEM images of the fibers formed by **R5** in the presence of different hexasaccharides upon 5 days incubation (Scale bars: 2 µm).

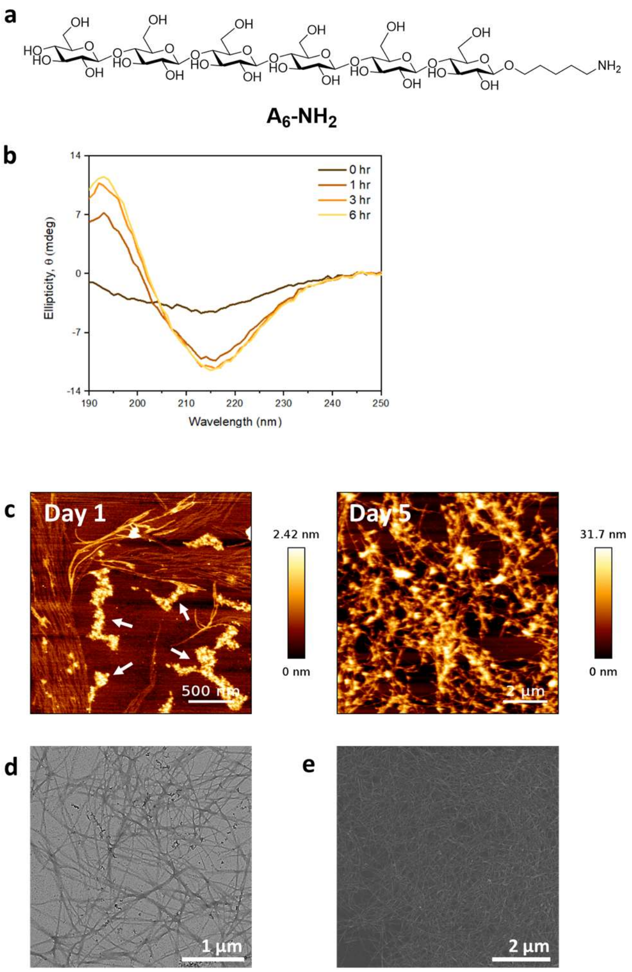

Supplementary Figure 57. (a) Chemical structure of **A_6-NH_2**. Structural transition of **R5** in the presence of **A_6-NH_2** observed via (b) CD spectra and (c) AFM, (d) TEM, and (e) SEM images of the assembled fibers to confirm that the aminopentyl linker at the reducing end of the oligosaccharide has a negligible effect on the aggregation tendency. The parts of the sample highlighted with white arrows in Supplementary Figure 10c (left) indicate the random aggregation of **A_6-NH_2**.

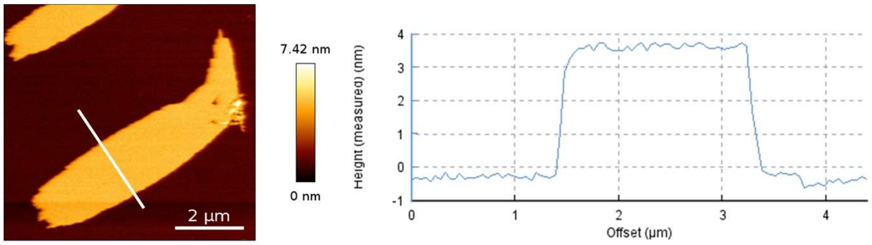

Supplementary Figure 58. AFM image and cross-sectional analysis of planar aggregates of **A_6** in the absence of **R5**.

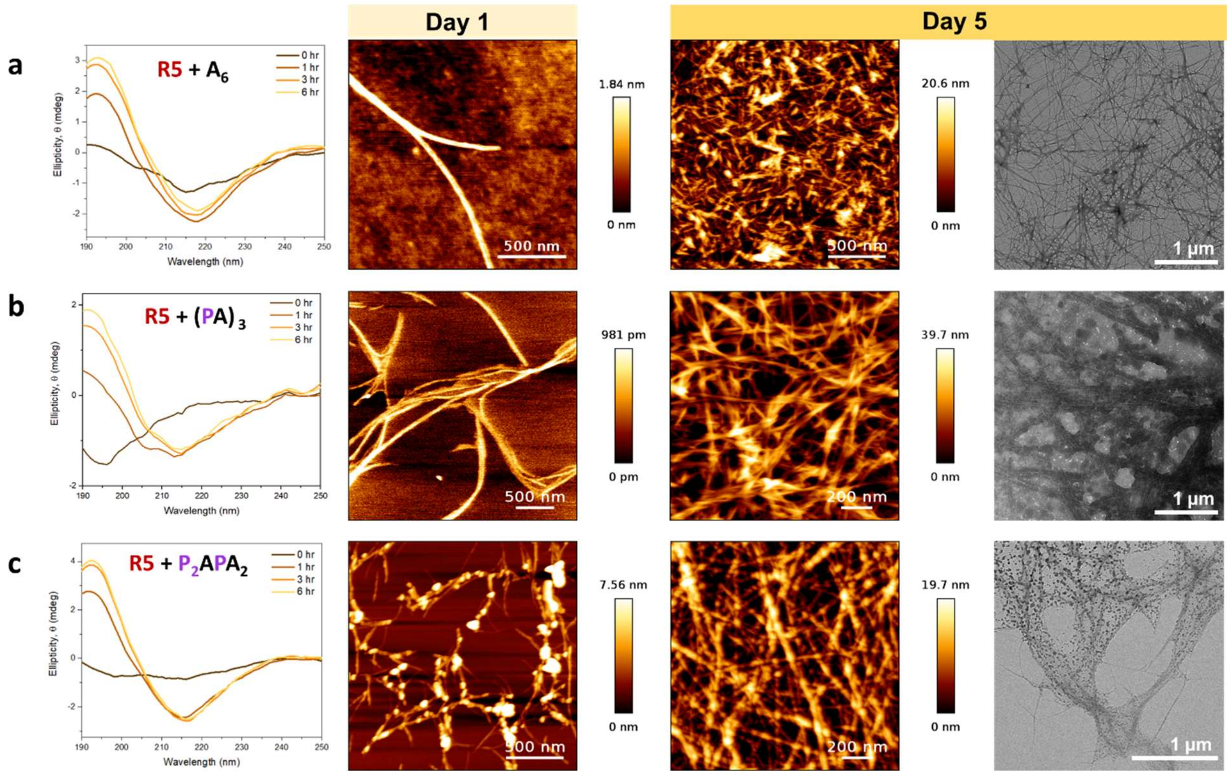

Supplementary Figure 59. CD spectra (left) and AFM and TEM images (right) of **R5** in the presence of $\mathbf{A_6}$ (a), $\mathbf{(PA)_3}$ (b), and $\mathbf{P_2APA_2}$ (c) with a 3:1 ratio by mass. TEM showed darker images compared to the samples prepared with a 6:1 mass ratio, suggesting a thicker matrix surrounding the peptide fibers.

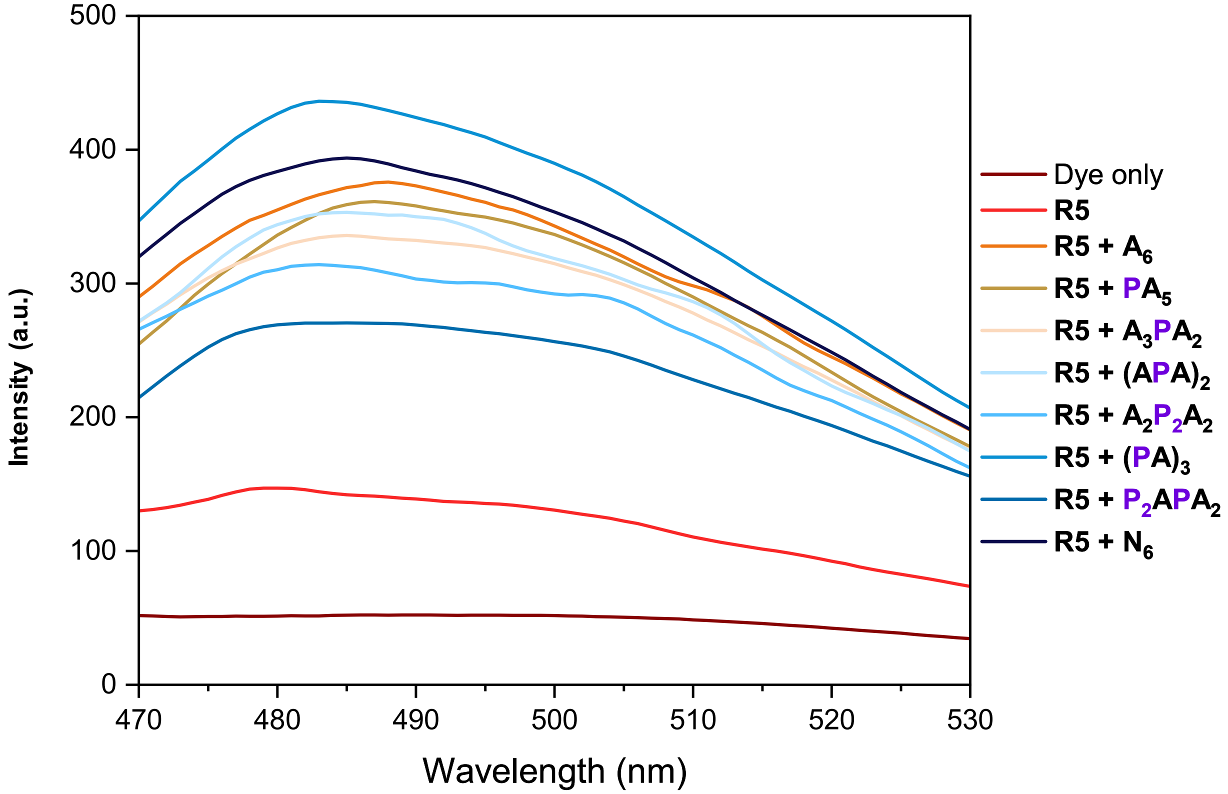

Supplementary Figure 60. ThT emission spectra to test the presence of amyloid fibers in the **R5** samples incubated for 5 days with/without different hexasaccharides, showing maxima at around 482 nm[9].

3.3. NMR sample preparation

R5 was suspended in HFIP at 0.5 mg/mL and sonicated for 1 h at RT until it was fully dissolved. **A_6**, **$(PA)_3$** and **P_2APA_2** were separately dissolved in HFIP at 0.07 mg/mL and sonicated for 1 h at RT. The solution of **R5** was mixed with the respective solution of oligosaccharide (to reach the final ratio of 6:1 by mass) and HFIP was removed by evaporation. The mixture was dried under high vacuum for 16 h before being dissolved in H_2O/D_2O (9:1, **R5** at about 400 µM). Four samples were prepared with the following concentration:

Supplementary Table 15. Summary of concentration used for NMR characterization.

	Conc. of **R5** (µM)	Conc. of oligosaccharide (µM)
**R5**	400	n.a.
**R5** and **A_6**	400	128
**R5** and **$(PA)_3$**	468	107
**R5** and **P_2APA_2**	442	102

3.4. Proton assignment and structural characterization of R5.

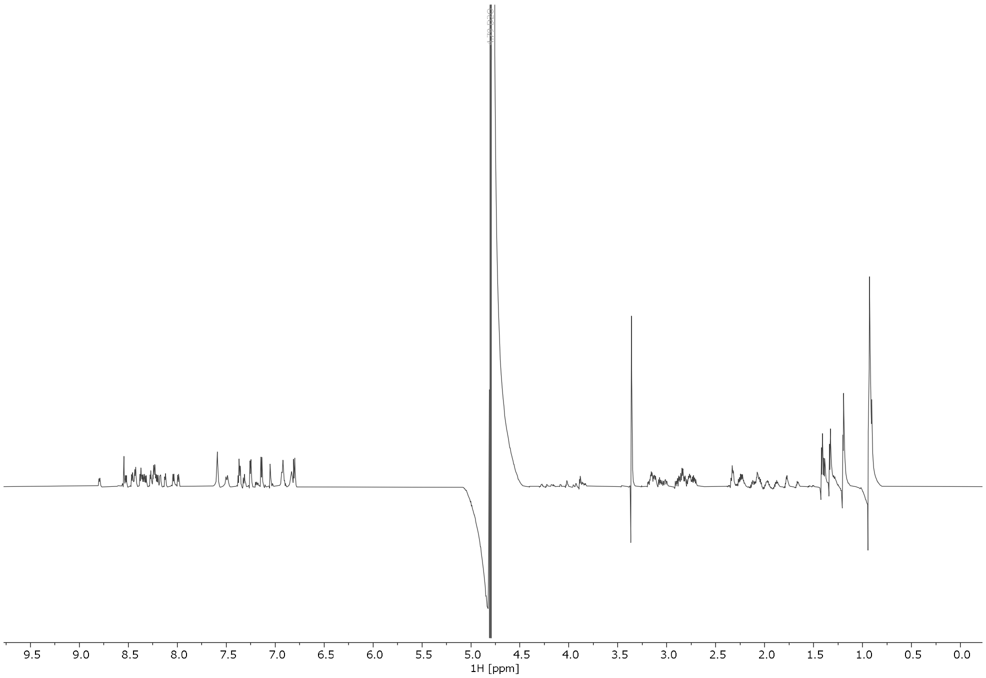

Supplementary Figure 61. ^{1}H NMR of R5 (700 MHz, H_2O/ D_2O (9:1))

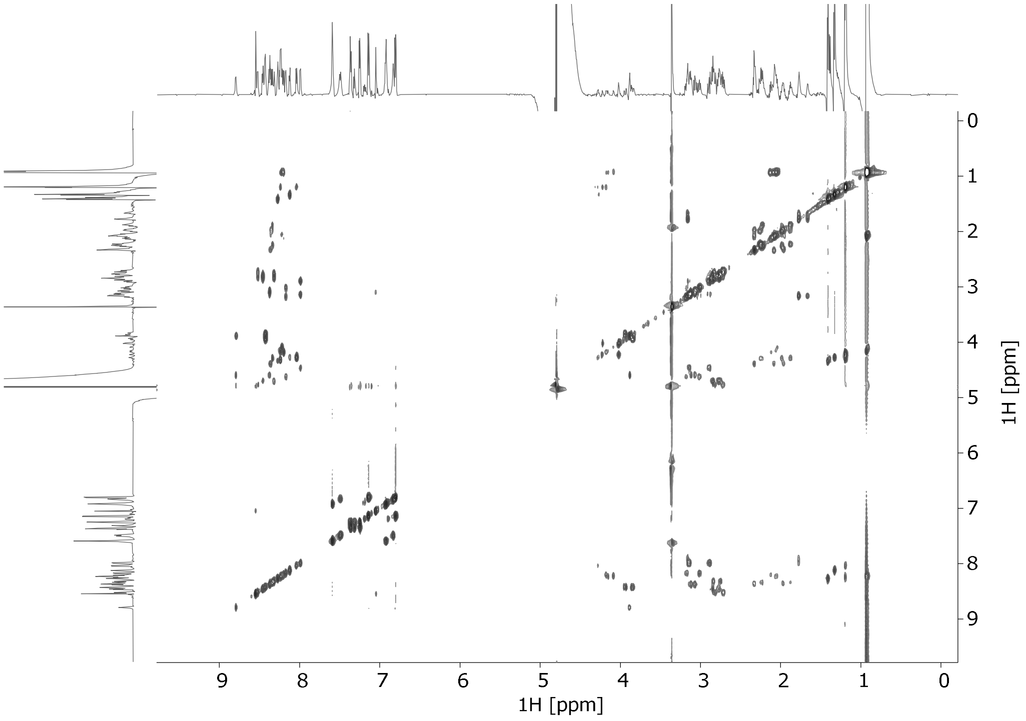

Supplementary Figure 62. ^{1}H-^{1}H **TOCSY NMR of R5**

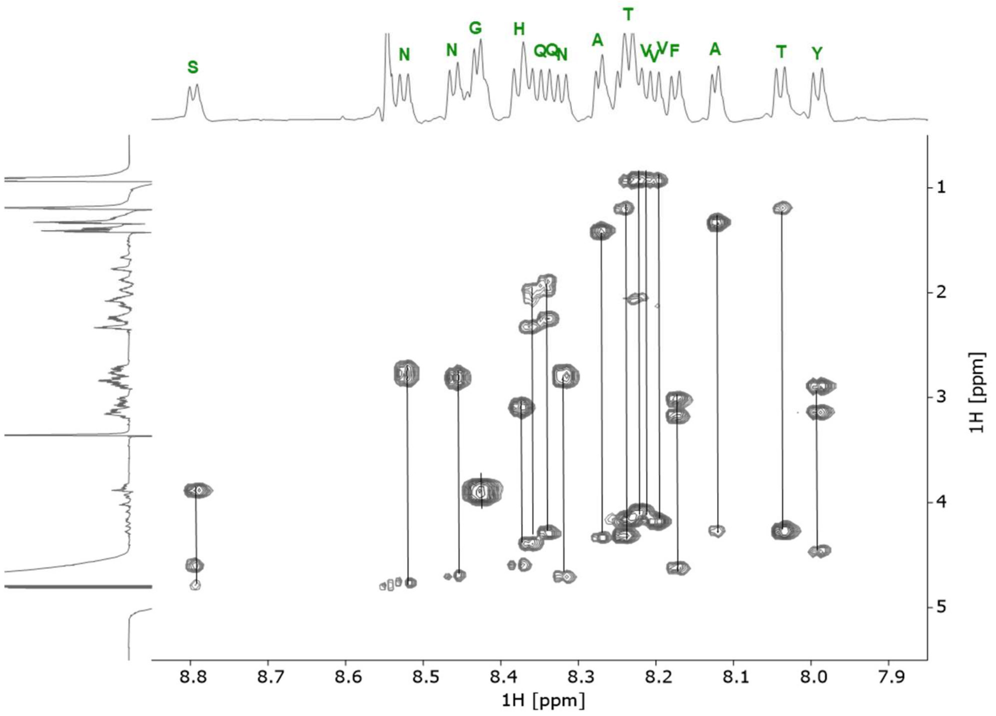

Supplementary Figure 63. Zoom of the ^{1}H-^{1}H TOCSY NMR spectrum of **R5** showing the amide resonances. The signals are assigned to the respective amino acid and labelled with the corresponding letters.

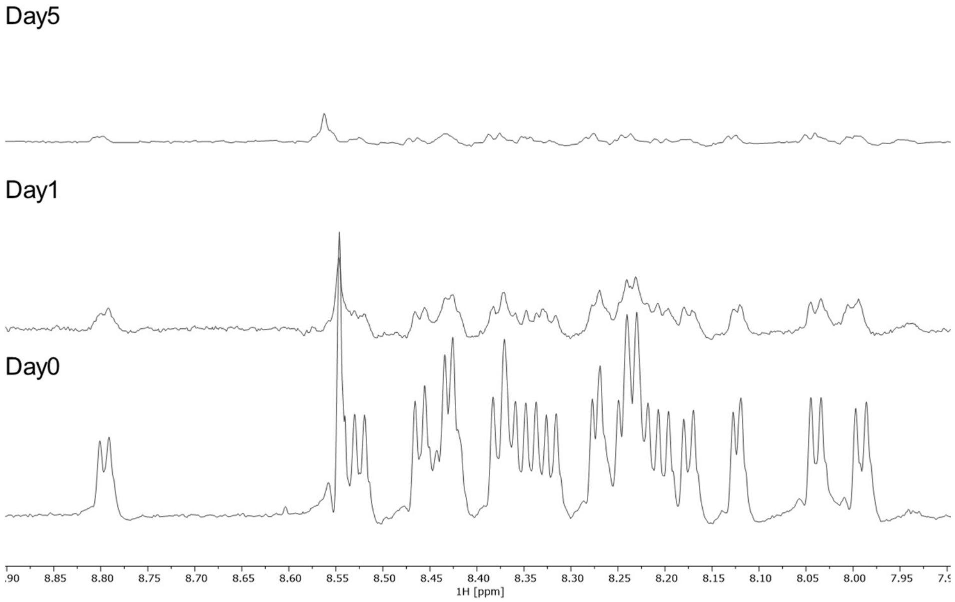

Supplementary Figure 64. ^{1}H NMR of **R5** recorded at different time intervals. Broadening of the signals and decreased intensity indicate aggregation followed by precipitation due to the formation of aggregates.

3.5. NMR characterization of R5 and A_6.

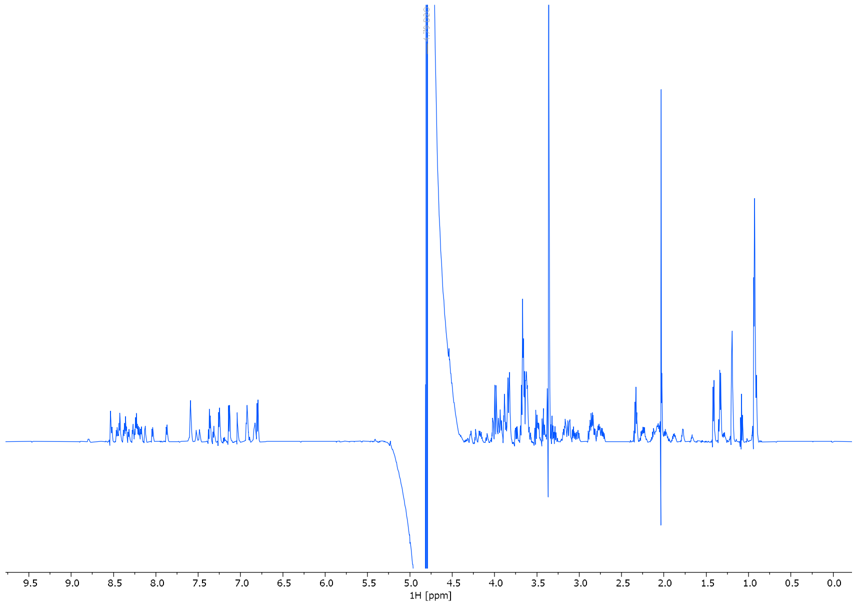

Supplementary Figure 65. ^{1}H NMR of R5 and A_6 (700 MHz, H_2O/ D_2O (9:1))

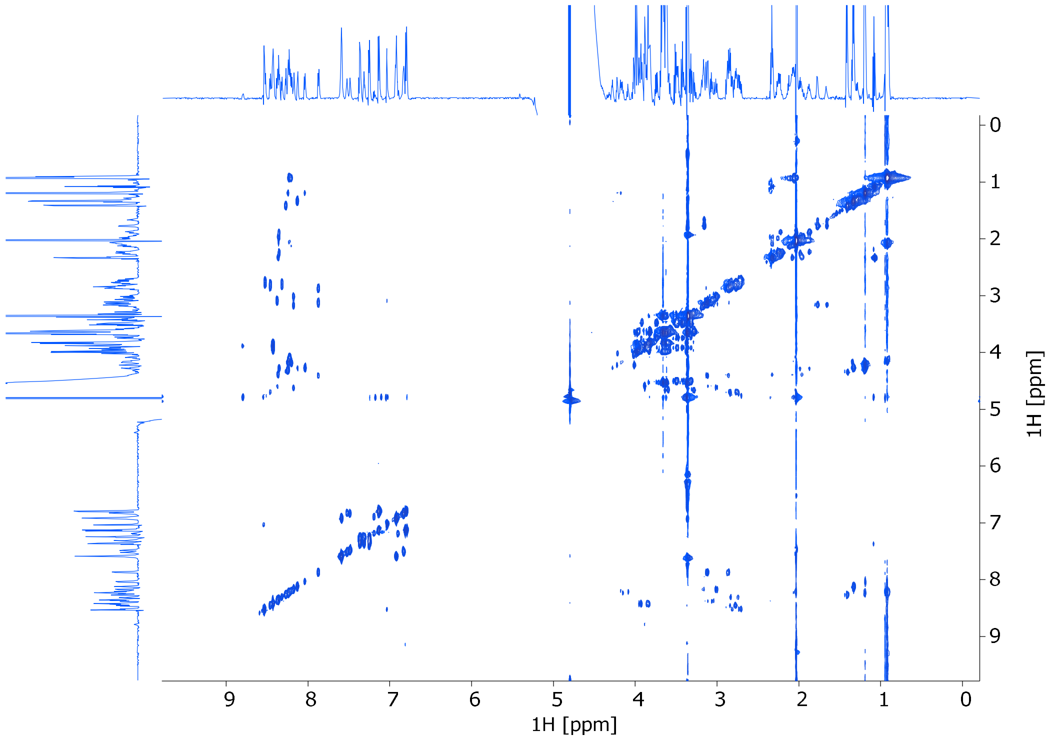

Supplementary Figure 66. ^{1}H-^{1}H TOCSY NMR of R5 with A_6

3.6. NMR characterization of R5 and $(PA)_3$.

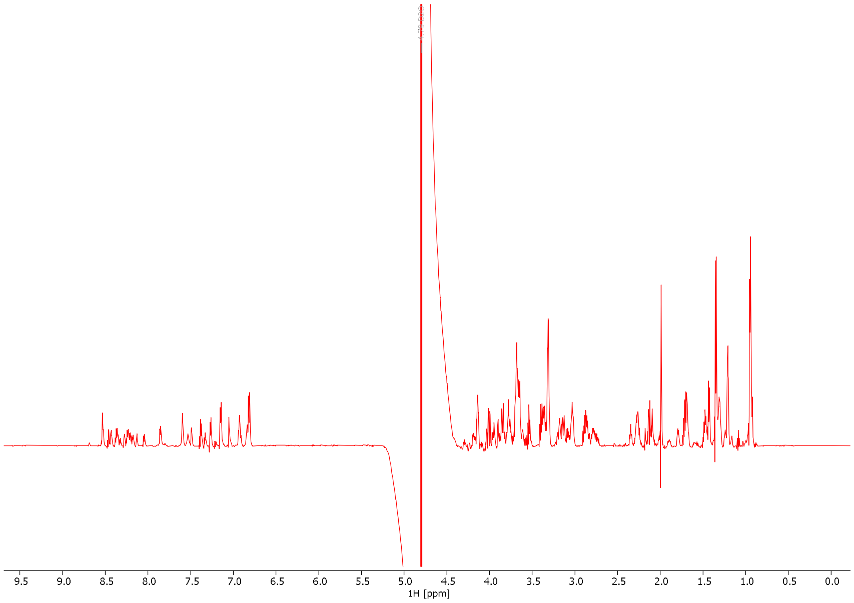

Supplementary Figure 67.^1H NMR of R5 and $(PA)_3$ (700 MHz, H_2O/ D_2O (9:1))

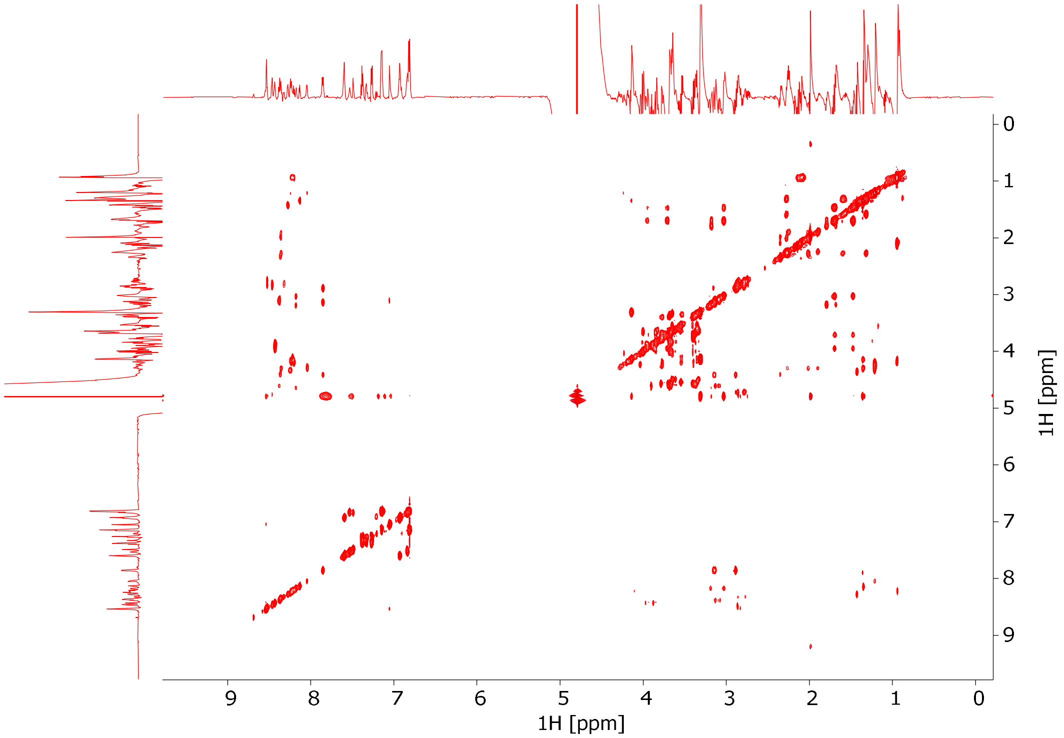

Supplementary Figure 68. ^1H-^1H TOCSY NMR of R5 with $(PA)_3$

3.7. NMR characterization of R5 and P_2APA_2.

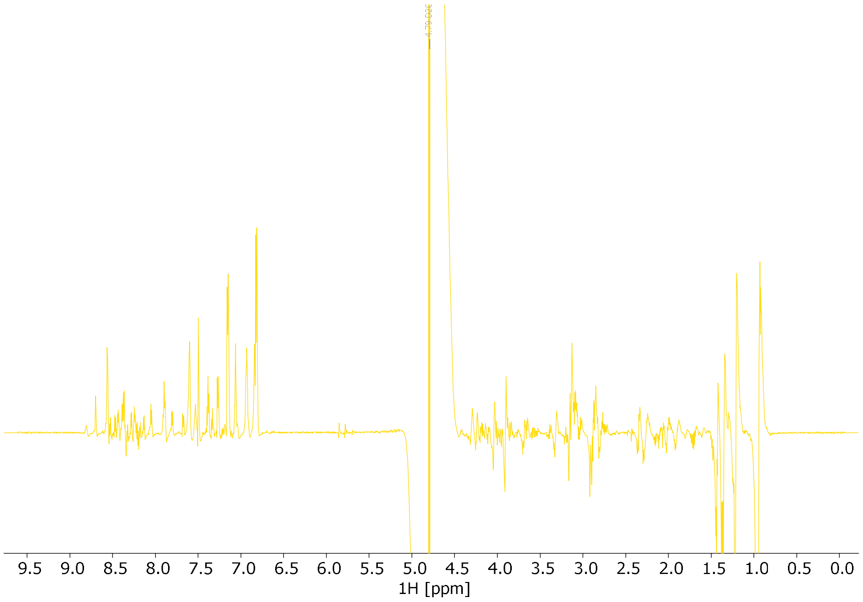

Supplementary Figure 69. ^{1}H NMR of R5 and P_2APA_2 (700 MHz, H_2O/ D_2O (9:1))

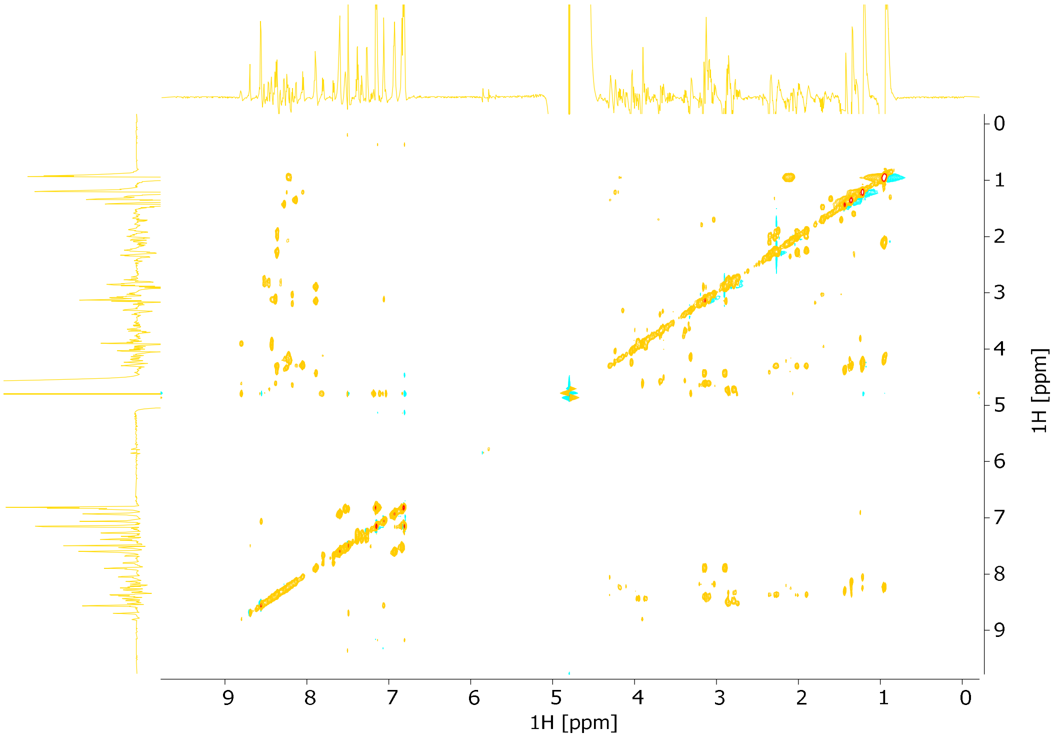

Supplementary Figure 70. ^{1}H-^{1}H TOCSY NMR and P_2APA_2

3.8. NMR comparison of the four samples.

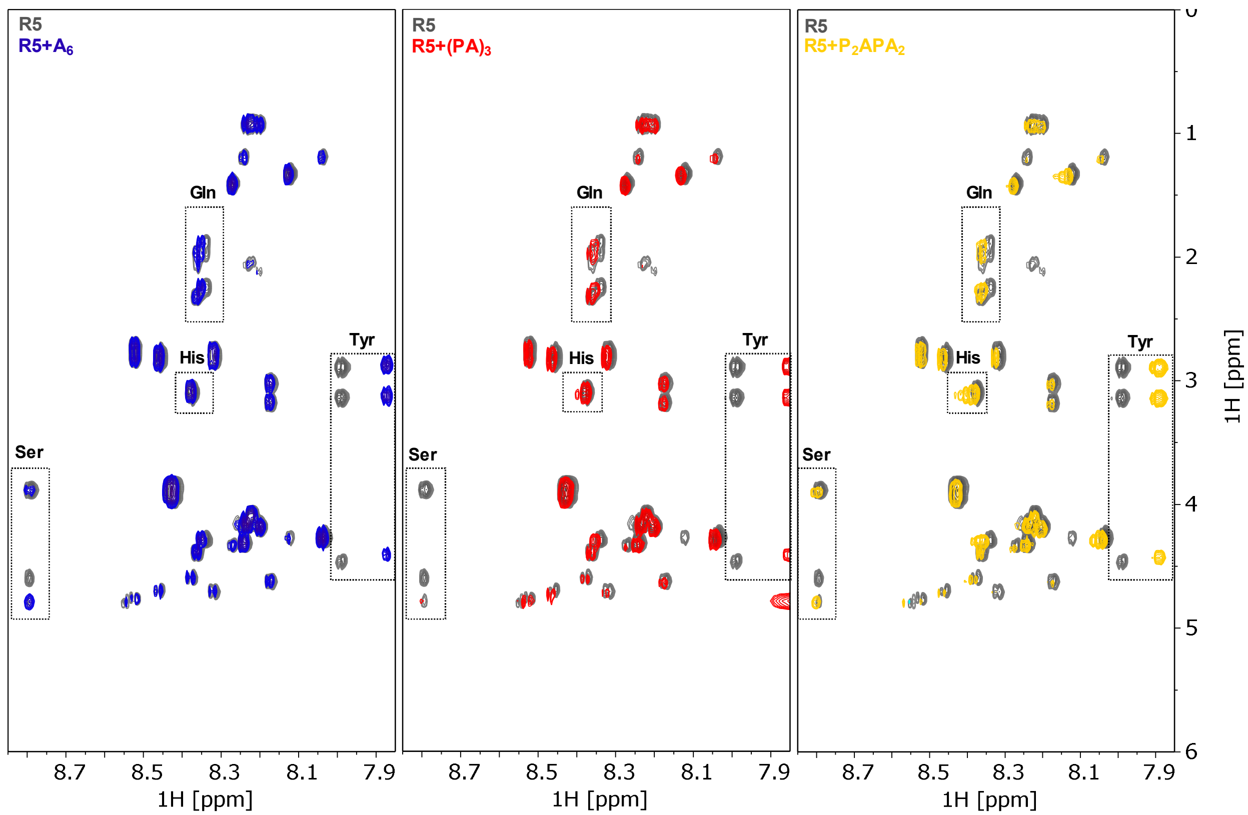

Supplementary Figure 71. Overlay of the ^{1}H-^{1}H TOCSY NMR recorded for **R5** alone (gray) and the samples in the presence of **A_6** (blue), **$(PA)_3$** (red) and **P_2APA_2** (yellow). For clarity, only the region of the amidic proton is shown.

Supplementary Table 16. Summary of the chemical shifts of the amide protons (H^N) of the affected residues measured for each sample.

Residue	R5	R5 + A_6	R5 + $(PA)_3$	R5 + P_2APA_2
	H^N (ppm)			
**Tyr**	7.99	7.87	7.86	7.89
**Gln**	8.34	8.35	8.35	8.36
**His**	8.37	8.38	8.38	8.39
**Ser**	8.79	8.79	8.80	8.80

3.9. ^{31}P NMR of (PA)$_3$ and (PA)$_3$ in presence of R5

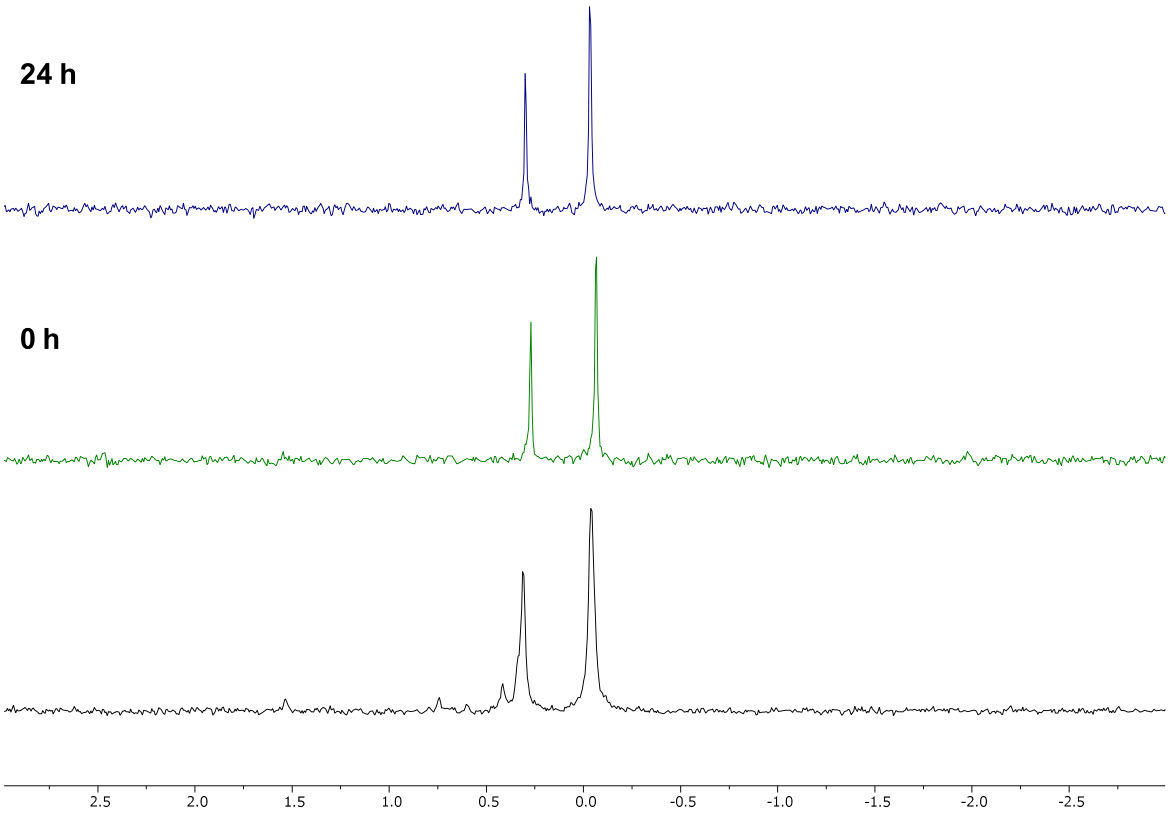

Supplementary Figure 72. ^{31}P NMR of **(PA)$_3$** (black) and (**PA)$_3$** in the presence of **R5** (green and blue) recorded at different time intervals (243 MHz, H_2O/ D_2O (9:1)).

3.10. Analysis of the mechanical properties of the artificial biofilms-inspired matrices

Supplementary Table 17. Mean Young's modulus and adhesive forces and corresponding standard deviations (SD) for the artificial films prepared by drop-casting of a 25 µM solution of the co-assembled sample to generate films with a thickness of 300 nm. The co-assembled sample were prepared with a 6:1 peptide:oligosaccharide mass ratio and incubated for 5 days before drop-casting on a pre-washed glass substrate. A minimum of three samples with 10 spots per sample was tested for each oligosaccharide. Adhesion for **R5** only could not be measured due to inhomogeneity of the film.

Sample	Stiffness (mPa)	Adhesion (nN)
Glass only	-	5.89 ± 3.59
**R5**	12.06 ± 1.42	-
**R5** + **A_6**	13.46 ± 4.81	20.06 ± 10.37
**R5** + **PA_5**	14.30 ± 3.01	105.0 ± 5.10
**R5** + **A_3PA_2**	12.69 ± 1.99	38.13 ± 18.39
**R5** + **$(APA)_2$**	12.83 ± 1.22	69.73 ± 29.11
**R5** + **$A_2P_2A_2$**	12.09 ± 2.44	22.24 ± 13.71
**R5** + **$(PA)_3$**	12.69 ± 3.11	128.9 ± 8.57
**R5** + **P_2APA_2**	12.24 ± 2.41	50.95 ± 18.47
**R5** + **N_6**	13.44 ± 1.79	40.80 ± 27.50

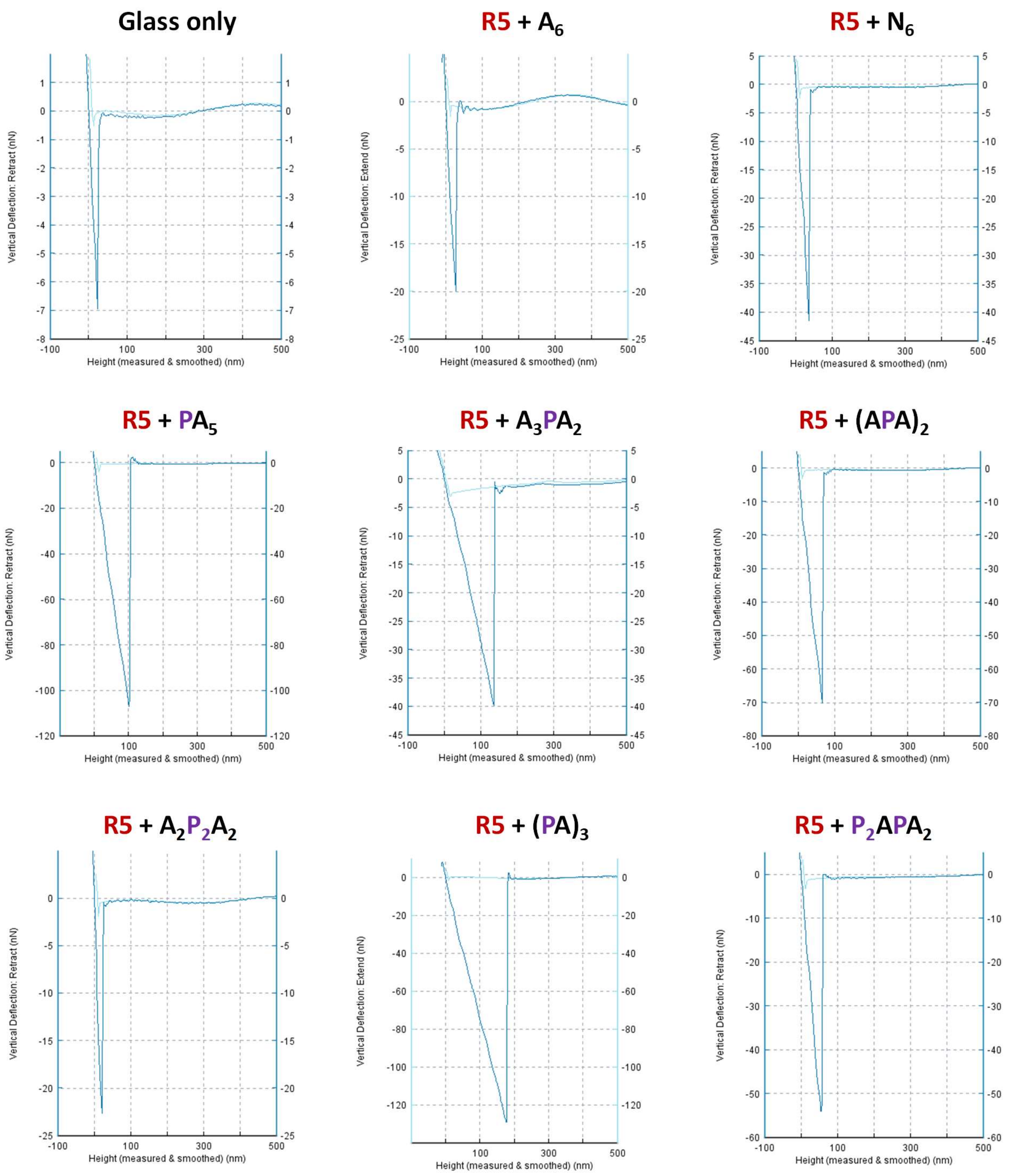

Supplementary Figure 73. Representative force-distance curves to obtain the adhesion of the artificial films with 6:1 (peptide: oligosaccharide) mass ratio. The measurements were performed in air.

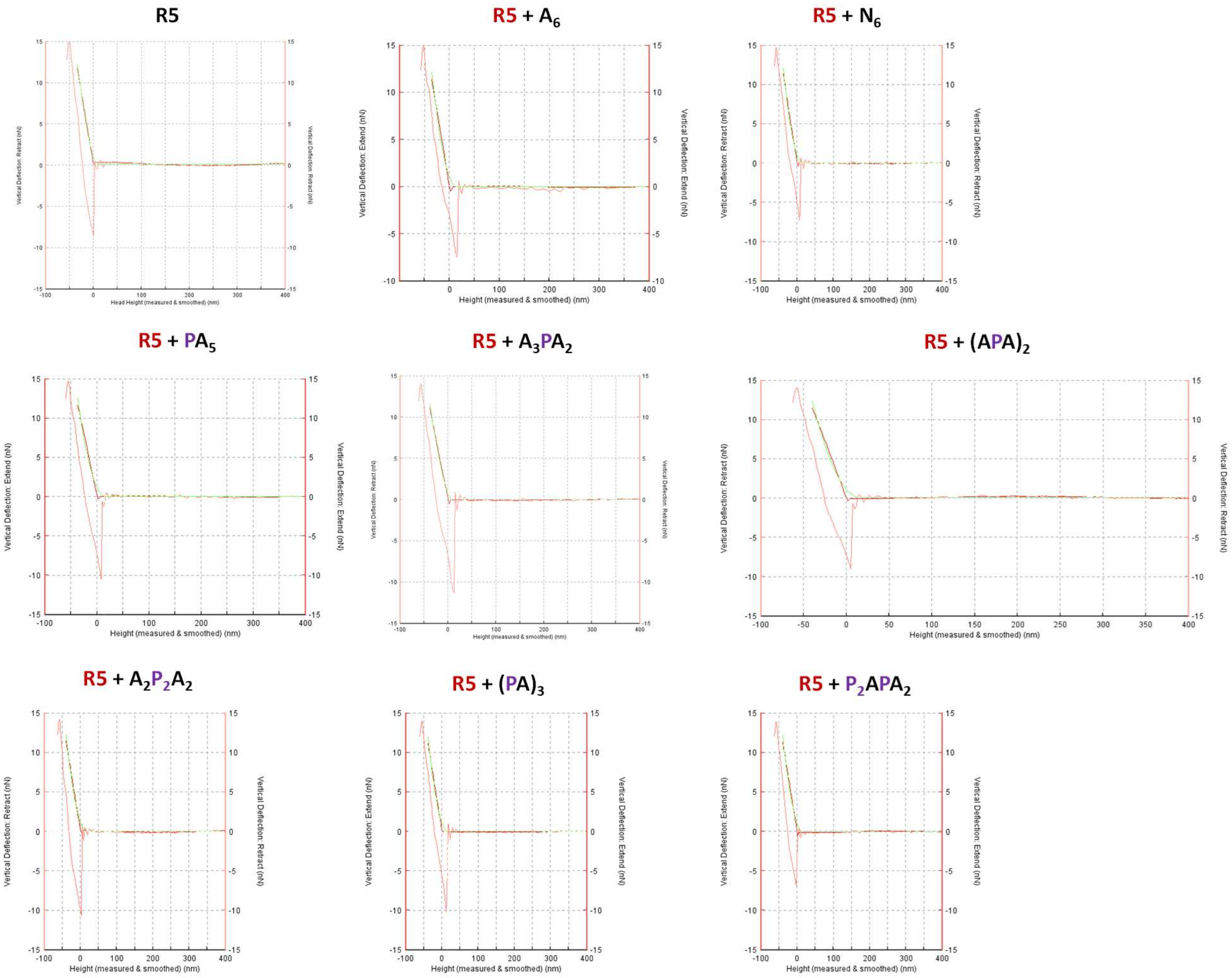

Supplementary Figure 74. Representative force-distance curves to obtain the stiffness of the artificial films with 6:1 (peptide: oligosaccharide) mass ratio. The measurements were performed in air.

4. Supplementary References

1. Yu, Y. *et al.* Systematic Hydrogen-Bond Manipulations To Establish Polysaccharide Structure–Property Correlations. *Angew. Chem. Int. Ed.* **58**, 13127–13132 (2019).

2. Tyrikos-Ergas, T. *et al.* Systematic Structural Characterization of Chitooligosaccharides Enabled by Automated Glycan Assembly. *Chem. Eur. J.* **27**, 2321–2325 (2021).

3. Tyrikos-Ergas, T., Sletten, E. T., Huang, J.-Y., Seeberger, P. H. & Delbianco, M. On resin synthesis of sulfated oligosaccharides. *Chem. Sci.* **13**, 2115–2120 (2022).

4. Le Mai Hoang, K. *et al.* Traceless Photolabile Linker Expedites the Chemical Synthesis of Complex Oligosaccharides by Automated Glycan Assembly. *J. Am. Chem. Soc.* **141**, 9079–9086 (2019).

5. Gude, M., Ryf, J. & White, P. D. An accurate method for the quantitation of Fmoc-derivatized solid phase supports. *Lett. Pept. Sci.* **9**, 203–206 (2002).

6. Guberman, M., Bräutigam, M. & Seeberger, P. H. Automated glycan assembly of Lewis type I and II oligosaccharide antigens. *Chem. Sci.* **10**, 5634–5640 (2019).

7. Kröck, L. *et al.* Streamlined access to conjugation-ready glycans by automated synthesis. *Chem. Sci.* **3**, 1617 (2012).

8. Murakata, C. & Ogawa, T. A total synthesis of GPI anchor of trypanosoma brucei. *Tetrahedron Lett.* **32**, 671–674 (1991).

9. Biancalana, M. & Koide, S. Molecular mechanism of Thioflavin-T binding to amyloid fibrils. *Biochim. Biophys. Acta* **1804**, 1405–1412 (2010).
